# Synthesis of Boron-Containing Primary Amines

**DOI:** 10.3390/molecules181012346

**Published:** 2013-10-08

**Authors:** Sheng-Hsuan Chung, Ting-Ju Lin, Qian-Yu Hu, Chia-Hua Tsai, Po-Shen Pan

**Affiliations:** Department of Chemistry, Tamkang University, No. 151 Yingzhuan rd., Tamsui Dist., New Taipei City 25137, Taiwan

**Keywords:** boron, multicomponent reactions, Gabriel synthesis, Ugi reaction, peptoid

## Abstract

In this study, boron-containing primary amines were synthesized for use as building blocks in the study of peptoids. In the first step, Gabriel synthesis conditions were modified to enable the construction of seven different aminomethylphenyl boronate esters in good to excellent yields. These compounds were further utilized to build peptoid analogs via an Ugi four-component reaction (Ugi-4CR) under microwave irradiation. The prepared Ugi-4CR boronate esters were then successfully converted to the corresponding boronic acids. Finally, the peptoid structures were successfully modified by cross-coupling to aryl/heteroaryl chlorides via a palladium-mediated Suzuki coupling reaction to yield the corresponding derivatives in moderate to good yields.

## 1. Introduction

**Figure 1:**
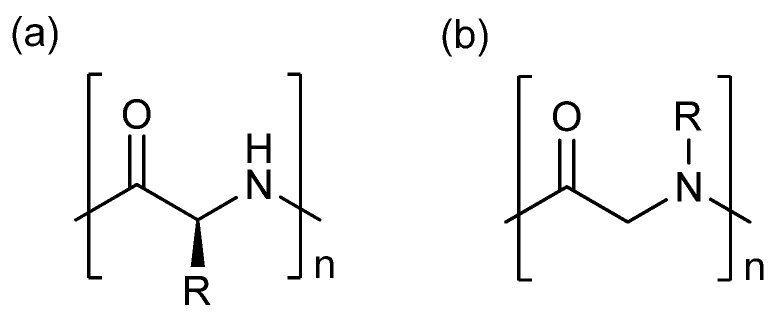
Structures of (**a**) peptide; (**b**) peptoid.

**Scheme 1:**
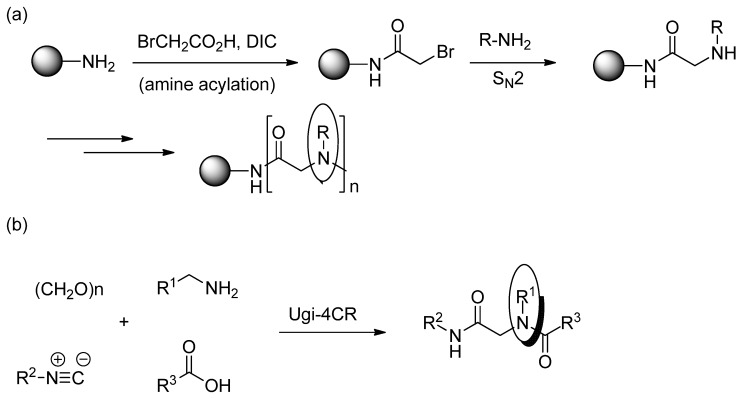
(**a**) Zuckermann peptoid synthesis; (**b**) Ugi-4CR peptoid synthesis.

Peptides are a form of naturally occurring polymer [Figure 1(a)], which have many significant pharmacological properties such as high specificity, high potency toward their target [1], and low accumulation in the body [2]. As a result, many drugs currently on the market are based on the peptide structure, including vancomycin (Vancocin^TM^), oxytocin (Oxytocin^TM^), enfuvirtide (Fuzeon^TM^), and eptifibatide (Integrilin^TM^). However, peptides also have a number of drawbacks when used as pharmaceuticals, including low cell permeability [3] and vulnerability to proteolytic degradation [4,5]. Peptoids, oligo-N-substituted glycines [Figure 1(b)], are a class of biomimetic molecule designed to mimic peptides and proteins, whilst providing the advantages of protease resistance [6] and improved cell permeability [7]. These features have driven research into the application of peptoids as therapeutic agents. Recent examples include transcription factor mimics [8], protein–protein interaction inhibitors [9], antimicrobial agents [10,11], and lung surfactant mimics [12]. There are two major strategies used for synthesizing peptoids. The first of these is the two stage sub-monomer method developed by Zuckermann and co-workers [13]. The protocol proceeds via acylation of an amine with an activated ester of 2-bromo (or chloro) acetic acid, followed by displacement of the halide with a primary amine [Scheme 1(a)]. The second strategy is via the Ugi four-component reaction [Ugi-4CR, Scheme 1(b)] [14,15], where a carboxylic acid, an isocyanide, an aldehyde (or ketone), and a primary amine are reacted in a one-pot method to generate the desired peptoid. Aside from their different synthetic approaches, both strategies rely on primary amines as the only source to introduce the side chain to the peptoid backbone. 

**Figure 2:**
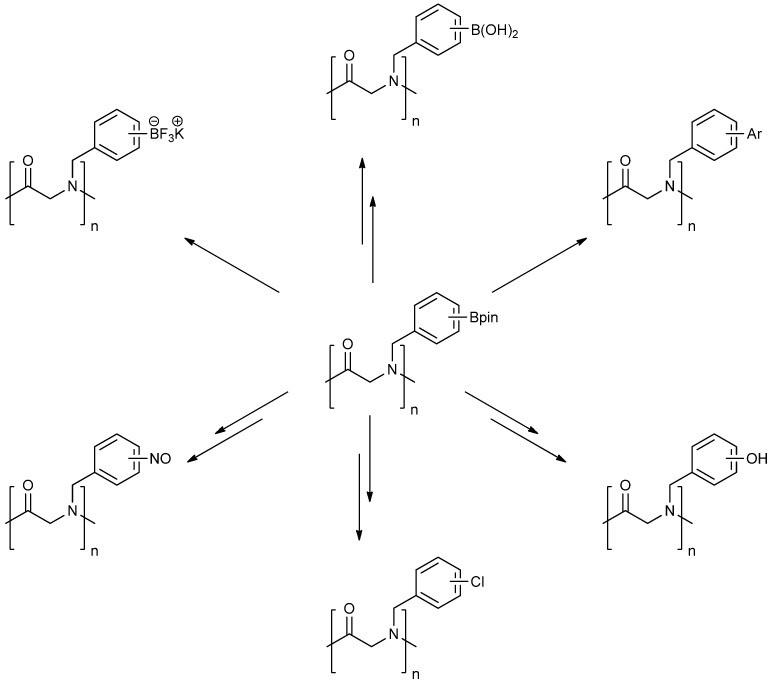
Functional group transformations from boronate ester.

Many studies have shown that both inter- and intramolecular interactions between the side chains of peptoid play a crucial role in determining its folding, conformation [16,17] and the functions [18,19]. Hence, primary amines can be used as the tool to investigate the role of individual monomer units involved in the peptoid folding/helix-stabilizing process. Boron-containing primary amines have unique properties for facilitating the study of this type of event, as they can be first incorporated into the parent peptoid backbone, and then the boron moiety can be converted into a variety of functional groups [20,21,22,23,24,25,26] (Figure 2). This transformation provides a valuable platform for evaluation of the relationships between the side chains and the peptoid folding patterns.

## 2. Results and Discussion

Although there are many synthetic methods that can be used to prepare amines [27,28], strategies for the specific synthesis of primary amines are relatively limited. The use of a reductive amination reaction is one such approach [29,30], whereby first an imine intermediate is formed and then a metal hydride reducing agent such as sodium cyanoborohydride is employed to reduce the imine double bond. The use of a protecting group is crucial when using this method in order to prevent over-alkylation, as an unprotected starting material will often result in the formation of undesired secondary or tertiary amine byproducts [31,32,33]. However, this need for the incorporation of a protecting group presents a number of disadvantages, including an increase in the total number of synthetic steps (protection and deprotection steps) and a decrease in atom economy. An alternative strategy for producing primary amines is via Gabriel synthesis [34]. In this method, the potassium salt of phthalimide is reacted with a primary alkyl halide to give the corresponding *N*-alkylphthalimide. This then reacts with hydrazine to give the desired primary amine. This synthetic strategy avoids the formation of secondary or tertiary amine byproducts without the need for a protecting group. Although this is one of the most commonly used methods, the synthesis of boron-containing primary amines via Gabriel synthesis is relatively unexplored [35]. This is partly because the reaction conditions are often harsh, and there are concerns over whether the boron functional group can survive intact in such an environment. In this report, we demonstrate the synthesis of boron-containing primary amines via a modified Gabriel synthesis.

The synthesis proceeded via the mixing of formylphenyl boronic acid with pinacol and magnesium sulfate in methanol to give the corresponding boronate ester. The progress was monitored using ^11^B-NMR spectroscopy, and when the reaction was seen to be completed, the crude solution was filtered. Sodium borohydride was then added to the filtrate, and the reaction was allowed to react at room temperature for 5 hours to afford the desired products **2a–g** in good to excellent yields (Table 1).

**Table 1:** Synthesis of hydroxymethylphenyl boronate esters.

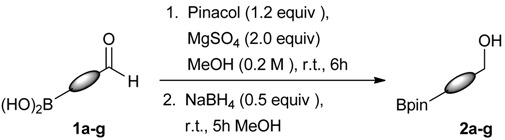			
Enrty	Aldehyde	Product	Yields (%) ^a^
1	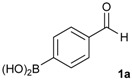	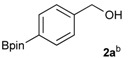	88
2	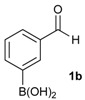	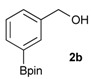	86
3	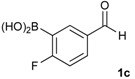	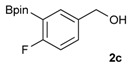	69
4	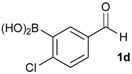	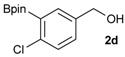	90
5	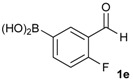	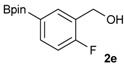	95
6	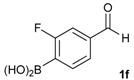	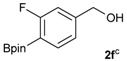	94
7	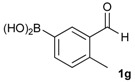	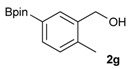	97

^a^ In two steps; ^b^ **2a** was also prepared under similar synthetic conditions [36]; ^c^ preparation of **2f** using different synthetic conditions was also reported [37].

Although THF is often used as the solvent in syntheses involving phthalimide, in the present study the desired product could not be isolated using this as either the solvent or co-solvent (Table 2, entries 1 and 2). Instead DMF was found to be the optimal solvent for this reaction (Table 2, entry 3). The use of 1.5 equiv. of potassium phthalimide gave the best yield (96%, entry 3), with a reduced amount resulting in lower yields (entries 4 and 5). In addition, six further analogs **3a–g** were successfully synthesized in moderate to excellent yields (entries 6–11).

**Table 2:** Optimization of phthalimidophenyl boronate esters synthesis.

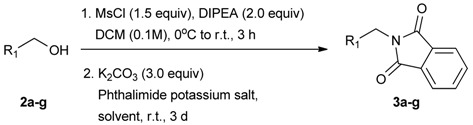					
Enrty	R^1^	Solvent	PhthK (equiv.)	Product	Yields (%)
1	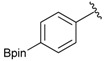	THF	**1.5**	3a	N.R.
2	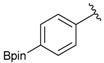	DMF/THF	**1.5**	3a	N.R.
		(v:v=1:4)			
3	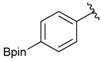	DMF	**1.5**	3a	96
4	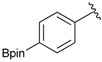	DMF	**1.2**	3a	87
5	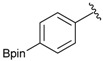	DMF	**1.0**	3a^a^	80
6	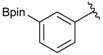	DMF	**1.5**	3b	80
7	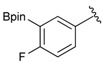	DMF	**1.5**	3c	75
8	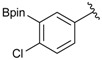	DMF	**1.5**	3d	85
9	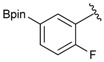	DMF	**1.5**	3e	53
10	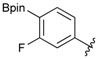	DMF	**1.5**	3f	97
11	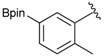	DMF	**1.5**	3g	48

^a^ **3a** was also synthesized by the similar synthetic condition [35].

The next step involved the use of the Ing-Manske procedure for the synthesis of the desired aminomethylphenyl boronate ester from the phthalimidophenyl boronate ester, and optimization of the reaction conditions. Initially, **3a** was reacted with six equivalents of hydrazine in ethanol under reflux for 8 h, giving the desired product **4a** in poor yield (Table 3, entry 1). Increasing the reaction time in addition to the amount of hydrazine used significantly improved the yield to 47% (Table 3, entry 2). 

**Table 3:** Synthesis of aminomethylphenyl boronate esters.

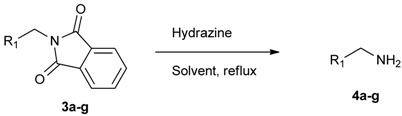						
Enrty	R^1^	Solvent	Time	Hydrazine	Product	Yields (%)
			(h)	(equiv.)		
1	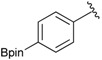	EtOH	8	6	**4a**	7
2	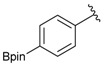	EtOH	12	8	**4a**	47
3	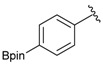	MeOH	12	3	**4a** **^a^**	73
4	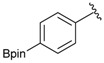	THF	12	3	**4a**	87
5	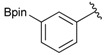	THF	12	3	**4b**	82
6	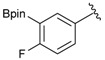	THF	12	3	**4c**	98
7	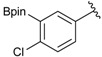	THF	12	3	**4d**	36
8	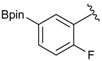	THF	12	3	**4e**	91
9	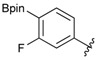	THF	12	3	**4f**	66
10	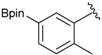	THF	12	3	**4g**	58

^a^ **4a** was also synthesized by the similar synthetic condition [35].

Other solvent systems were also investigated, and it was found that the yield was improved from 47% to 73% when methanol was used instead of ethanol (entry 3). Additionally, the use of THF improved the yield even further from 73% to 87% (entry 4). By employing these optimized conditions, **4b**–**g** were obtained in good to excellent yield (entries 5–10). Interestingly, phthalimidophenyl trifluoroborate failed to provide the desired aminomethylphenyl trifluoroborate under the same reaction conditions, due to stability issues of the boron moiety.

**Scheme 2:**
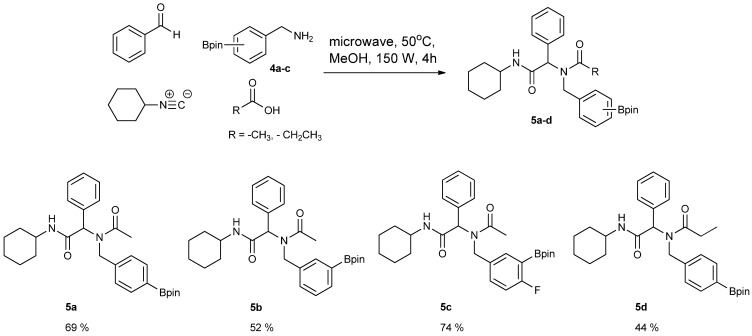
Synthesis of peptoid boronate esters via Ugi-4CR.

Three of the synthesized boron-containing primary amines **4a**–**c** were subsequently utilized as building blocks for the microwave-assisted Ugi-4CR reaction, and the desired products **5a–d** were successfully obtained in moderate to good yields (Scheme 2).

**Scheme 3:**
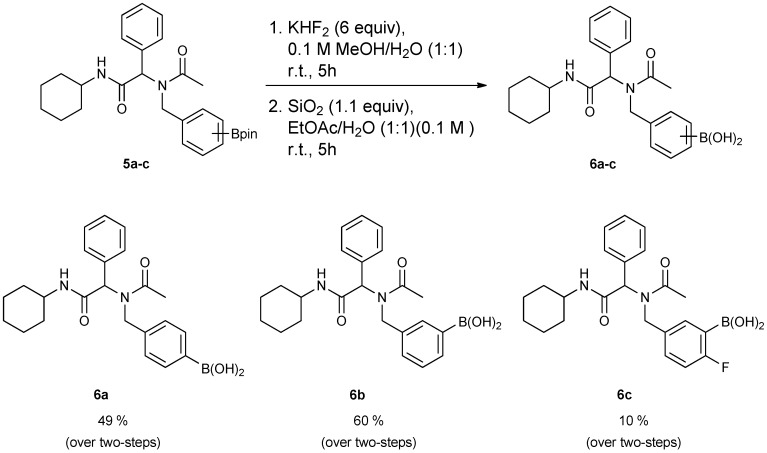
Synthesis of peptoid boronic acids from the corresponding boronate esters.

After successful synthesis of Ugi-4CR boronate esters **5a–d**, transformation into the corresponding boronic acids **6a**–**c** was performed. The boronate esters were first converted into potassium organotrifluoroborates that then underwent hydrolysis to give the desired boronic acids. Although it was possible to isolate each of the boronic acids, the substrate bearing an electron-withdrawing group **5c** gave a particularly low yield over the two steps Scheme 3.

**Scheme 4:**
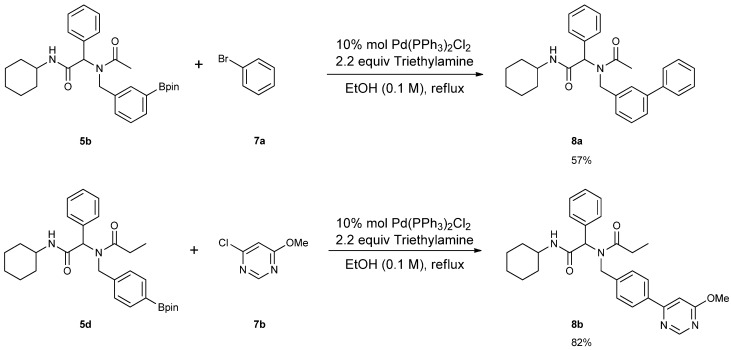
Pd mediated Suzuki cross coupling of boron containing Ugi-4CR substrate.

The structural diversity of the Ugi-4CR boronate esters was further increased by using a palladium-mediated Suzuki coupling reaction, where aryl/heteroaryl chlorides were cross-coupled to the boron- containing analogs to give **8a**–**b** in moderate to good yields (Scheme 4).

## 3. Experimental

### 3.1. General Information

All starting materials were obtained from commercial suppliers and used without further purification unless otherwise noted. Reactions were performed on a CEM Co., Discovery microwave reactor with sealed vessels. Unless otherwise specified ^1^H-, and ^13^C-NMR spectra were recorded on a Bruker AC-300 FT-NMR spectrometer at 300 and 76 MHz, respectively. ^11^B-NMR spectra were recorded on a Bruker Avance 600 FT-NMR spectrometer at 193 MHz. All ^11^B chemical shifts were referenced to external BF_3_·OEt_2_ (0.0 ppm). Data is represented as follows: chemical shifts (ppm), multiplicity: (s = singlet, d = doublet, t = triplet, m = multiplet, br = broad), coupling constant *J* (Hz). Melting points were determined by using a Fargo MP-2D melting point apparatus and were uncorrected. High resolution ESI mass spectra were obtained on a Finnigan MAT 95S instrument. The desired products **4a**–**g** were purified by a RP-HPLC using ODS-A C18 reverse phase column (5 μm, 10 mm × 250 mm) and following gradient elutions with solvent A: 0.1% TFA/ water, solvent B: 0.1% TFA/acetonitrile; from 0% to 90% of B over 90 min, a flow rate: 2.0 mL/min; detection: UV, 215 and 254 nm.

### 3.2. General Procedure A for the Synthesis of Boron-Containing Primary Alcohols ***2a**–**g***


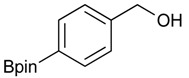


*[4-(4,4,5,5-Tetramethyl-1,3,2-dioxaborolan-2-yl)phenyl]methanol* (**2a**). To a mixture of a 4-formylbenzenboronic acid (**1a**, 375 mg, 2.50 mmol), pinacol (355 mg, 3.00 mmol) and anhydrous magnesium sulfate (625 mg, 5.00 mmol), methanol was added (12.50 mL). The mixture was stirred at room temperature for 6 h. After the reaction was completed, the crude solution was filtered, and then sodium borohydride (47 mg, 1.25 mmol) was added to the filtrate. Afterwards, the reaction mixture was stirred for an additional 5 h. Once the reaction was completed, the reaction mixture was filtered and the filtrate was concentrated *in vacuo* to give the desired product **2a** as a white solid (m.p. 75–77 °C) in 88% yield (513 mg); ^1^H-NMR (CD_3_OD-*d_4_*) δ ppm 7.71 (d, *J* = 8.0 Hz, 2H), 7.35 (d, *J* = 7.8 Hz, 2H), 4.62 (s, 2H), 1.34 (s, 12H); ^13^C-NMR (CD_3_OD-*d_4_*) δ ppm 146.23, 135.93, 127.26, 85.19, 65.24, 25.34; ^11^B-NMR (CDCl_3_) δ ppm 34.82.


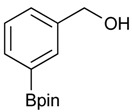


*[3-(4,4,5,5-Tetramethyl-1,3,2-dioxaborolan-2-yl)phenyl]methanol* (**2b**). Following the General Procedure A, the desired compound was synthesized utilizing 3-formylbenzenboronic acid (**1b**, 375 mg, 2.50 mmol), pinacol (355 mg, 3.00 mmol), anhydrous magnesium sulfate (601 mg, 5.00 mmol), sodium borohydride (47 mg, 1.25 mmol), and methanol (12.50 mL) giving compound **2b** as a white solid (m.p. 48–50 °C) in 86% yield (502 mg); ^1^H-NMR (CD_3_OD-*d_4_*) δ ppm 7.74 (s, 1 H), 7.64 (d, *J* = 7.2 Hz, 1H), 7.46 (d, *J* = 7.4 Hz, 1H), 7.34 (t, *J* = 7.4 Hz, 1H), 4.60 (s, 2H), 1.34 (s, 12H); ^13^C-NMR (CD_3_OD-*d_4_*) δ ppm 142.11, 134.76, 134.50, 131.23, 128.93, 85.23, 65.35, 25.35; ^11^B-NMR (CDCl_3_) δ ppm 30.97.


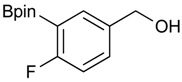


*[4-Fluoro-3-(4,4,5,5-tetramethyl-1,3,2-dioxaborolan-2-yl]phenyl)methanol* (**2c**). Following the General Procedure A, the desired compound was synthesized utilizing 2-fluoro-3-formylbenzenboronic acid (**1c**, 420 mg, 2.50 mmol), pinacol (355 mg, 3.00 mmol), anhydrous magnesium sulfate (601 mg, 5.00 mmol), sodium borohydride (47 mg, 1.25 mmol), and methanol (12.50 mL) giving compound **2c** as a white solid (m.p. 80–82 °C) in 69% yield (435 mg); ^1^H-NMR (CD_3_OD-*d_4_*) δ ppm 7.68 (t, *J* = 3.4 Hz, 1H), 7.49–7.44 (m, 1H), 7.01 (t, *J* = 6.5 Hz, 1H), 4.57 (s, 2H), 1.34 (s, 12H); ^13^C-NMR (CD_3_OD-*d_4_*) δ ppm 166.42 (d, *J* = 249.0 Hz), 136.7, 135.1, 135.0, 131.9, 131.9, 114.7, 114.5, 114.2, 83.7, 62.9, 23.7, 23.7, 23.6; ^11^B-NMR (CDCl_3_) δ ppm 30.2.


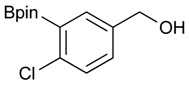


*[4-Chloro-3-(4,4,5,5-tetramethyl-1,3,2-dioxaborolan-2-yl)phenyl]methanol* (**2d**). Following the General Procedure A, the desired compound was synthesized utilizing 2-chloro-5-(hydroxymethyl)phenylboronic acid (**1d**, 242 mg, 1.30 mmol), pinacol (184 mg, 1.56 mmol), anhydrous magnesium sulfate (313 mg, 2.60 mmol), sodium borohydride (25 mg, 0.65 mmol), and methanol (6.50 mL) giving compound **2d** as a oil in 90% yield (314 mg); ^1^H-NMR (CD_3_OD-*d_4_*) δ ppm 7.66 (d, *J* = 1.8 Hz, 1H), 7.39–7.31 (m, 2H), 4.57 (s, 2H), 1.39 (t, *J* = 18.1 Hz, 12H); ^13^C-NMR (CDCl_3_) δ ppm 138.4, 138.1, 134.5, 130.2, 129.2, 84.0, 63.7, 24.5, 24.3; ^11^B-NMR (CDCl_3_) δ ppm 30.6.


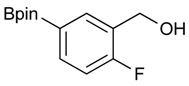


*[2-Fluoro-5-(4,4,5,5-tetramethyl-1,3,2-dioxaborolan-2-yl)phenyl]methanol* (**2e**). Following the General Procedure A, the desired compound was synthesized utilizing 4-fluoro-3-formylphenylboronic acid (**1e**, 218 mg, 1.30 mmol), pinacol (184 mg, 1.56 mmol), anhydrous magnesium sulfate (313 mg, 2.60 mmol), sodium borohydride (25 mg, 0.65 mmol), and methanol (6.50 mL) giving compound **2e** as a white solid (m.p. 44–47 °C) in 95% yield (312 mg); ^1^H-NMR (CD_3_OD-*d_4_*) δ ppm 7.87 (d, *J* = 8.1 Hz, 1H), 7.71–7.09 (m, 1H), 7.05 (t, *J* = 10.4 Hz, 1H), 4.67 (s, 2H), 1.34 (s, 12H); ^13^C-NMR (CD_3_OD-*d_4_*) δ ppm 164.4(d, *J* = 250.5 Hz), 137.6, 137.2, 137.1, 129.2, 129.1, 115.8, 115.7, 85.4, 58.9, 25.3, 25.2; ^11^B-NMR (CDCl_3_) δ ppm 30.6.


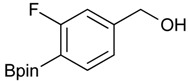


*[3-Fluoro-4-(4,4,5,5-tetramethyl-1,3,2-dioxaborolan-2-yl)phenyl]methanol* (**2f**). Following the General Procedure A, the desired compound was synthesized utilizing 2-fluoro-4-formylphenylboronic acid (**1f**, 218 mg, 1.30 mmol), pinacol (184 mg, 1.56 mmol), anhydrous magnesium sulfate (313 mg, 2.60 mmol), sodium borohydride (25 mg, 0.65 mmol), and methanol (6.50 mL) giving compound **2f** as a white solid (m.p. 35–38 °C) in 94% yield (308 mg); ^1^H-NMR (CDCl_3_) δ ppm 7.60 (t, *J* = 6.8 Hz, 1H), 6.99 (d, *J* = 7.5 Hz, 1H), 6.93 (d, *J* = 10.4 Hz, 1H), 4.53 (s, 2H), 1.29 (s, 12H); ^13^C-NMR (CDCl_3_) δ ppm 167.4 (d, *J* = 250.5 Hz), 147.2, 136.9, 136.8, 121.5, 113.3, 113.1, 83.9, 64.2, 30.3, 24.8; ^11^B-NMR (CDCl_3_) δ ppm 29.9.


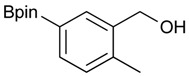


*[2-Methyl-5-(4,4,5,5-tetramethyl-1,3,2-dioxaborolan-2-yl)phenyl]methanol* (**2g**)*.* Following the General Procedure A, the desired compound was synthesized utilizing 3-formylbenzenboronic acid (**1g**, 213 mg, 1.30 mmol), pinacol (184 mg, 1.56 mmol), anhydrous magnesium sulfate (313 mg, 2.60 mmol), sodium borohydride (25 mg, 0.65 mmol), and methanol (6.50 mL) giving compound **2g** as a white solid (m.p. 60–63 °C) in 97% yield (312 mg); ^1^H-NMR (CD_3_OD-*d_4_*) δ ppm 7.74 (s, 1H), 7.55 (d, *J* = 7.4 Hz, 1H), 7.16 (d, *J* = 7.4 Hz, 1H), 4.62 (s, 2H), 2.36 (s, 3H), 1.34 (s, 12H); ^13^C-NMR (CD_3_OD-*d_4_*) δ ppm 141.1, 139.8, 135.4, 135.1, 130.8, 85.1, 63.5, 25.3, 25.2, 19.1; ^11^B-NMR (CDCl_3_) δ ppm 30.9.

### 3.3. General Procedure B for the Synthesis of Boron-Containing Primary Phthalimides ***3a–g***


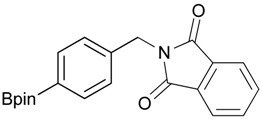


*2-[4-(4,4,5,5-Tetramethyl-1,3,2-dioxaborolan-2-yl)benzyl]isoindoline-1,3-dione* (**3a**). Compound **2a** (585 mg, 2.50 mmol), and dichloromethane (25.00 mL) were added to a dry flask containing a magnetic stir bar under a nitrogen atmosphere. The flask was cooled to 0 °C, then methanesulfonyl chloroide (0.29 mL, 3.75 mmol) and *N*,*N*-diisopropylethylamine (DIPEA, 0.87 mL, 5.00 mmol) were slowly added to the flask. The reaction mixture was stirred at 0 °C for 3 h. After the reaction was completed, the reaction mixture was diluted with dichloromethane (25.00 mL) before H_2_O (25.00 mL) was added. The organic layer was then washed with brine and dried with MgSO_4_. The resulting organic layer was then filtered and the filtrate was concentrated *in vacuo*. The resulting crude material was re-dissolved in DMF (4.68 mL) after which both potassium phthalimide salt (695 mg, 3.75 mmol), and K_2_CO_3_ (1,036 mg, 7.50 mmol) were added to the solution. The reaction was then allowed to stir at room temperature for 3 days. After the reaction was completed, the distilled H_2_O (20.00 mL) was slowly added to the reaction mixture to afford the formation of a solid precipitate. The reaction mixture was then filtered and the filtered cake was collected. The filtered cake was re-dissolved in *tert*-butanol/H_2_O (4:1, v/v) (10.00 mL) before a freeze-drying process was applied to remove the remaining DMF. The desired product **3a** was obtained as a white solid (m.p. 166–169 °C) in 96% yield (872 mg); ^1^H-NMR (CDCl_3_) δ ppm 7.84–7.82 (m, 2 H), 7.74 (d, *J* = 7.85 Hz, 2 H), 7.71–7.68 (m, 2H), 7.42 (d, *J* = 7.85 Hz, 2H), 4.85 (s, 2H), 1.34 (s, 12H); ^13^C-NMR (CDCl_3_) δ ppm 167.90, 139.26, 135.12, 133.93, 132.04, 127.76, 123.29, 83.73, 41.60, 24.77; ^11^B-NMR (CDCl_3_) δ ppm 30.94.


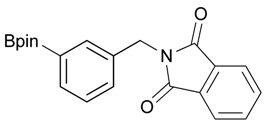


*2-[3-(4,4,5,5-Tetramethyl-1,3,2-dioxaborolan-2-yl)benzyl]isoindoline-1,3-dione* (**3b**). Following the General Procedure B, the desired compound was synthesized utilizing **2b** (767 mg, 3.28 mmol), methanesulfonyl chloride (0.38 mL, 4.92 mmol), DIPEA (1.14 mL, 6.56 mmol), potassium phthalimide salt (911 mg, 4.92 mmol), and potassium carbonate (1,360 mg, 9.84 mmol) giving compound **3b** as a white solid (m.p. 170–173 °C) in 80% yield (953 mg); ^1^H-NMR (CDCl_3_) δ ppm 7.76–7.83 (m, 3H), 7.72–7.68 (m, 3H), 7.51 (d, *J* = 7.70 Hz, 1H), 7.32 (t, *J* = 7.5 Hz, 1H), 4.85 (s, 2H), 1.33 (s, 12H); ^13^C-NMR (CDCl_3_) δ ppm 135.60, 134.78, 134.25, 133.91, 132.13, 131.36, 128.05, 123.31, 83.82, 41.59, 24.84; ^11^B-NMR (CDCl_3_) δ ppm 30.88.


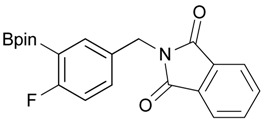


*2-[4-Fluoro-3-(4,4,5,5-tetramethyl-1,3,2-dioxaborolan-2-yl)benzyl]isoindoline-1,3-dione* (**3c**). Following the General Procedure B, the desired compound was synthesized utilizing **2c** (378 mg, 1.50 mmol), methanesulfonyl chloroide (0.17 mL, 2.25 mmol), DIPEA (0.52 mL, 3.00 mmol), potassium phthalimide salt (417 mg, 2.25 mmol), and potassium carbonate (622 mg, 4.5 mmol) giving compound **3c** as a white solid (m.p. 55–58 °C) in 75% yield (429 mg); ^1^H-NMR (CDCl_3_) δ ppm 7.84–7.78 (m, 3H), 7.78–7.67 (m, 2H), 7.52–7.7.47 (m, 1H), 6.95 (t, *J* = 8.8 Hz, 1H), 4.80 (s, 2H), 1.34 (s, 12H); ^13^C-NMR (CDCl_3_) δ ppm 167.93, 137.12, 137.07, 133.96, 133.73, 133,67, 132.03, 131.63, 123.47, 123.32, 115.61, 115.45, 83.95, 40.80, 24.75; ^11^B-NMR (CDCl_3_) δ ppm 30.19.


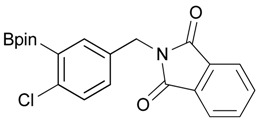


*2-[4-Chloro-3-(4,4,5,5-tetramethyl-1,3,2-dioxaborolan-2-yl)benzyl]isoindoline-1,3-dione* (**3d**). Following the General Procedure B, the desired compound was synthesized utilizing **2d** (348 mg, 1.30 mmol), methanesulfonyl chloroide (0.15 mL, 1.95 mmol), DIPEA (0.45 mL, 2.60 mmol), potassium phthalimide salt (360 mg, 1.95 mmol), and potassium carbonate (539 mg, 3.90 mmol). Compound **3d** was obtained as a white solid (m.p. 102–104 °C) in 85% yield (439 mg); ^1^H-NMR (CDCl_3_) δ ppm 7.78–7.74 (m, 2H), 7.71 (d, *J* = 2.0 Hz, 1H), 7.66–7.62 (m, 2H), 7.35 (q, *J*^1^ = 6.1 Hz, *J*^2^ = 2.1 Hz, 1H), 7.23 (d, *J* = 8.3 Hz, 1H), 4.75 (s, 2H), 1.32 (s, 12H); ^13^C-NMR (CDCl_3_) δ ppm 167.8, 139.0, 136.5, 134.1, 134.0, 132.1, 131.9, 129.6, 123.3, 84.2, 40.9, 24.8; ^11^B-NMR (CDCl_3_) δ ppm 30.6.


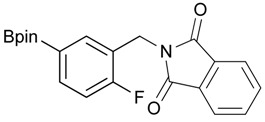


*2-[2-Fluoro-5-(4,4,5,5-tetramethyl-1,3,2-dioxaborolan-2-yl)benzyl]isoindoline-1,3-dione* (**3e**). Following the General Procedure B, the desired compound was synthesized utilizing **2e** (272 mg, 1.08 mmol), methanesulfonyl chloroide (0.13 mL, 1.62 mmol), DIPEA (0.38 mL, 2.16 mmol), potassium phthalimide salt(300 mg, 1.62 mmol), and potassium carbonate (447 mg, 3.24 mmol) giving **3e** as a white solid (m.p. 139–142 °C) in 53% yield (218 mg); ^1^H-NMR (CDCl_3_) δ ppm 7.85–7.79 (m, 3H), 7.73–768 (m, 3H), 7.02 (q, *J*^1^ = 1.8 Hz, *J*^2^ = 8.2 Hz, 1H), 4.91 (s, 2H), 1.32 (s, 12H); ^13^C-NMR (CDCl_3_) δ ppm 167.8, 163.0 (d, *J* = 252.0 Hz), 137.2, 136.7, 136.6, 133.9, 132.1, 123.4, 122.5, 122.4, 115.1, 114.9, 83.9, 35.6, 35.5, 24.8; ^11^B-NMR (CDCl_3_) δ ppm 30.6.


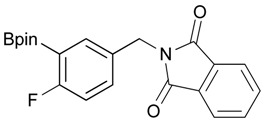


*2-[3-Fluoro-4-(4,4,5,5-tetramethyl-1,3,2-dioxaborolan-2-yl)benzyl]isoindoline-1,3-dione* (**3f**). Following the General Procedure B, the desired compound was synthesized utilizing **2f** (293 mg, 1.16 mmol), methanesulfonyl chloroide (0.14 mL, 1.74 mmol), DIPEA (0.39 mL, 2.32 mmol), potassium phthalimide salt (322 mg, 1.74 mmol), and potassium carbonate (480 mg, 3.48 mmol) giving compound **3f** as a white solid (m.p. 101–103 °C) in 97% yield (429 mg); ^1^H-NMR (CDCl_3_) δ ppm 7.85–7.81 (m, 2H), 7.73–7.66 (m, 3H), 7.17 (d, *J* = 7.7 Hz, 1H), 7.06 (d, *J* = 9.9 Hz, 1H), 4.82 (s, 2H), 1.32 (s, 12H); ^13^C-NMR (CDCl_3_) δ ppm 167.7, 167.2 (d, *J* = 252.0 Hz), 142.0, 141.9, 137.2, 137.2, 134.1, 131.9, 123.5, 123.4, 115.2, 114.9, 83.8, 40.9, 24.7; ^11^B-NMR (CDCl_3_) δ ppm 30.06.


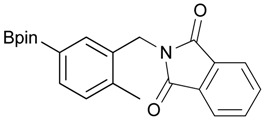


*2-[2-Methyl-5-(4,4,5,5-tetramethyl-1,3,2-dioxaborolan-2-yl)benzyl]isoindoline-1,3-*dione (**3g**). Following the **General Procedure B**, the desired compound was synthesized utilizing **2g** (598 mg, 2.41 mmol), methanesulfonyl chloroide (0.28 mL, 3.62 mmol), DIPEA (0.83 mL, 4.83 mmol), potassium phthalimide salt (670 mg, 3.62 mmol), and potassium carbonate (1,000 mg, 7.24 mmol) giving compound **3g** as a white solid (m.p. 168–171 °C) in 48% yield (438 mg); ^1^H-NMR (CDCl_3_) δ ppm 7.81–7.76 (m, 3H), 7.68–7.61 (m, 3H), 7.16 (d, *J* = 7.4 Hz, 1H), 4.86 (s, 2H), 2.49 (s, 3H), 1.29 (s, 12H); ^13^C-NMR (CDCl_3_) δ ppm 167.8, 139.5, 134.9, 134.2, 133.7, 133.4, 131.8, 129.7, 126.5, 122.9, 83.4, 39.1, 24.6, 19.6; ^11^B-NMR (CDCl_3_) δ ppm 31.1.

### 3.4. General Procedure C for the Synthesis of Boron-Containing Primary Amines ***4a–g***


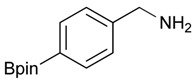


*[4-(4,4,5,5-Tetramethyl-1,3,2-dioxaborolan-2-yl)phenyl]methanamine* (**4a**). Compound **3a** (196 mg, 0.54 mmol) was added to a dry flask containing magnetic stir and dissolved in THF (5.50 mL). Afterwards, hydrazine hydrate (0.08 mL, 1.62 mmol) was added to the reaction mixture. The reaction was stirred under a reflux for 12 h. The resulting dried crude material was then re-suspended with chloroform (50.00 mL), filtered, and the filtrate was concentrated *in vacuo*. The crude material was purified by High Performance Liquid Chromatography (HPLC) to give the desired product **4a** as a white solid (m.p. 85 °C) in 87% yield (111 mg); ^1^H-NMR (CDCl_3_) δ ppm 7.78 (d, *J* = 7.9 Hz, 2H), 7.32 (d, *J* = 8.0 Hz, 2H), 3.88 (s, 2H), 1.34 (s, 12H); ^13^C-NMR (CDCl_3_) δ ppm 136.72, 135.56, 127.18, 84.11, 43.94, 25.02; ^11^B-NMR (CD_3_OD-*d_4_*) δ ppm 30.90; HRMS (ESI, positive ion) (*m/z*): [M+H]^+^ calcd for C_13_H_20_BNO_2_, 234.1669; found, 234.1651.


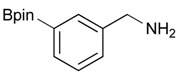


*[3-(4,4,5,5-Tetramethyl-1,3,2-dioxaborolan-2-yl)phenyl]methanamine* (**4b**). Following the General Procedure C, the desired compound was synthesized utilizing **3b** (250 mg, 0.69 mmol), THF (7.00 mL), and hydrazine hydrate (0.10 mL, 2.07 mmol) giving compound **4b** as a white solid (m.p. 91 °C) in 82% yield (131 mg); ^1^H-NMR (CDCl_3_) δ ppm 7.74–7.68 (m, 2H), 7.42–7.32 (m, 2H), 3.86 (s, 2H), 1.34 (s, 12H); ^13^C-NMR (CDCl_3_) δ ppm 142.78, 133.58, 133.48, 130.30, 128.23, 84.03, 46.69, 25.07; ^11^B-NMR (CD_3_OD-*d_4_*) δ ppm 30.94; HRMS (ESI, positive ion) (*m/z*): [M+H]^+^ calcd for C_13_H_20_BNO_2_, 234.1669; found, 234.1644.


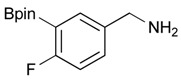


*[4-Fluoro-3-(4,4,5,5-tetramethyl-1,3,2-dioxaborolan-2-yl)phenyl]methanamine* (**4c**). Following the General Procedure C, the desired compound was synthesized utilizing **3c** (953 mg, 2.50 mmol), THF (25.00 mL), and hydrazine hydrate(0.36 mL, 7.50 mmol) giving compound **4c** as a white solid (m.p. 62 °C) with 98% yield (616 mg); ^1^H-NMR (CDCl_3_) δ ppm 7.66–7.63 (m, 1H), 7.40–7.34 (m, 1H), 6.99 (t, *J* = 5.9 Hz, 1H), 3.83 (s, 2H), 1.34 (s, 12H); ^13^C-NMR (CDCl_3_) δ ppm 166.44 (d, *J* = 249.9 Hz), 138.64, 135.58, 135.47, 132.37, 132.25, 116.48, 116.31, 84.13, 45.92, 25.03; ^11^B-NMR (CD_3_OD-*d_4_*) δ ppm 30.12; HRMS (ESI, positive ion) (*m/z*): [M+H]^+^ calcd for C_13_H_19_BFNO_2_, 252.1574; found, 252.1548.


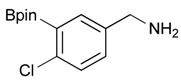


*[4-Chloro-3-(4,4,5,5-tetramethyl-1,3,2-dioxaborolan-2-yl)phenyl]methanamine* (**4d**). Following the General Procedure C, the desired compound was synthesized utilizing **3d** (524 mg, 1.32 mmol), THF (13.00 mL), and hydrazine hydrate (0.20 mL, 3.96 mmol) giving compound **4d** as a white solid (m.p. 56 °C) in 36% yield (128 mg); ^1^H-NMR (CDCl_3_) δ ppm 7.59 (s, 1H), 7.31–7.26 (m, 2H), 4.19 (s, 2H), 1.27 (s, 12H); ^13^C-NMR (CDCl_3_) δ ppm 135.26, 134.97, 131.96, 130.91, 130.59, 84.37, 45.76, 24.95; ^11^B-NMR (CD_3_OD-*d_4_*) δ ppm 30.45; HRMS (ESI, positive ion) (*m/z*): [M+H]^+^ calcd for C_13_H_19_BClNO_2_, 268.1279; found, 268.1153.


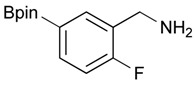


*[2-Fluoro-5-(4,4,5,5-tetramethyl-1,3,2-dioxaborolan-2-yl)phenyl]methanamine* (**4e**). Following the General Procedure C, the desired compound was synthesized utilizing **3e** (679 mg, 1.78 mmol), THF (18.00 mL), and hydrazine hydrate (0.26 mL, 5.34 mmol) giving compound **4e** as a white solid (m.p. 78 °C) in 91% yield (405 mg); ^1^H-NMR (CDCl_3_) δ ppm 7.76–7.66 (m, 2H), 7.02 (t, 1H), 3.91 (s, 2H), 1.33 (s, 12H); ^13^C-NMR (CDCl_3_) δ ppm 163.27 (d, *J* = 126.8 Hz), 138.35, 136.29, 136.24, 124.02, 123.93, 83.96, 36.59, 24.88; ^11^B-NMR (CD_3_OD-*d_4_*) δ ppm 30.05; HRMS (ESI, positive ion) (*m/z*): [M+H]^+^ calcd for C_13_H_19_BFNO_2_, 252.1574; found, 252.1549.


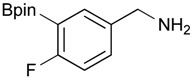


*[3-Fluoro-4-(4,4,5,5-tetramethyl-1,3,2-dioxaborolan-2-yl)phenyl]methanamine* (**4f**). Following the General Procedure C, the desired compound was synthesized utilizing **3f** (907 mg, 2.38 mmol), THF (24.00 mL), and hydrazine hydrate (0.34 mL, 7.15 mmol) giving compound **4f** as a white solid (m.p. 52 °C) in 66% yield (396 mg); ^1^H-NMR (CDCl_3_) δ ppm 7.70 (t, *J* = 6.5 Hz, 1H), 7.10 (d, *J* = 7.8 Hz, 1H), 7.04 (d, *J* = 9.7 Hz, 1H), 3.93 (s, 2H), 1.30 (s, 12H); ^13^C-NMR (CDCl_3_) δ ppm 167.24 (d, *J* = 252.2 Hz), 137.98, 137.87, 124.21, 116.05, 115.72, 84.37, 43.20, 25.02, 24.93; ^11^B-NMR (CDCl_3_-*d_3_*) δ ppm 30.16; HRMS (ESI, positive ion) (*m/z*): [M+H]^+^ calcd for C_13_H_19_BFNO_2_, 252.1574; found, 252.1549.


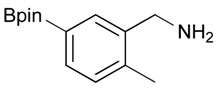


*[2-Methyl-5-(4,4,5,5-tetramethyl-1,3,2-dioxaborolan-2-yl)phenyl]methanamine* (**4g**). Following the General Procedure C, the desired compound was synthesized utilizing **3g** (690 mg, 1.83 mmol), THF (18.00 mL), and hydrazine hydrate (0.26 mL, 5.49 mmol) giving compound **4g** as a white solid (m.p. 59 °C) in 58% yield (262 mg); ^1^H-NMR (CDCl_3_) δ ppm 7.62–7.60 (m, 2H), 7.17 (d, *J* = 6.7 Hz, 1H), 3.75 (s, 2H), 2.38 (s, 3 H), 1.34 (s, 12H); ^13^C-NMR (CDCl_3_) δ ppm 140.44, 136.90, 134.49, 134.32, 130.10, 83.71, 42.04, 24.91, 19.68; ^11^B-NMR (CDCl_3_) δ ppm 31.01; HRMS (ESI, positive ion) (*m/z*): [M+H]^+^ calcd for C_14_H_22_BNO_2_, 248.1489; found, 248.9602.

### 3.5. General Procedure D for the Synthesis of Ugi-4CR Boronate esters ***5a–c***


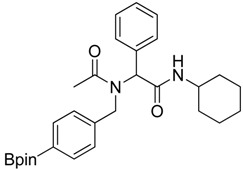


*N-Cyclohexyl-2-phenyl-2-{N-[4-(4,4,5,5-tetramethyl-1,3,2-dioxaborolan-2-yl)benzyl] acetamido}acetamide* (**5a**). A 10 mL glass tube containing **4a** (396 mg, 1.70 mmol), benzylaldehyde (0.17 mL, 1.70 mmol) and MeOH (3.40 mL) was first microwave irradiated for 120 min (50 °C, 150 W) under medium speed magnetic stirring. Acetic acid (0.12 mL, 2.04 mmol) and cyclohexyl isocyanide (0.21 mL, 1.70 mmol) were then added to the reaction mixture. Additional microwave irradiation was applied for 120 min (50 °C, 150 W) under moderate magnetic stirring. After the reaction was completed, MeOH was removed *in vacuo*. The crude material was re-dissolved in ethyl acetate and the resulting organic solution was washed with 1 M aqueous HCl solution. This was followed by adding a saturated aqueous solution of K_2_CO_3_ combined with brine. The resulting organic layer was collected, dried by MgSO_4_, and then concentrated *in vacuo.* Afterwards*, * the crude material was purified by flash column chromatography on silica gel using *n*-hexane/ethyl acetate = 1:1 as the eluent to afford the desired product 5a as a white solid (m.p. 100 °C) in 69% yield (575 mg); ^1^H-NMR (CDCl_3_) δ ppm 7.59 (d, *J* = 7.7 Hz, 2H), 7.44–7.18 (m, 5H), 7.10–6.90 (m, 2H), 6.08 (s, 0.55H), 5.79 (b, 0.45H), 4.66 (q, *J*^1^ = 41.9, *J*^2^ = 18.1, 2H), 3.77 (br, 1H), 2.00–1.03 (m, 22H); ^13^C-NMR (CDCl_3_) δ ppm 172.9, 168.8, 141.1, 135.4, 135.0, 129.8, 128.9, 128.7, 128.6, 127.5, 125.5, 83.9, 62.3, 50.8, 48.7, 32.9, 26.1, 25.6, 25.0, 24.9, 24.9, 22.6; ^11^B-NMR (CD_3_OD-*d_4_*) δ ppm 31.2; HRMS (ESI, positive ion) (*m/z*): [M+H]^+^ calcd for C_29_H_39_BN_2_O_4_, 491.3079; found, 491.3067.


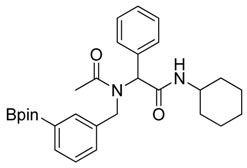


*N-Cyclohexyl-2-phenyl-2-{N-[3-(4,4,5,5-tetramethyl-1,3,2-dioxaborolan-2-yl)benzyl]acetamido}acetamide* (**5b**). Following the General Procedure D, the desired compound was synthesized utilizing **4b** (394 mg, 1.69 mmol), benzylaldehyde (0.17 mL, 1.69 mmol), cyclohexyl isocyanide (0.21 mL, 1.69 mmol), acetic acid (0.12 mL, 2.03 mmol), and MeOH (3.40 mL) giving compound **5b** as a white solid (m.p. 91 °C) in 52% yield (430 mg); ^1^H-NMR (Bruker AC-600 FT-NMR spectrometer at 600 MHz, CDCl_3_) δ ppm 7.57 (d, *J* = 7.2 Hz, 1H), 7.33–7.09 (m, 8 H), 5.92 (s, 1H), 5.67 (br, 0.45H), 4.65 (q, *J*^1^ = 46.5, *J*^2^ = 17.4, 2H), 3.75 (br, 1H), 2.10 (s, 3H), 1.64–1.05 (m, 22H); ^13^C-NMR (Bruker AC-600 FT-NMR spectrometer at 150.9 MHz, CDCl_3_) δ ppm 22.3, 24.8, 25.2, 32.6, 48.7, 50.9, 63.0, 83.8, 127.8, 128.7, 128.9, 129.0, 129.5, 132.4, 133.4, 134.8, 136.3, 168.7, 173.3; ^11^B-NMR (CD_3_OD-*d_4_*) δ ppm 31.08; HRMS (ESI, positive ion) (*m/z*): [M+H]^+^ calcd for C_29_H_39_BN_2_O_4_, 491.3079; found, 491.3057.


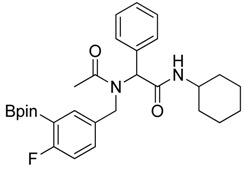


*N-Cyclohexyl-2-{N-[4-fluoro-3-(4,4,5,5-tetramethyl-1,3,2-dioxaborolan-2-yl)benzyl]acetamido}-2-phenylacetamide* (**5c**). Following the General Procedure D, the desired compound was synthesized utilizing **4c** (733 mg, 2.92 mmol), benzylaldehyde (0.30 mL, 2.92 mmol), cyclohexyl isocyanide (0.36 mL, 2.92 mmol), acetic acid (0.20 mL, 3.50 mmol), and MeOH (4.00 mL) giving **5c** a white solid (m.p. 110 °C) in 74% yield (1,100 mg); ^1^H-NMR (CDCl_3_) δ ppm 7.32–7.06 (m, 7H), 6.81(br, 1H), 6.00 (s, 0.50H), 5.66 (br, 0.50H), 4.62 (q, *J*^1^ = 44.0, *J*^2^ = 17.3, 2H), 3.76 (m, 1H), 2.06 (s, 3H), 1.85–1.06 (m, 22H); ^13^C-NMR (CDCl_3_) δ ppm 172.62, 168.62, 166.97, 165.31, 135.11, 134.32, 134.28, 132.68, 130.92, 130.87, 129.73, 128.76, 128.55, 115.15, 83.91, 62.39, 49.99, 48.61, 31.91, 29.34, 24.82, 24.58, 22.47; ^11^B-NMR (CDCl_3_) δ ppm 29.94; HRMS (ESI, positive ion) (*m/z*): [M+H]^+^ calcd for C_30_H_40_BFN_2_O_4_, 509.2985; found, 509.3139.


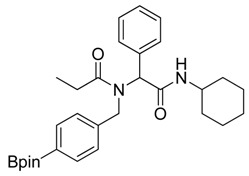


*N-Cyclohexyl-2-phenyl-2-{N-[4-(4,4,5,5-tetramethyl-1,3,2-dioxaborolan-2-yl)benzyl]acetamido}propionamide* (**5d**). Following the General Procedure D, the desired compound was synthesized utilizing **4a** (487 mg, 2.09 mmol), benzylaldehyde (0.21 mL, 2.09 mmol), cyclohexyl isocyanide (0.26 mL, 2.09 mmol), propionic acid (0.19 mL, 2.50 mmol), and MeOH (4.00 mL) giving **5d** as a white solid (m.p. 102 °C) in 44% yield (464 mg); ^1^H-NMR (CDCl_3_) δ ppm 7.59 (d, *J* = 7.3 Hz, 2H), 7.34–7.23 (m, 5H), 6.93 (d, *J* = 7.2, 2H), 6.09 (s, 0.48H), 5.66 (b, 0.52H), 4.66 (q, *J*^1^ = 46.4, *J*^2^ = 18.1, 2H), 3.78 (br, 1H), 2.37–1.04 (m, 27H); ^13^C-NMR (CDCl_3_) δ ppm 175.9, 168.8, 141.1, 135.2, 134.7, 129.7, 128.7, 128.5, 125.2, 83.7, 62.4, 49.9, 48.5, 32.7, 26.2, 25.4, 24.8, 24.7, 9.2; ^11^B-NMR (CDCl_3_) δ ppm 30.6; HRMS (ESI, positive ion) (*m/z*): [M+H]^+^ calcd for C_30_H_41_BN_2_O_4_, 505.3157; found, 505.3219.

### 3.6. General Procedure E for the Synthesis of Ugi-4CR Boronic Acid Derivatives ***6a–c***


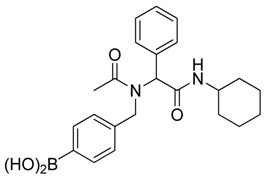


*[4-({N-[2-(Cyclohexylamino)-2-oxo-1-phenylethyl]acetamido}methyl)phenyl]boronic* acid (**6a**). Compound **5a** (917 mg, 1.87 mmol) and KHF_2_ (876 mg, 11.22 mmol) were added to a flask containing magnetic stir, which was then suspended in MeOH/H_2_O (1:1, v/v, 9.35 mL). The reaction mixture was stirred vigorously at room temperature for 6 hours, and the solvents were removed *in vacuo*. The resulting mixture of solids were dried under a dry-freezing vacuum overnight and then subjected to extraction with hot acetone (100.00 mL). The resulting acetone extracts were concentrated i*n vacuo.* SiO_2_ (123 mg, 2.05 mmol) was added to this crude material which was re-suspended in H_2_O/ethyl acetate (1:1, v/v) (5.5 mL). The reaction was stirred at room temperature until ^11^B-NMR indicated completion of the reaction (5 h). The reaction mixture was filtered to remove SiO_2_, and the filter cake was thoroughly rinsed with ethyl acetate. The aqueous layer was separated from the organic layer, and the aqueous layer was extracted with ethyl acetate (2 × 15 mL). The organic layer was collected and dried with MgSO_4_. This organic solution was then filtered, and concentrated *in vacuo* to afford the desired pure product **6a** as a white solid (m.p. 177 °C) in 49% yield (374 mg); ^1^H-NMR (CD_3_OD-*d_4_*) δ ppm 7.56–7.20 (m, 6H), 6.94–6.89 (m, 3H), 6.09 (s, 0.81H), 5.67 (br, 0.23H), 4.64 (q, *J*^1^ = 88.5, *J*^2^ = 19.4, 2H), 3.69–3.54 (br, 1H), 2.08 (s., 3H), 1.96–1.06 (m, 10H); ^13^C-NMR (CD_3_OD-*d_4_*) δ ppm 175.4, 171.6, 136.4, 135.1, 134.7, 131.2, 130.2, 129.8, 129.7, 127.6, 126.3, 63.9, 51.3, 50.2, 33.6, 33.6, 26.7, 26.2, 26.2, 22.7; ^11^B-NMR (CD_3_OD-*d_4_*) δ ppm 28.09; HRMS (ESI, positive ion) (*m/z*): [M+H]^+^ calcd for C_23_H_29_BN_2_O_4_, 409.2297; found, 409.2310.


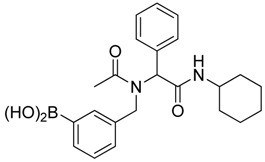


*[3-({N-[2-(Cyclohexylamino)-2-oxo-1-phenylethyl]acetamido}methyl)phenyl]boronic acid* (**6b**). Following the General Procedure E, the desired compound was synthesized utilizing **5b** (431 mg, 0.88 mmol), KHF_2_ (412 mg, 5.28 mmol), SiO_2_ (58 mg, 0.96 mmol), and MeOH/H_2_O (1:1, v/v, 4.5 mL) giving **6b** as a white solid (m.p. 150 °C) in 60% yield (215 mg); ^1^H-NMR (CD_3_OD-*d_4_*) δ ppm 7.51–6.98 (m, 9H), 6.10 (s, 0.81H), 5.66 (br, 0.19H), 4.69 (q, *J*^1^ = 70.7, *J*^2^ = 18.1, 2H), 3.66 (br, 1H), 2.09 (s, 3H), 1.82–1.03 (m, 10H); ^13^C-NMR (CD_3_OD-*d_4_*) δ ppm 175.25, 171.43, 170.26, 138.30, 138.05, 136.65, 136.35, 133.93, 133.37, 133.08, 132.87, 132.46, 131.90, 131.01, 130.36, 130.08, 129.95, 129.65, 129.48, 128.85, 128.61, 128.46, 128.31, 66.41, 63.76, 51.21, 50.11, 33.46, 26.60, 26.09, 26.04, 22.56; ^11^B-NMR (CD_3_OD-*d_4_*) δ ppm 28.58; HRMS (ESI, positive ion) (*m/z*): [M+H]^+^ calcd for C_23_H_29_BN_2_O_4_, 409.2318; found, 409.2297.


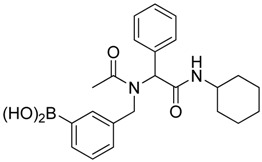


*[5-({N-[2-(Cyclohexylamino)-2-oxo-1-phenylethyl]acetamido}methyl)-2-fluorophenyl]boronic Acid* (**6c**). Following the General Procedure E, the desired compound was synthesized utilizing **5c** (46 mg, 0.09 mmol), KHF_2_ (42 mg; 0.54 mmol), SiO_2_ (6 mg; 0.10 mmol), and MeOH/H_2_O (1:1, v/v, 2.0 mL) giving compound **6c** as a white solid (m.p. 61 °C) with 10% yield (4 mg); ^1^H-NMR (Bruker AC-600 FT-NMR spectrometer at 600 MHz, CD_3_OD-*d_4_*) δ ppm 7.27–7.20 (m, 5H), 6.97–6.82 (m, 3H), 6.10 (s, 0.72H), 5.66 (br, 0.28H), 4.65 (q, *J*^1^ = 127.8, *J*^2^ = 17.4, 2H), 3.65 (br, 1H), 2.19 (s, 0.89H), 2.08 (s, 2.11H), 1.85–1.59 (m, 5H), 1.34–1.14 (m, 5H); ^13^C-NMR (CD_3_OD-*d_4_*) δ ppm 175.21, 174.62, 171.36, 170.35, 136.51, 136.34, 135.62, 134.81, 134.08, 132.57, 131.10, 129.68, 115.57, 115.41, 114.83, 66.27, 63.48, 50.51, 50.02, 33.51, 33.46, 26.60, 26.10, 26.05, 22.49; ^11^B-NMR (CD_3_OD-*d_4_*) δ ppm 28.46; HRMS (ESI, positive ion) (*m/z*): [M+H]^+^ calcd for C_23_H_28_BFN_2_O_4_, 427.2202; found, 427.2204

### 3.7. General Procedure F for the Synthesis ***8a–b***


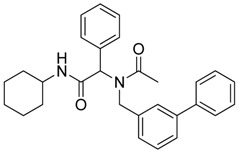


*2-[N-([1,1'-Biphenyl]-3-ylmethyl)acetamido]-N-cyclohexyl-2-phenylacetamid* (**8a**). Compound **5b** (284 mg, 0.58 mmol), bromobenzene (0.08 mL, 0.75 mmol), and Pd(PPh_3_)_2_Cl_2_ (40 mg, 0.06 mmol) were added to a flask containing a magnetic stir with EtOH (5.80 mL). Triethylamine (0.18 mL, 1.28 mmol) was then added to the reaction mixture and the reaction mixture was stirred under reflux condition for 10 h under a N_2_ atmosphere. After cooling the mixture to room temperature, solvent was removed *in vacuo*. The crude material was diluted with ethyl acetate (5.00 mL) and water (5.00 mL). The aqueous layer was separated from the organic layer, and the aqueous layer was extracted with ethyl acetate (2 × 15 mL). Organic layers were then combined, washed with brine solution, and dried with MgSO_4_, and solvent was removed *in vacuo*. The crude material was then purified by flash column chromatography on silica gel using *n*-hexane/ethyl acetate = 1:1 as the eluent to give the desired product **8a** as a white solid (m.p. 180 °C) in 57% yield (146 mg); white solid; ^1^H-NMR (Bruker AC-600 FT-NMR spectrometer at 600 MHz, CD_3_OD-*d_4_*) δ ppm 7.47–7.19 (m, 12H), 7.07 (s, 1H), 6.96 (d, *J* = 7.8 Hz, 1H), 6.15 (s, 0.77H), 5.68 (br, 0.23H), 4.75 (q, *J*^1^ = 115.8, *J*^2^ = 18.0, 2H), 3.65 (m, 1H), 2.11 (s, 3H), 1.82–1.08 (m, 10H); ^13^C-NMR (Bruker AC-600 FT-NMR spectrometer at 150.9 MHz, CD_3_OD-*d_4_*) δ ppm 175.41, 171.51, 142.75, 142.33, 140.03, 136.65, 131.20, 130.26, 130.00, 129.93, 129.72, 128.56, 128.15, 126.67, 126.21, 125.77, 63.73, 51.35, 50.19, 33.63. 26.75, 26.25, 26.20, 22.72; HRMS (ESI, positive ion) (*m/z*): [M+H]^+^ calcd for C_29_H_32_N_2_O_2_, 441.2543; found, 441.2542.


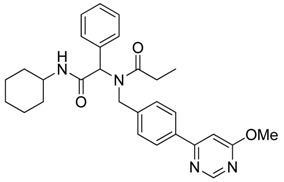


*N-[2-(Cyclohexylamino)-2-oxo-1-phenylethyl]-N-[4-(6-methoxypyrimidin-4-yl)benzyl]propionamide* (**8b**). Following the General Procedure F, the desired compound was synthesized, utilizing *N*-[2-(cyclohexylamino)-2-oxo-1-phenylethyl]-*N*-[4-(4,4,5,5-tetramethyl-1,3,2-dioxaborolan-2-yl)benzyl]-propionamide (**5d**, 236 mg, 0.52 mmol), 4-chloro-6-methoxypyrimidine (105.24 mg, 0.728 mmol), Pd(PPh_3_)_2_Cl_2_ (15 mg, 0.06 mmol), and triethylamine (0.17 mL, 1.23 mmol) giving compound **8b** as a white solid (m.p. 174 °C) in 82% yield (223 mg); ^1^H-NMR (CDCl_3_) δ ppm 8.76 (s, 1 H), 7.78 (d, *J* = 8.2 Hz, 1H), 7.32–6.66 (m, 9H), 6.11 (s, 0.42H), 5.93 (b, 0.58H), 4.68 (q, *J*^1^ = 51.4, *J*^2^ = 18.2, 2H), 3.97 (s, 3H), 3.77 (m, 1H), 2.40–2.13 (m, 2H), 1.84–1.02 (m, 13H); ^13^C-NMR (CDCl_3_) δ ppm 176.29, 175.82, 169.97, 164.83, 158.44, 141.87, 135.43, 129.84, 128.87, 128.79, 128.71, 127.10, 126.57, 117.01, 103.40, 62.73, 54.01, 49.91, 48.76, 32.85, 27.41, 25.56, 24.91, 24.83, 9.46; HRMS (ESI, positive ion) (*m/z*): [M+H]^+^ calcd for C_29_H_34_N_4_O_3_, 487.2710; found, 487.2702.

## 4. Conclusions

In summary, seven boron-containing primary amines were successfully synthesized from the corresponding formylphenyl boronate esters. Further, the synthesized amines were incorporated into the peptoid-like backbone in moderate to good yields via an Ugi-4CR reaction under microwave-assisted conditions. The boronate ester group of the peptoid-like analogs was successfully transformed to provide the corresponding boronic acids using a simple two-step protocol. In addition, the structure of the peptoid-like boronate esters was further modified by coupling to aryl/heteroaryl chlorides via palladium-mediated Suzuki cross-coupling reactions. These results suggest that boron-containing primary amines could be a unique building block for use in the progression of the field of peptoid research.

## Supplementary Materials

Supplementary materials can be accessed at: http://www.mdpi.com/1420-3049/18/10/12346/s1.
