# Supplementary material for: Synthesis of Boron-Containing Primary Amines
Source: Molecules. 2013 Oct 8;18(10):12346–67. doi: 10.3390/molecules181012346 (PMC6269827; doi:10.3390/molecules181012346)
Supplement: Supplementary file 1 [file molecules-18-12346-s001.pdf]

# Supplementary Materials

## Contents

300 MHz $^{1}$H-NMR of Compound (**2a**) in $CDCl_3$ ........ **S4**
150.9 MHz $^{13}$C-NMR of Compound (**2a**) in $CDCl_3$ ........ **S4**
192.5 MHz $^{11}$B-NMR of Compound (**2a**) in $CDCl_3$ ........ **S5**
300 MHz $^{1}$H-NMR of Compound (**2b**) in $CDCl_3$ ........ **S5**
150.9 MHz $^{13}$C-NMR of Compound (**2b**) in $CDCl_3$ ........ **S6**
192.5 MHz $^{11}$B-NMR of Compound (**2b**) in $CDCl_3$ ........ **S6**
300 MHz $^{1}$H-NMR of Compound (**2c**) in $CD_3OD$-*d₄* ........ **S7**
150.9 MHz $^{13}$C-NMR of Compound (**2c**) in $CD_3OD$-$d_4$ ........ **S7**
192.5 MHz $^{11}$B-NMR of Compound (**2c**) in $CDCl_3$ ........ **S8**
300 MHz $^{1}$H-NMR of Compound (**2d**) in $CD_3OD$-$d_4$ ........ **S8**
75.5 MHz $^{13}$C-NMR of Compound (**2d**) in $CDCl_3$ ........ **S9**
192.5 MHz $^{11}$B-NMR of Compound (**2d**) in $CDCl_3$ ........ **S9**
300 MHz $^{1}$H-NMR of Compound (**2e**) in $CD_3OD$-$d_4$ ........ **S10**
150.9 MHz $^{13}$C-NMR of Compound (**2e**) in $CD_3OD$-$d_4$ ........ **S10**
192.5 MHz $^{11}$B-NMR of Compound (**2e**) in $CDCl_3$ ........ **S11**
300 MHz $^{1}$H-NMR of Compound (**2f**) in $CDCl_3$ ........ **S11**
150.9 MHz $^{13}$C-NMR of Compound (**2f**) in $CDCl_3$ ........ **S12**
192.5 MHz $^{11}$B-NMR of Compound (**2f**) in $CDCl_3$ ........ **S12**
300 MHz $^{1}$H-NMR of Compound (**2g**) in $CD_3OD$-$d_4$ ........ **S13**
150.9 MHz $^{13}$C-NMR of Compound (**2g**) in $CD_3OD$-$d_4$ ........ **S13**
192.5 MHz $^{11}$B-NMR of Compound (**2g**) in $CDCl_3$ ........ **S14**
300 MHz $^{1}$H-NMR of Compound (**3a**) in $CDCl_3$ ........ **S14**
150.9 MHz $^{13}$C-NMR of Compound (**3a**) in $CDCl_3$ ........ **S15**
192.5 MHz $^{11}$B-NMR of Compound (**3a**) in $CDCl_3$ ........ **S15**
300 MHz $^{1}$H-NMR of Compound (**3b**) in $CDCl_3$ ........ **S16**
150.9 MHz $^{13}$C-NMR of Compound (**3b**) in $CDCl_3$ ........ **S16**
192.5 MHz $^{11}$B-NMR of Compound (**3b**) in $CDCl_3$ ........ **S17**
300 MHz $^{1}$H-NMR of Compound (**3c**) in $CDCl_3$ ........ **S17**
150.9 MHz $^{13}$C-NMR of Compound (**3c**) in $CDCl_3$ ........ **S18**
192.5 MHz $^{11}$B-NMR of Compound (**3c**) in $CDCl_3$ ........ **S18**
300 MHz $^{1}$H-NMR of Compound (**3d**) in $CDCl_3$ ........ **S19**
75.5 MHz $^{13}$C-NMR of Compound (**3d**) in $CDCl_3$ ........ **S19**

192.5 MHz $^{11}$B-NMR of Compound (**3d**) in $CDCl_3$ ............ **S20**

300 MHz $^{1}$H-NMR of Compound (**3e**) in $CDCl_3$ ............ **S20**

150.9 MHz $^{13}$C-NMR of Compound (**3e**) in $CDCl_3$ ............ **S21**

192.5 MHz $^{11}$B-NMR of Compound (**3e**) in $CDCl_3$ ............ **S21**

300 MHz $^{1}$H-NMR of Compound (**3f**) in $CDCl_3$ ............ **S22**

75.5 MHz $^{13}$C-NMR of Compound (**3f**) in $CDCl_3$ ............ **S22**

192.5 MHz $^{11}$B-NMR of Compound (**3f**) in $CDCl_3$ ............ **S23**

300 MHz $^{11}$H-NMR of Compound (**3g**) in $CDCl_3$ ............ **S23**

150.9 MHz $^{13}$C-NMR of Compound (**3g**) in $CDCl_3$ ............ **S24**

192.5 MHz $^{11}$B-NMR of Compound (**3g**) in $CDCl_3$ ............ **S24**

300 MHz $^{1}$H-NMR of Compound (**4a**) in $CDCl_3$ ............ **S25**

75.5 MHz $^{13}$C-NMR of Compound (**4a**) in $CDCl_3$ ............ **S25**

192.5 MHz $^{11}$B-NMR of Compound (**4a**) in $CD_3OD$-$d_4$ ............ **S26**

300 MHz $^{1}$H-NMR of Compound (**4b**) in $CDCl_3$ ............ **S26**

75.5 MHz $^{13}$C-NMR of Compound (**4b**) in $CDCl_3$-$d_4$ ............ **S27**

192.5 MHz $^{11}$B-NMR of Compound (**4b**) in $CD_3OD$-$d_4$ ............ **S27**

300 MHz $^{1}$H-NMR of Compound (**4c**) in $CDCl_3$ ............ **S28**

75.5 MHz $^{13}$C-NMR of Compound (**4c**) in $CDCl_3$ ............ **S28**

192.5 MHz $^{11}$B-NMR of Compound (**4c**) in $CD_3OD$-$d_4$ ............ **S29**

300 MHz $^{1}$H-NMR of Compound (**4d**) in $CDCl_3$ ............ **S29**

75.5 MHz $^{13}$C-NMR of Compound (**4d**) in $CDCl_3$ ............ **S30**

192.5 MHz $^{11}$B-NMR of Compound (**4d**) in $CD_3OD$-$d_4$ ............ **S30**

300 MHz $^{1}$H-NMR of Compound (**4e**) in $CDCl_3$ ............ **S31**

75.5 MHz $^{13}$C-NMR of Compound (**4e**) in $CDCl_3$ ............ **S31**

192.5 MHz $^{11}$B-NMR of Compound (**4e**) in $CD_3OD$-$d_4$ ............ **S32**

300 MHz $^{1}$H-NMR of Compound (**4f**) in $CDCl_3$ ............ **S32**

75.5 MHz $^{13}$C-NMR of Compound (**4f**) in $CDCl_3$ ............ **S33**

192.5 MHz $^{11}$B-NMR of Compound (**4f**) in $CDCl_3$ ............ **S33**

300 MHz $^{1}$H-NMR of Compound (**4g**) in $CDCl_3$ ............ **S34**

150.9 MHz $^{13}$C-NMR of Compound (**4g**) in $CDCl_3$ ............ **S34**

192.5 MHz $^{11}$B-NMR of Compound (**4g**) in $CDCl_3$ ............ **S35**

600 MHz $^{1}$H-NMR of Compound (**5a**) in $CDCl_3$-$d_3$ ............ **S35**

150.9 MHz $^{13}$C-NMR of Compound (**5a**) in $CDCl_3$ ............ **S36**

192.5 MHz $^{11}$B-NMR of Compound (**5a**) in $CD_3OD$-$d_4$ ............ **S36**

600 MHz $^{1}$H-NMR of Compound (**5b**) in $CDCl_3$ ........................................ **S37**
150.9 MHz $^{13}$C-NMR of Compound (**5b**) in $CDCl_3$ ........................................ **S37**
192.5 MHz $^{11}$B-NMR of Compound (**5b**) in $CD_3OD$-$d_4$........................................ **S38**
300 MHz $^{1}$H-NMR of Compound (**5c**) in $CDCl_3$........................................ **S38**
150.9 MHz $^{13}$C-NMR of Compound (**5c**) in $CDCl_3$........................................ **S39**
192.5 MHz $^{11}$B-NMR of Compound (**5c**) in $CDCl_3$........................................ **S39**
300 MHz $^{1}$H-NMR of Compound (**6a**) in $CD_3OD$-$d_4$........................................ **S40**
75.5 MHz $^{13}$C-NMR of Compound (**6a**) in $CD_3OD$-$d_4$........................................ **S40**
192.5MHz $^{11}$B-NMR of Compound (**6a**) in $CD_3OD$-$d_4$........................................ **S41**
300 MHz $^{1}$H-NMR of Compound (**6b**) in $CD_3OD$-$d_4$........................................ **S41**
150.9 MHz $^{13}$C-NMR of Compound (**6b**) in $CD_3OD$-$d_4$........................................ **S42**
192.5 MHz $^{11}$B-NMR of Compound (**6b**) in $CD_3OD$-$d_4$........................................ **S42**
600 MHz $^{1}$H-NMR of Compound (**6c**) in $CD_3OD$-$d_4$ ........................................ **S43**
75.5 MHz $^{13}$C-NMR of Compound (**6c**) in $CD_3OD$-$d_4$ ........................................ **S43**
192.5 MHz $^{11}$B-NMR of Compound (**6c**) in $CD_3OD$-$d_4$ ........................................ **S44**
600 MHz $^{1}$H-NMR of Compound (**8a**) in $CD_3OD$-$d_4$........................................ **S44**
150.9 MHz $^{13}$C-NMR of Compound (**8a**) in $CD_3OD$-$d_4$........................................ **S45**
300 MHz $^{1}$H-NMR of Compound (**8b**) in $CDCl_3$ ........................................ **S45**
75.5 MHz $^{13}$C-NMR of Compound (**8b**) in $CDCl_3$ ........................................ **S46**

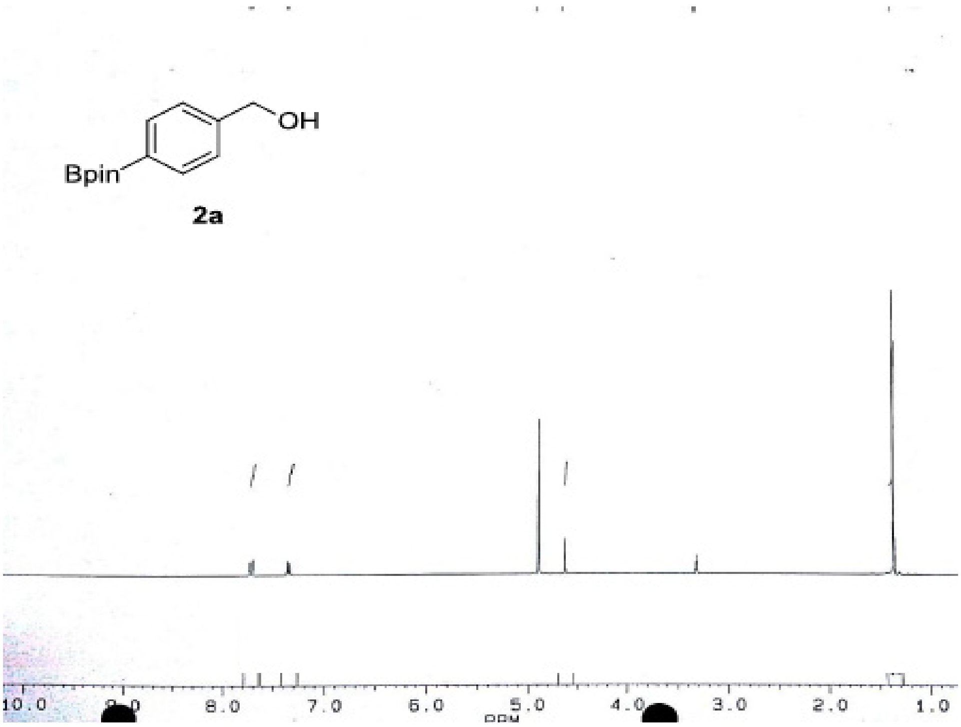


300 MHz $^{1}$H-NMR of Compound (2a) in $CDCl_3$

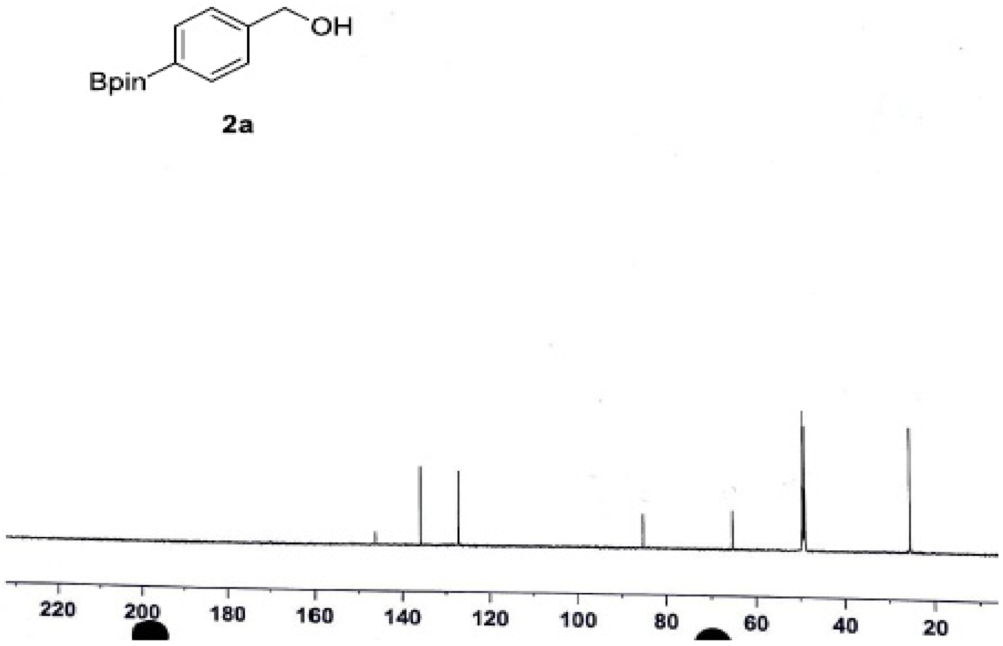


150.9 MHz $^{13}$C-NMR of Compound (**2a**) in $CDCl_3$

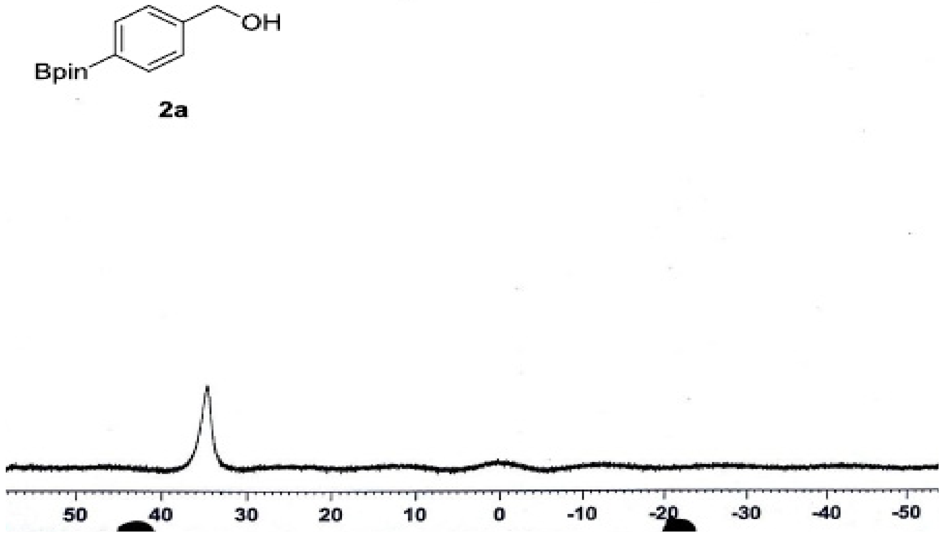


192.5 MHz $^{11}$B-NMR of Compound (**2a**) in $CDCl_3$

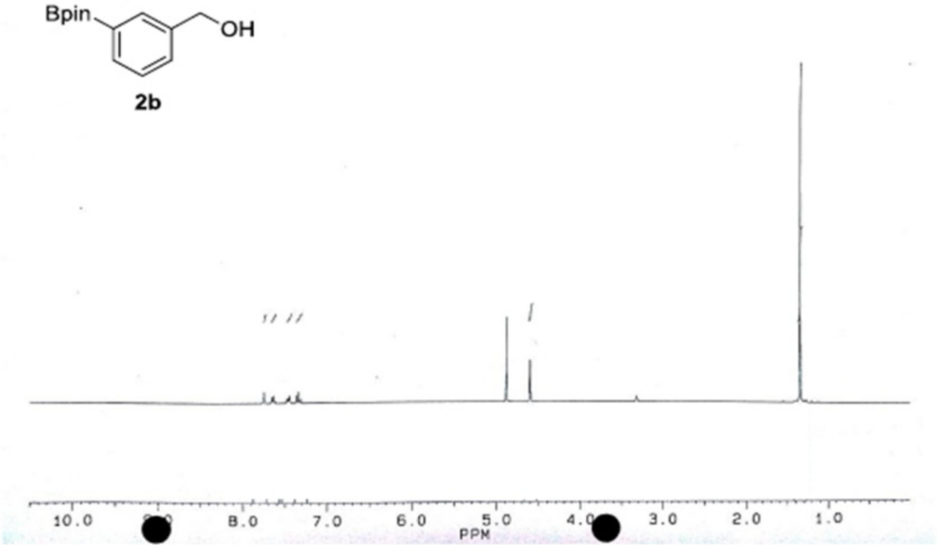


300 MHz $^{1}$H-NMR of Compound (**2b**) in $CDCl_3$

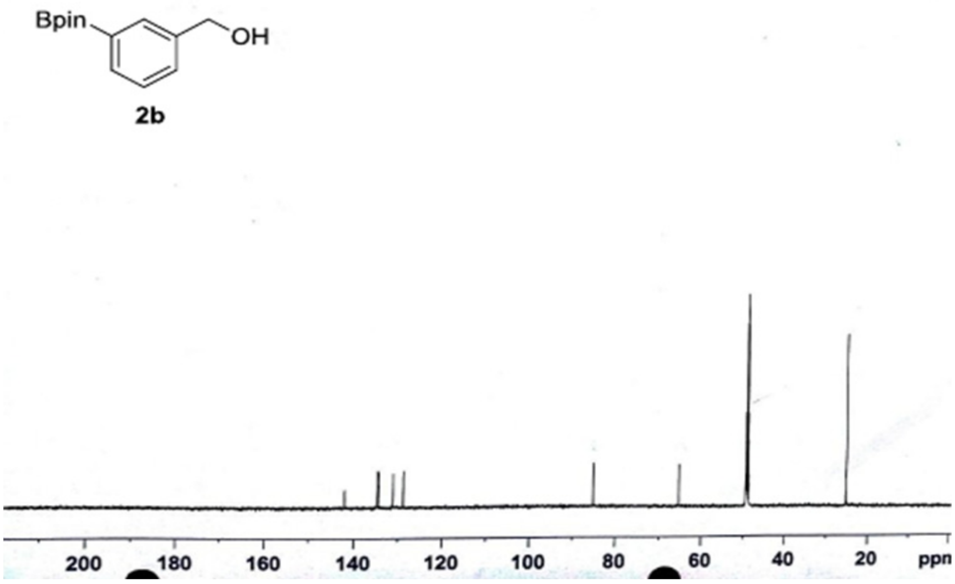


150.9 MHz $^{13}$C-NMR of Compound (**2b**) in $CDCl_3$

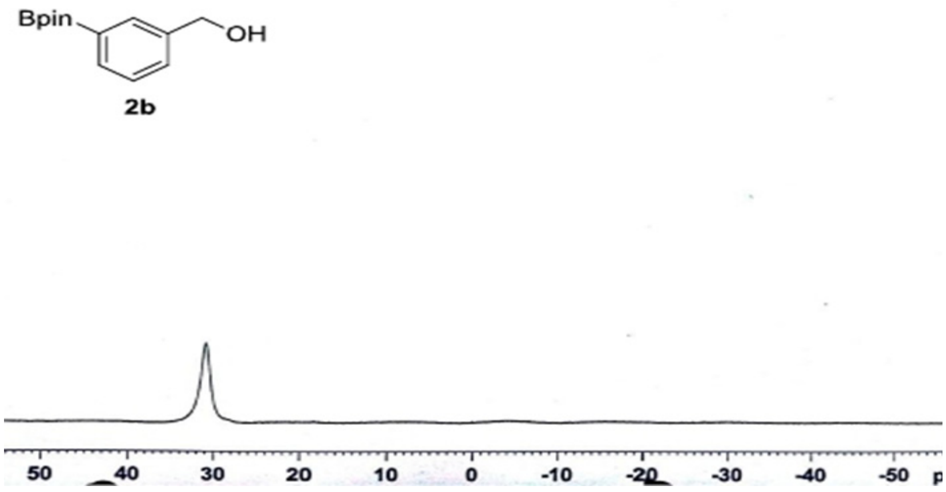


192.5 MHz $^{11}$B-NMR of Compound (**2b**) in $CDCl_3$

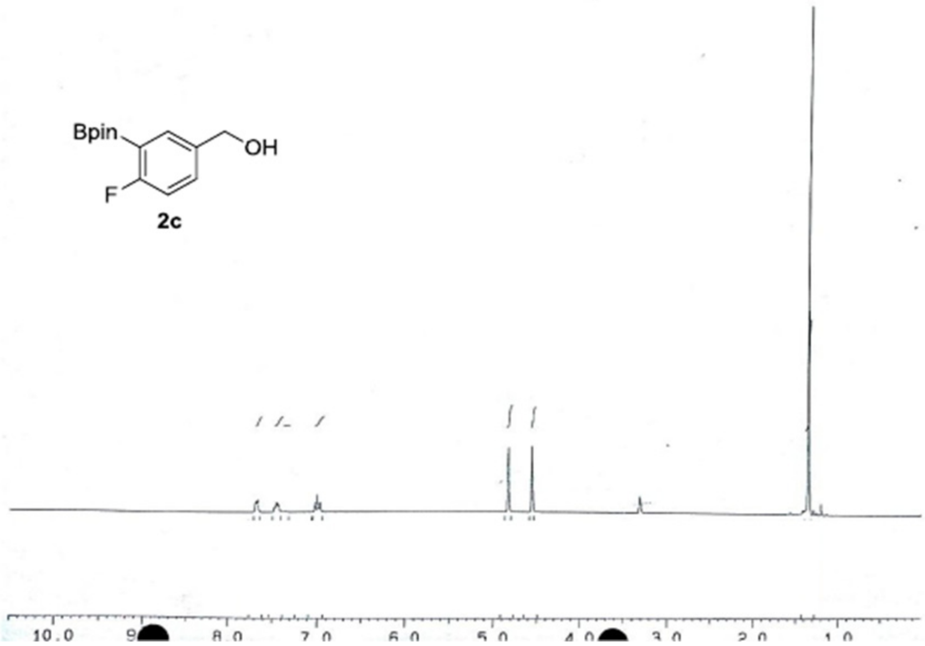


300 MHz ¹H-NMR of Compound (**2c**) in $CD_3OD$-*d*$_4$

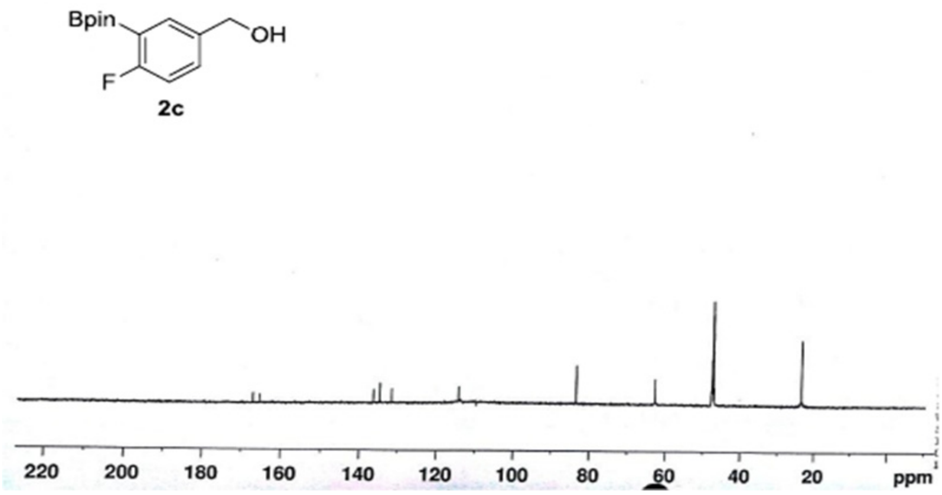


150.9 MHz ¹³C-NMR of Compound (**2c**) in $CD_3OD$-*d*$_4$

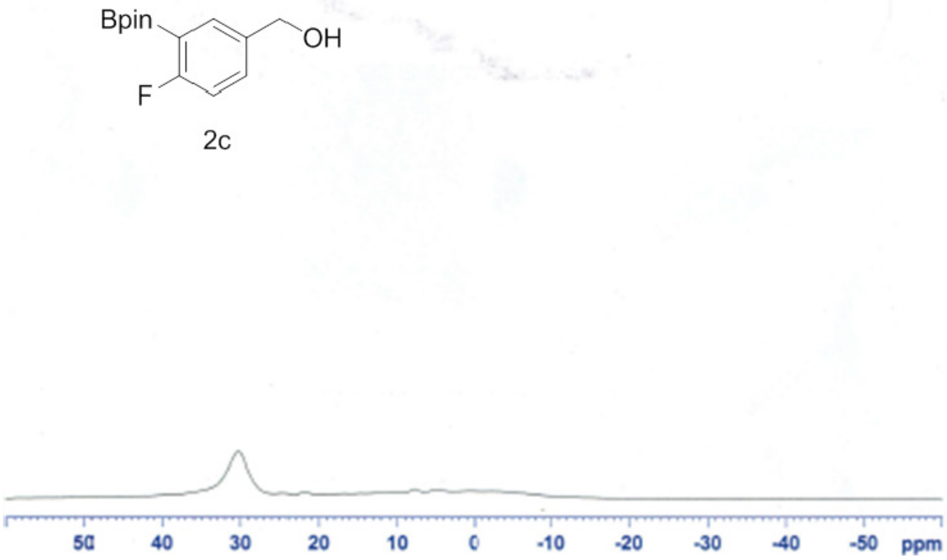


192.5 MHz $^{11}B$-NMR of Compound (**2c**) in $CDCl_3$

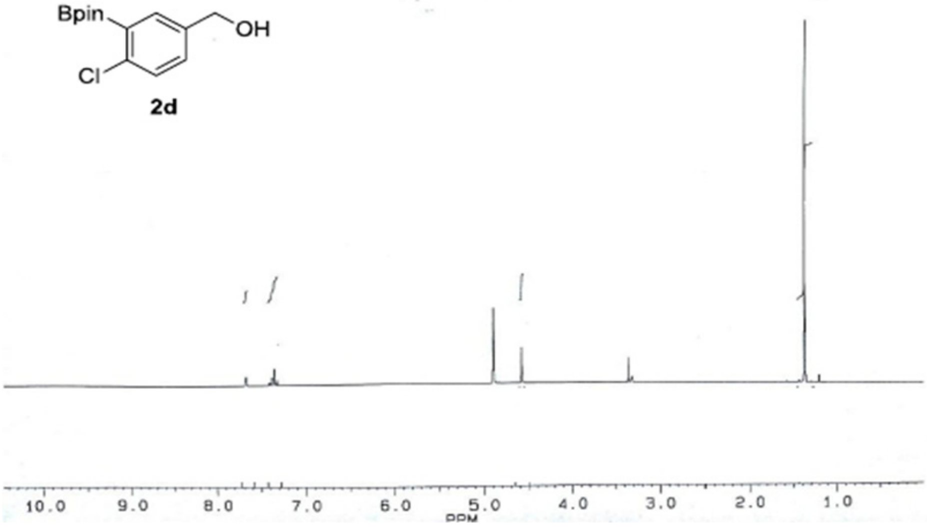


300 MHz $^{1}H$-NMR of Compound (**2d**) in $CD_3OD$-*d*

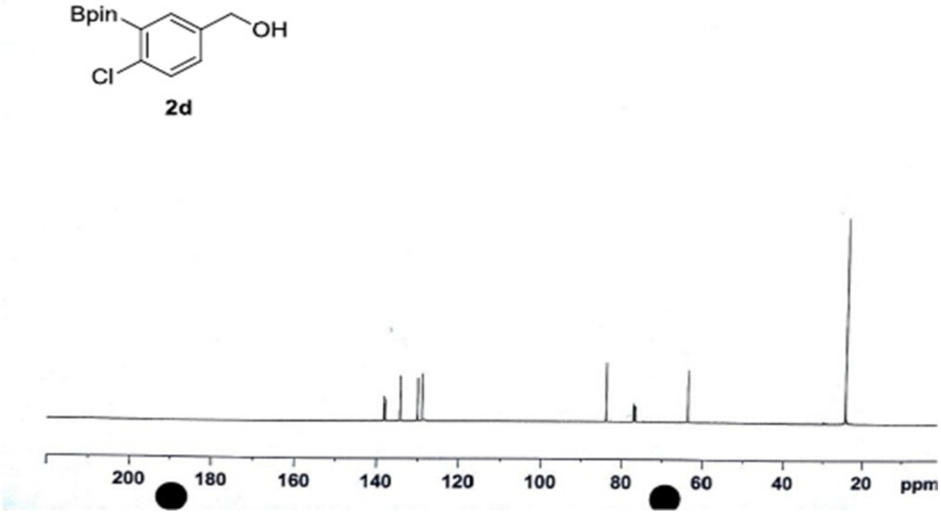


75.5 MHz $^{13}$C-NMR of Compound (**2d**) in $CDCl_3$

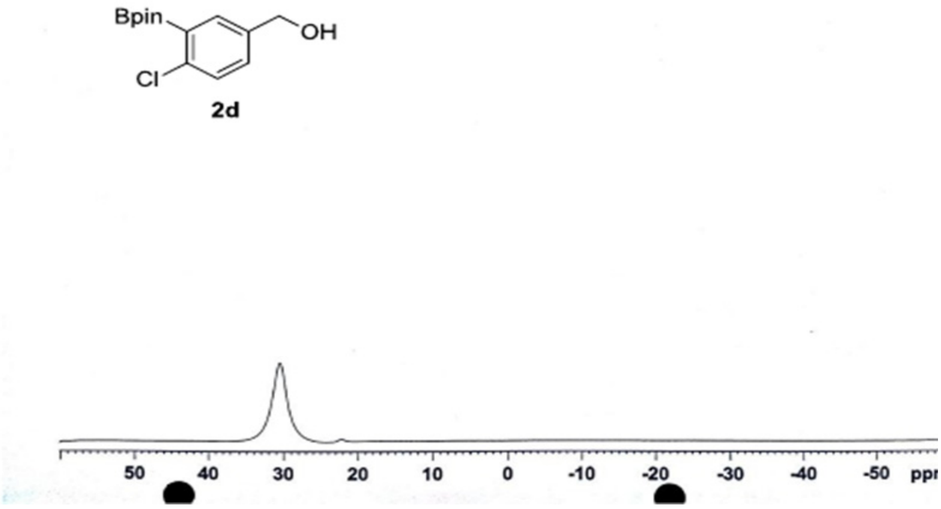


192.5 MHz $^{11}$B-NMR of Compound (**2d**) in $CDCl_3$

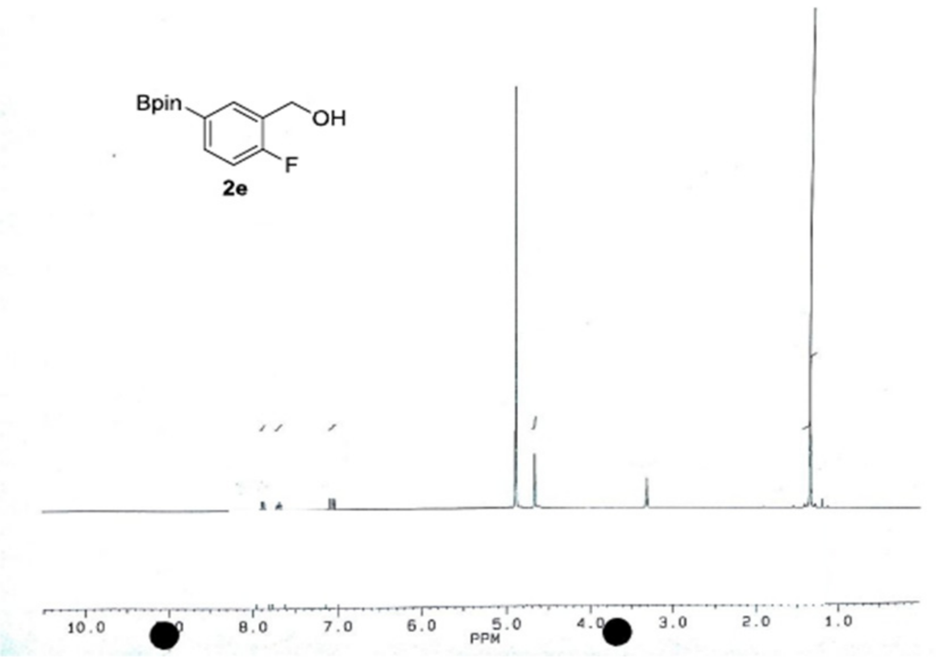


300 MHz $^{1}$H-NMR of Compound (**2e**) in $CD_3OD$-*$d_4$*

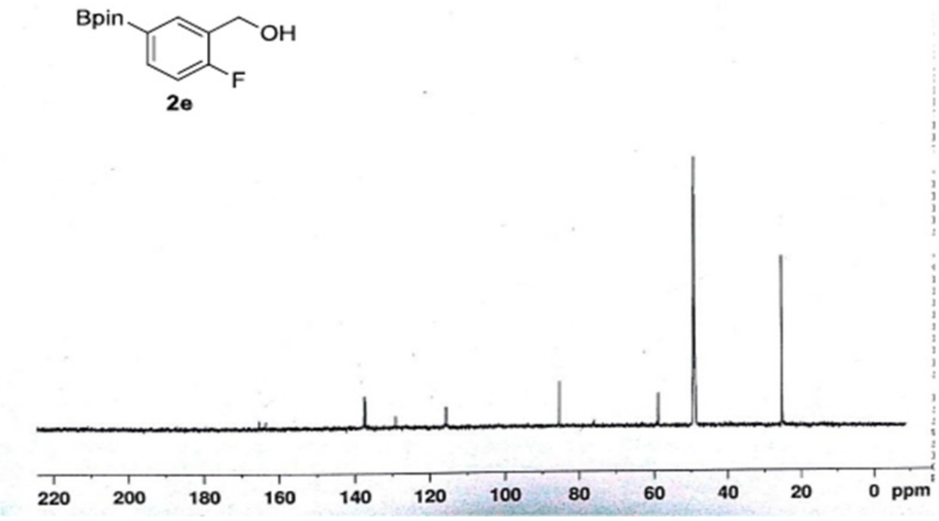


150.9 MHz $^{13}$C-NMR of Compound (**2e**) in $CD_3OD$-*$d_4$*

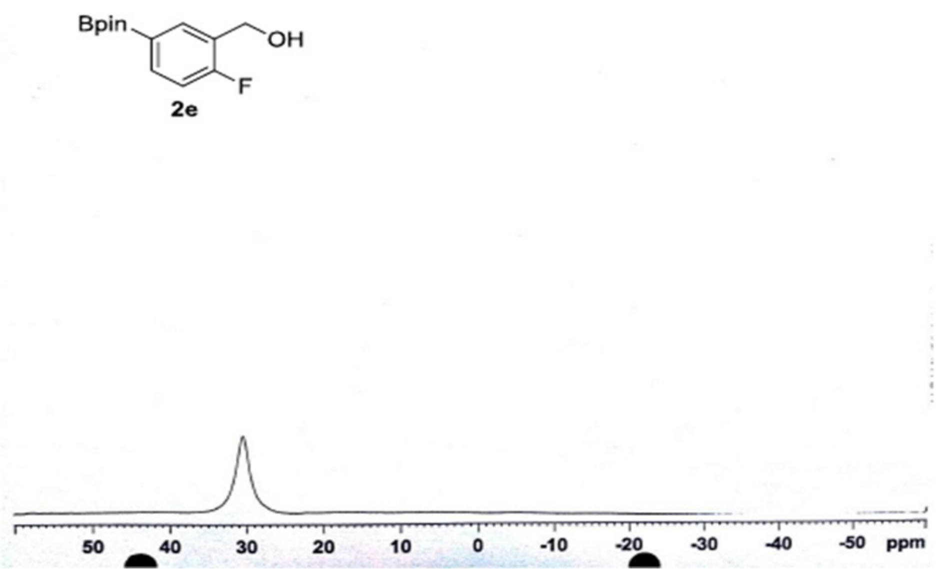


192.5 MHz $^{11}$B-NMR of Compound (**2e**) in $CDCl_3$

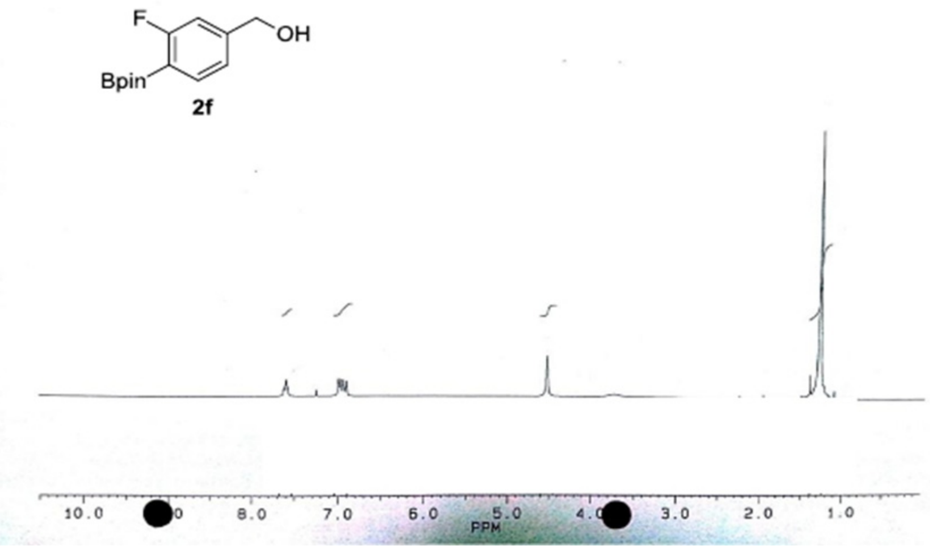


300 MHz $^{1}$H-NMR of Compound (**2f**) in $CDCl_3$

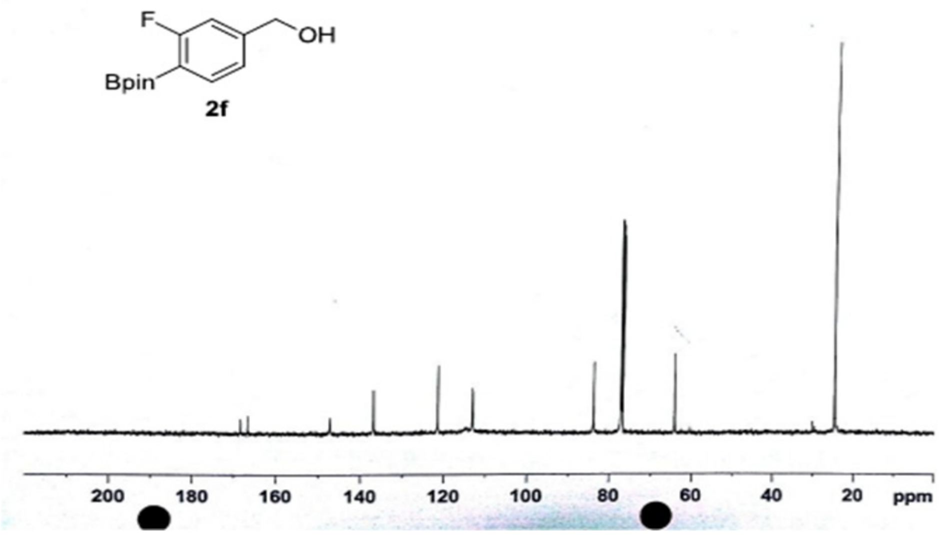


150.9 MHz $^{13}C$-NMR of Compound (**2f**) in $CDCl_3$

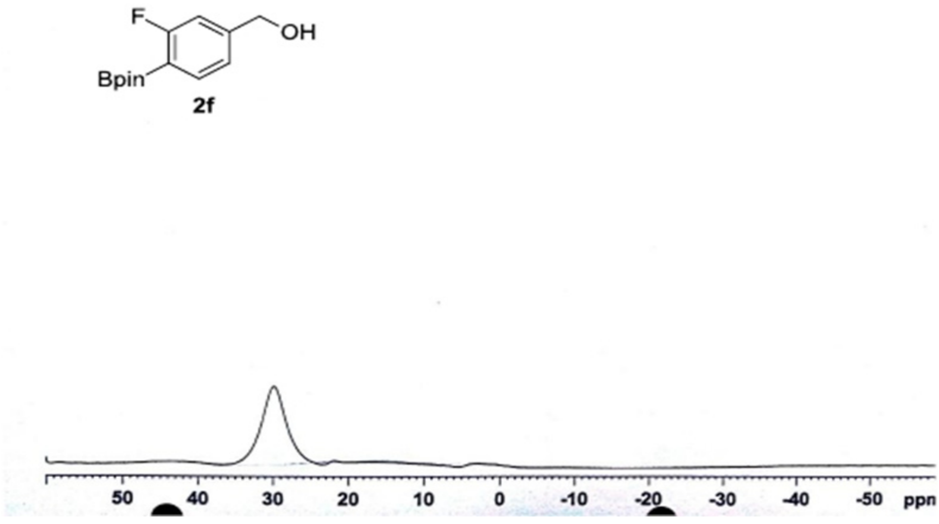


192.5 MHz $^{11}B$-NMR of Compound (**2f**) in $CDCl_3$

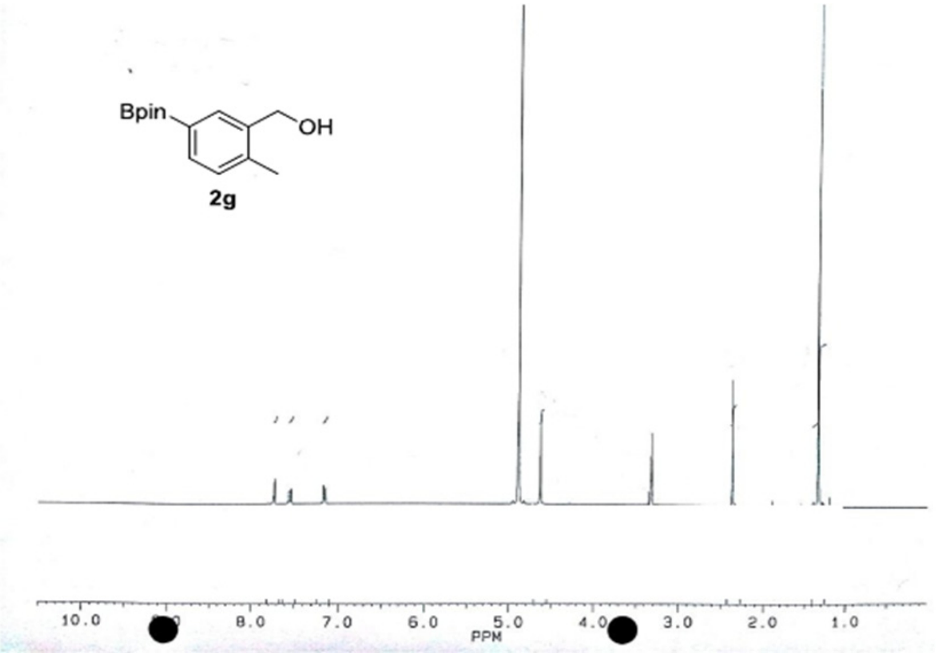


300 MHz $^{1}$H-NMR of Compound (**2g**) in $CD_3OD$-$d_4$

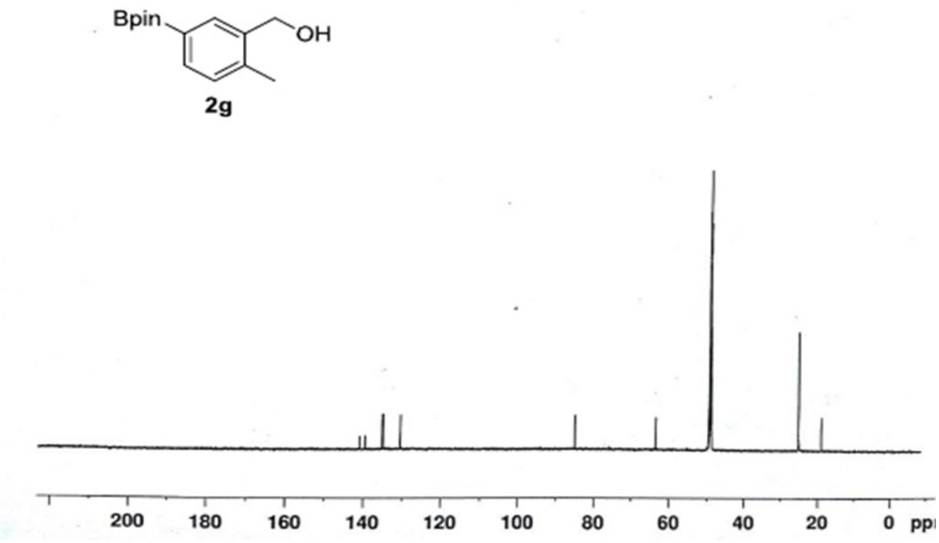


150.9 MHz $^{13}$C-NMR of Compound (**2g**) in $CD_3OD$-$d_4$

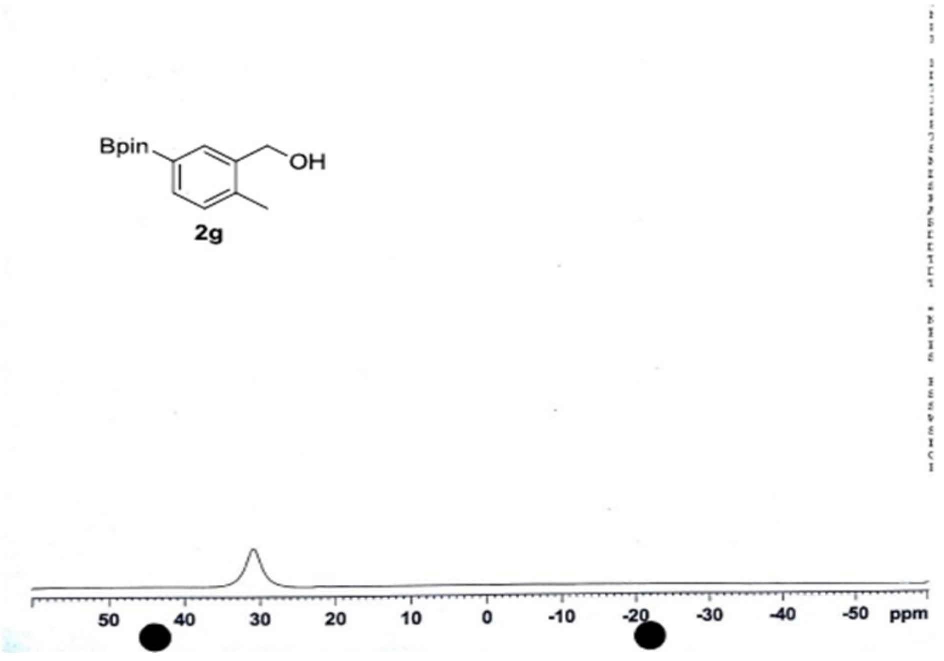


192.5 MHz $^{11}$B-NMR of Compound (**2g**) in $CDCl_3$

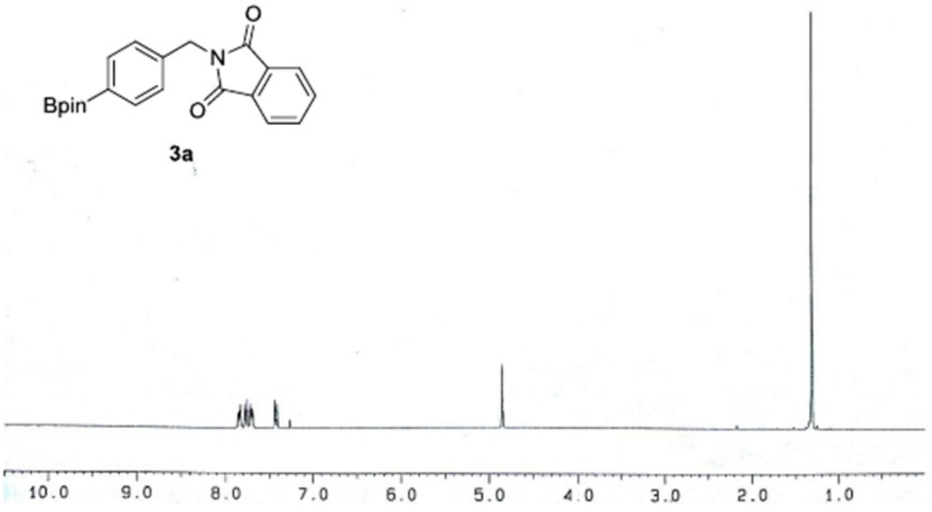


300 MHz $^{1}$H-NMR of Compound (**3a**) in $CDCl_3$

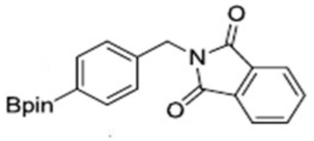


**3a**

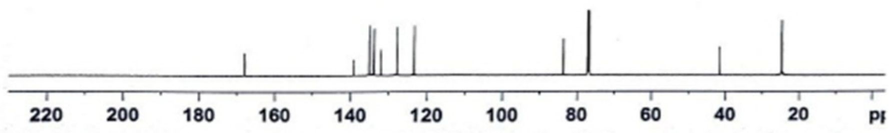


150.9 MHz $^{13}$C-NMR of Compound (**3a**) in $CDCl_3$

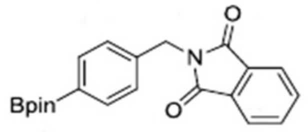


**3a**

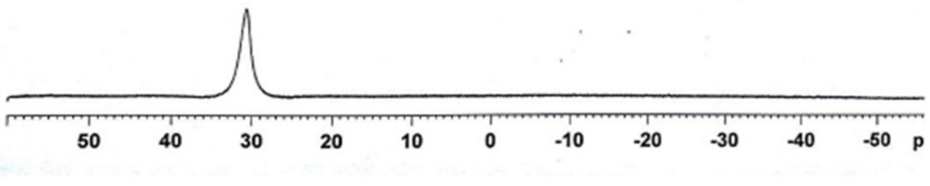


192.5 MHz $^{11}$B-NMR of Compound (**3a**) in $CDCl_3$

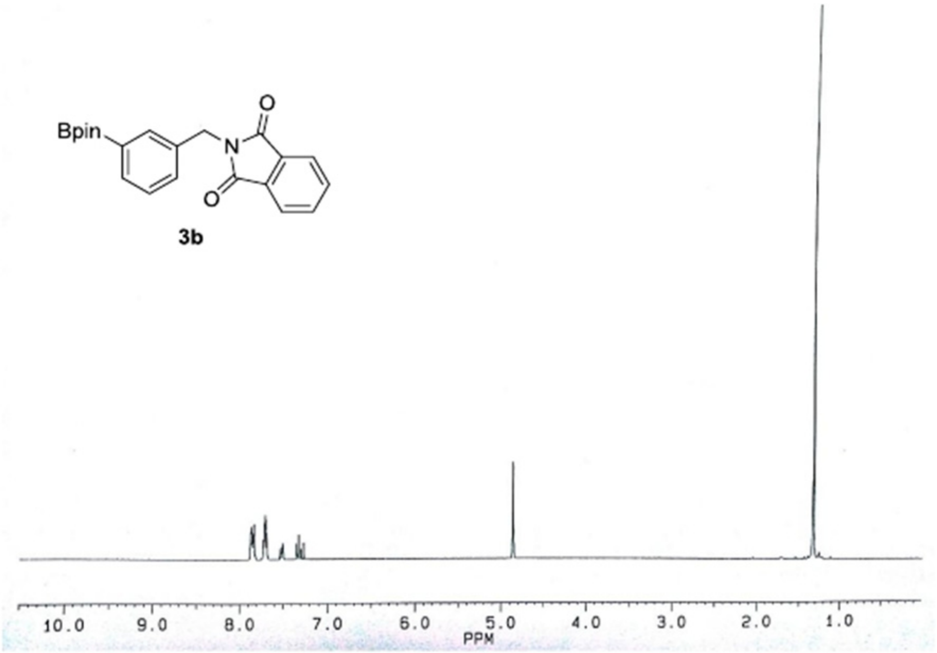


300 MHz $^{1}H$-NMR of Compound (**3b**) in $CDCl_3$

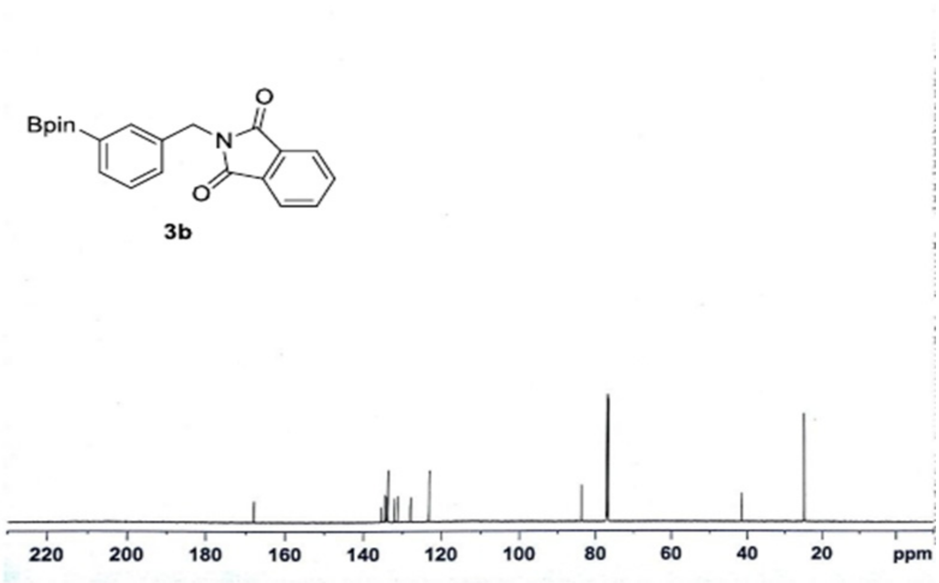


150.9 MHz $^{13}C$-NMR of Compound (**3b**) in $CDCl_3$

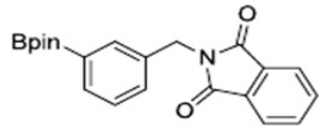

**3b**

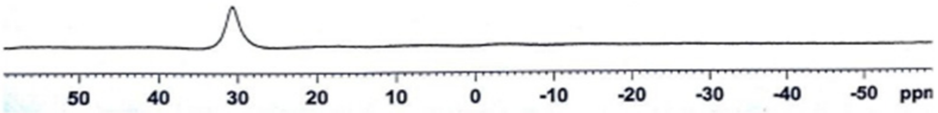


192.5 MHz $^{11}$B-NMR of Compound (**3b**) in $CDCl_3$

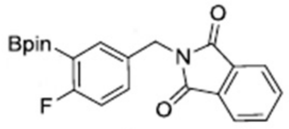

**3c**

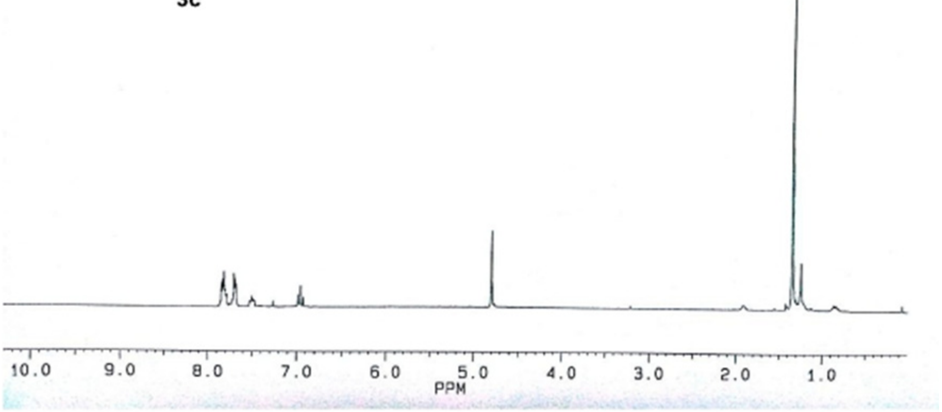


300 MHz $^{1}$H-NMR of Compound (**3c**) in $CDCl_3$

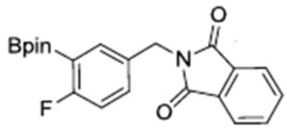


**3c**

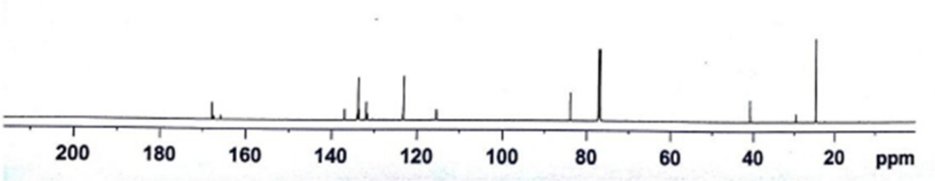


150.9 MHz $^{13}$C-NMR of Compound (**3c**) in $CDCl_3$

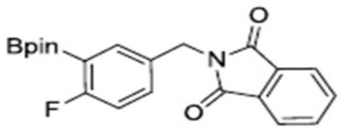


**3c**

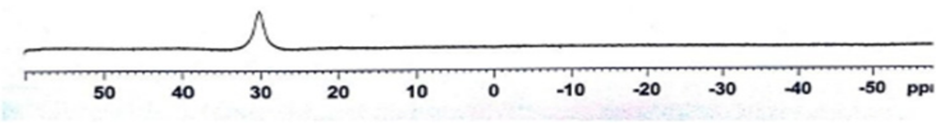


192.5 MHz $^{11}$B-NMR of Compound (**3c**) in $CDCl_3$

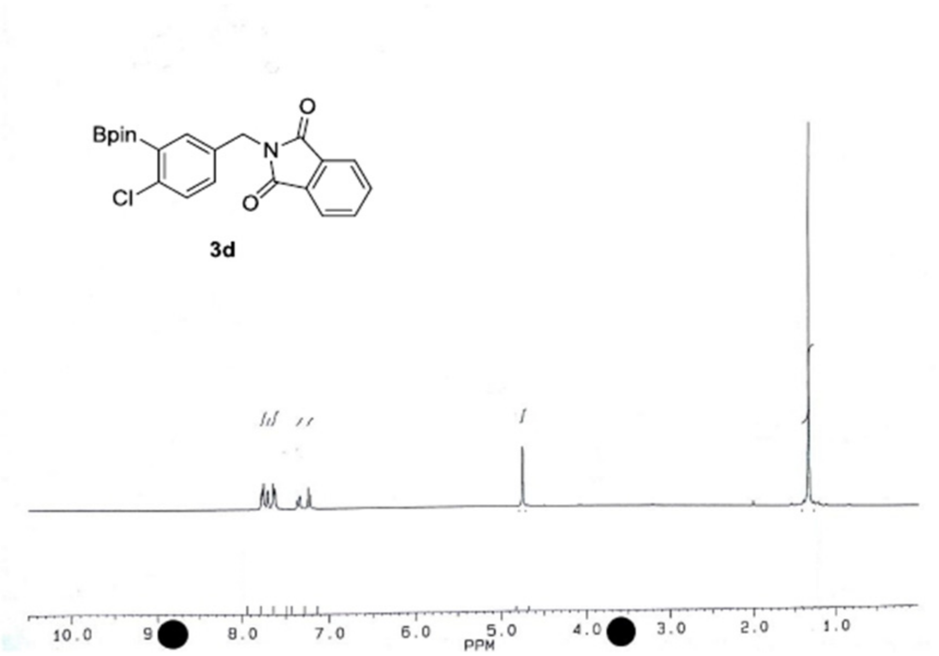


300 MHz $^{1}$H-NMR of Compound (**3d**) in $CDCl_3$

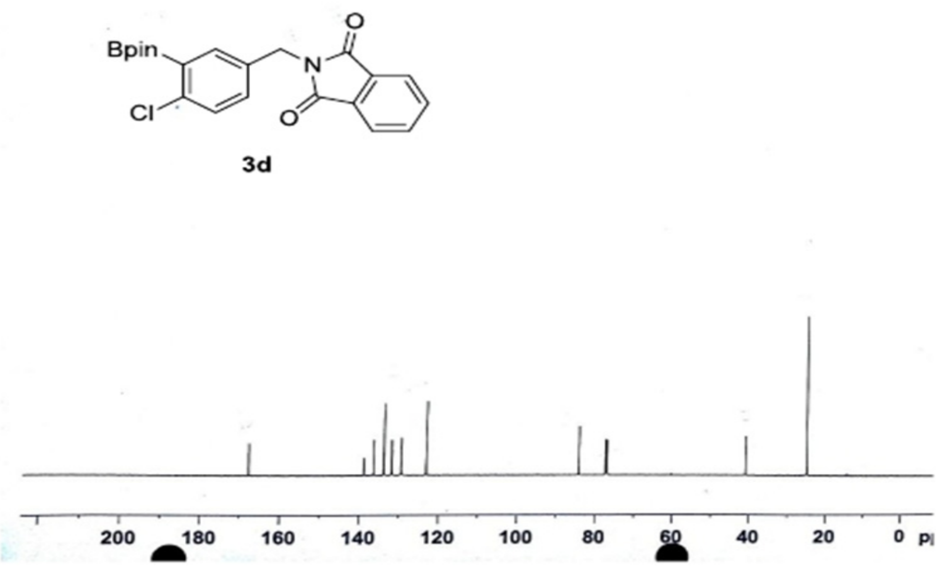


75.5 MHz $^{13}$C-NMR of Compound (**3d**) in $CDCl_3$

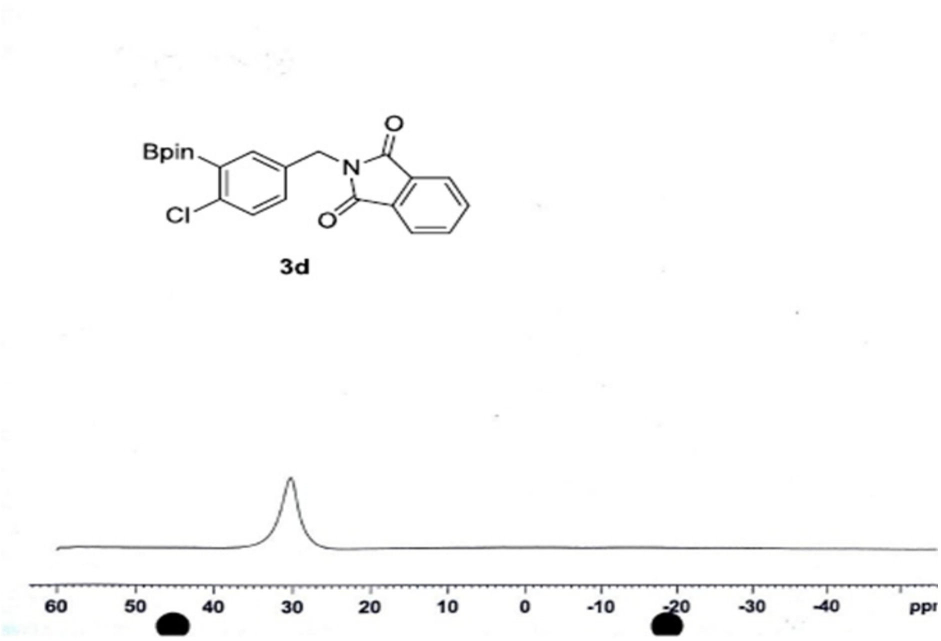


192.5 MHz $^{11}$B-NMR of Compound (**3d**) in $CDCl_3$

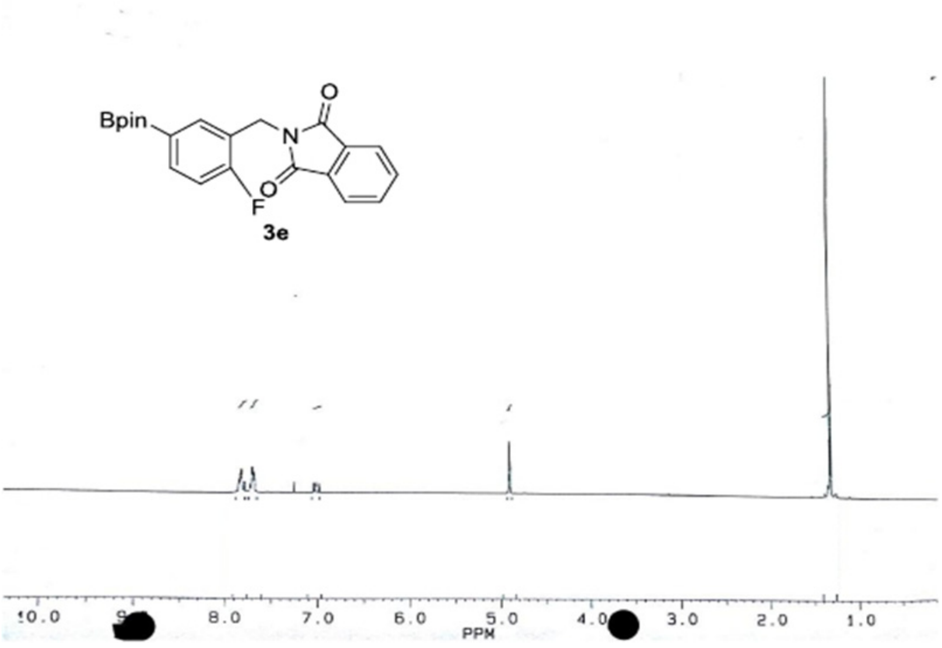


300 MHz $^{1}$H-NMR of Compound (**3e**) in $CDCl_3$

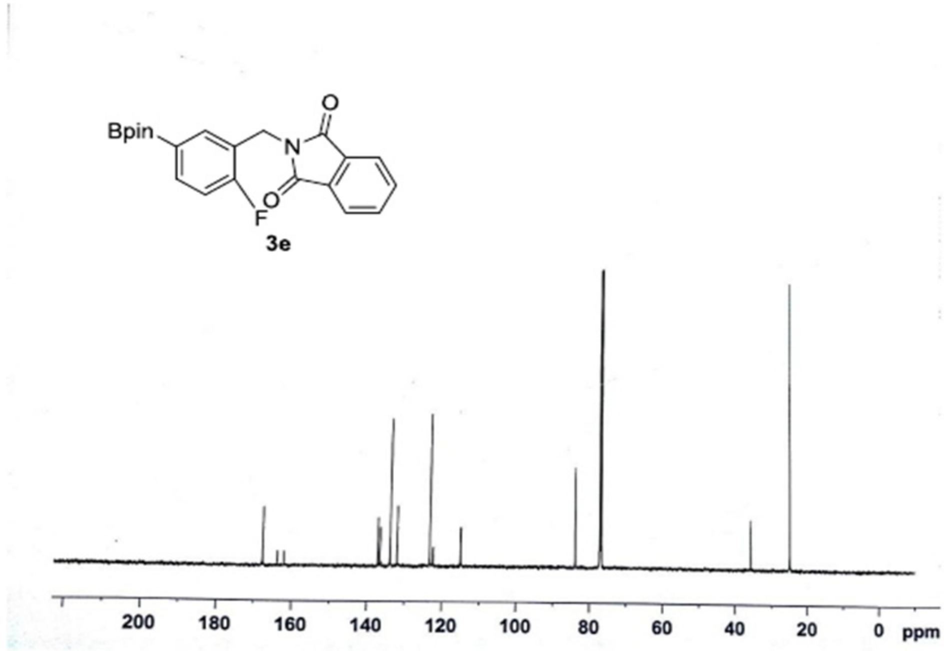


150.9 MHz $^{13}$C-NMR of Compound (**3e**) in $CDCl_3$

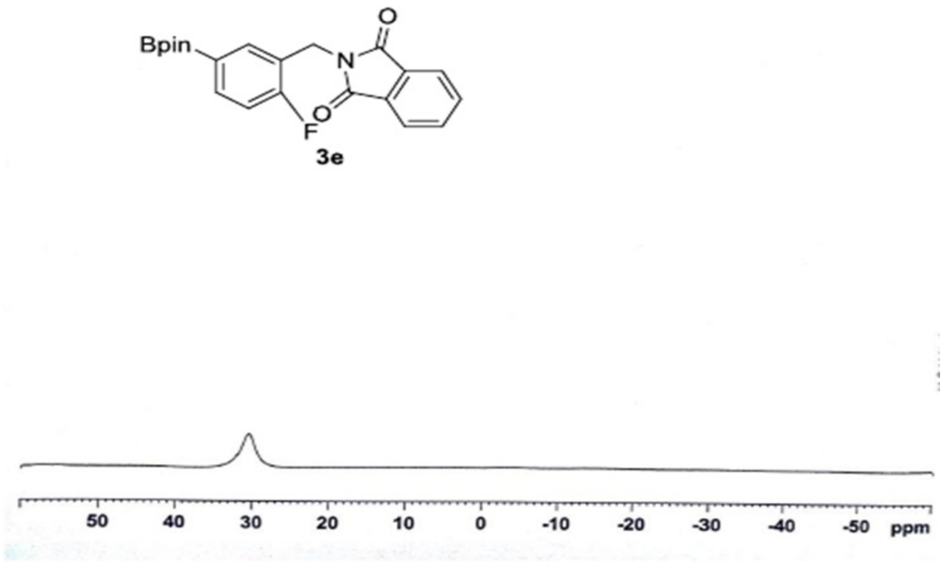


192.5 MHz $^{11}$B-NMR of Compound (**3e**) in $CDCl_3$

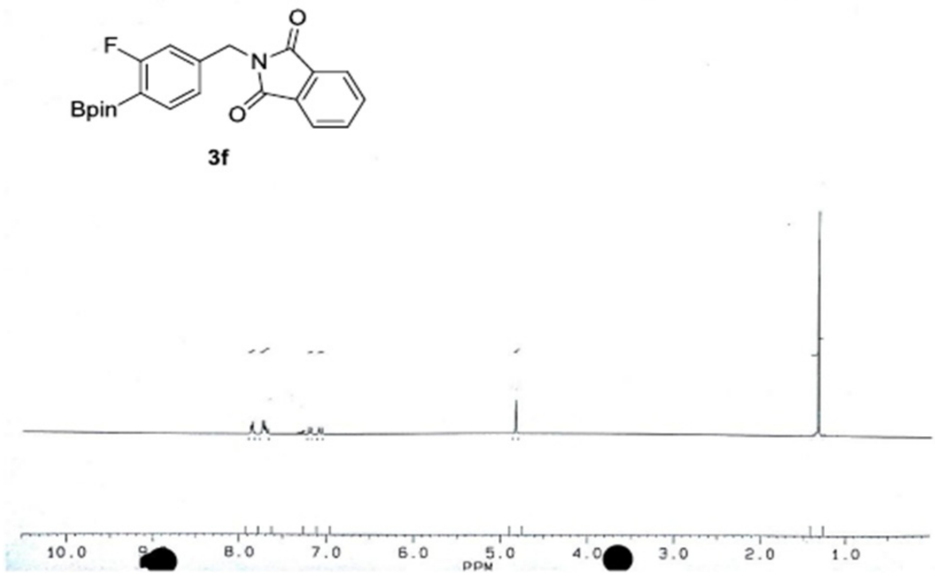


300 MHz $^{1}$H-NMR of Compound (**3f**) in $CDCl_3$

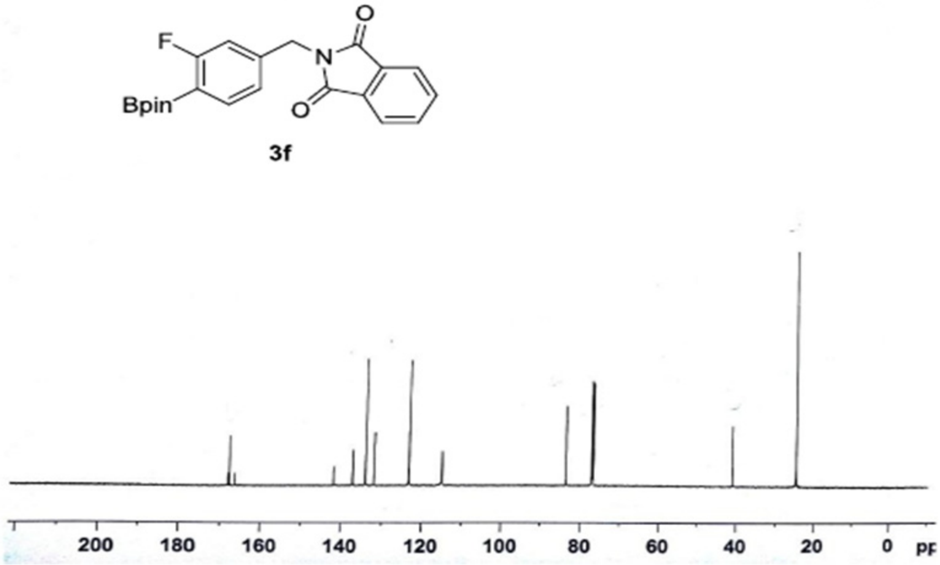


75.5 MHz $^{13}$C-NMR of Compound (**3f**) in $CDCl_3$

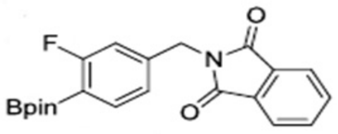


**3f**

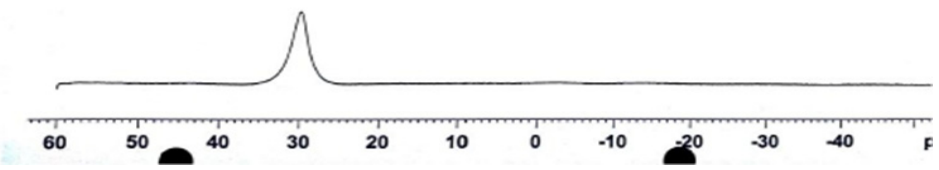


192.5 MHz $^{11}$B-NMR of Compound (**3f**) in $CDCl_3$

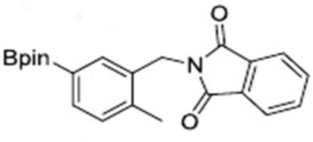


**3g**

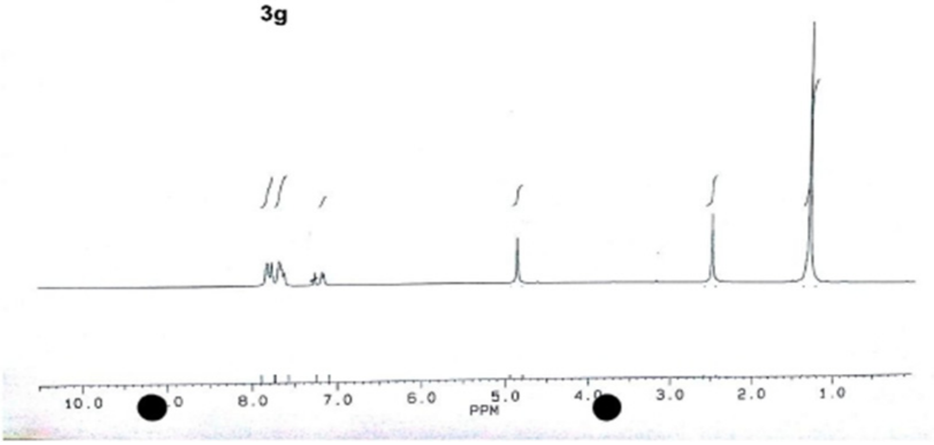


300 MHz $^{11}$H-NMR of Compound (**3g**) in $CDCl_3$

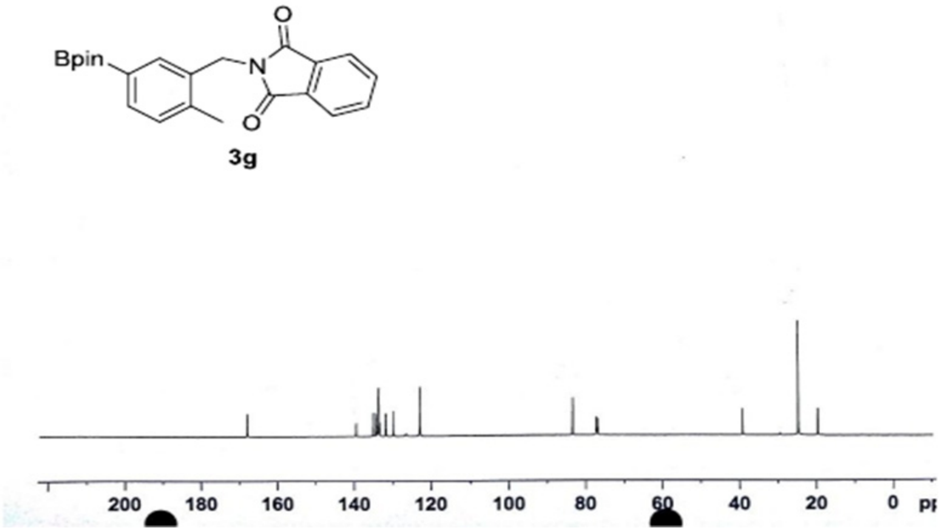


150.9 MHz $^{13}$C-NMR of Compound (**3g**) in $CDCl_3$

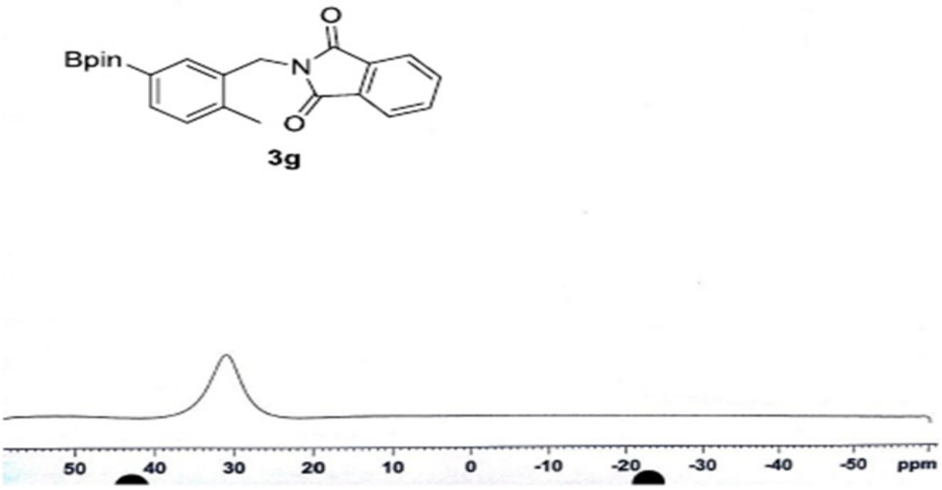


192.5 MHz $^{11}$B-NMR of Compound (**3g**) in $CDCl_3$

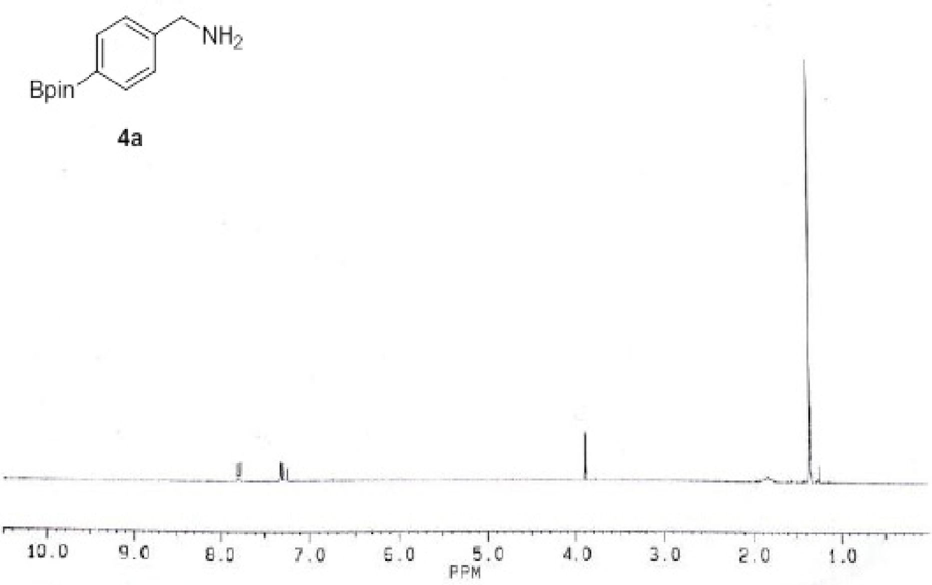


300 MHz $^{1}$H-NMR of Compound (**4a**) in $CDCl_3$

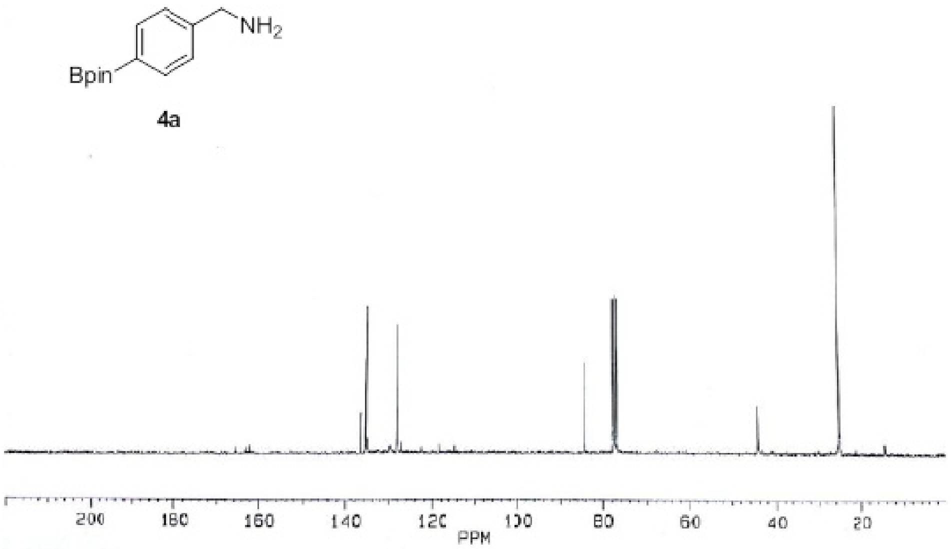


75.5 MHz $^{13}$C-NMR of Compound (**4a**) in $CDCl_3$

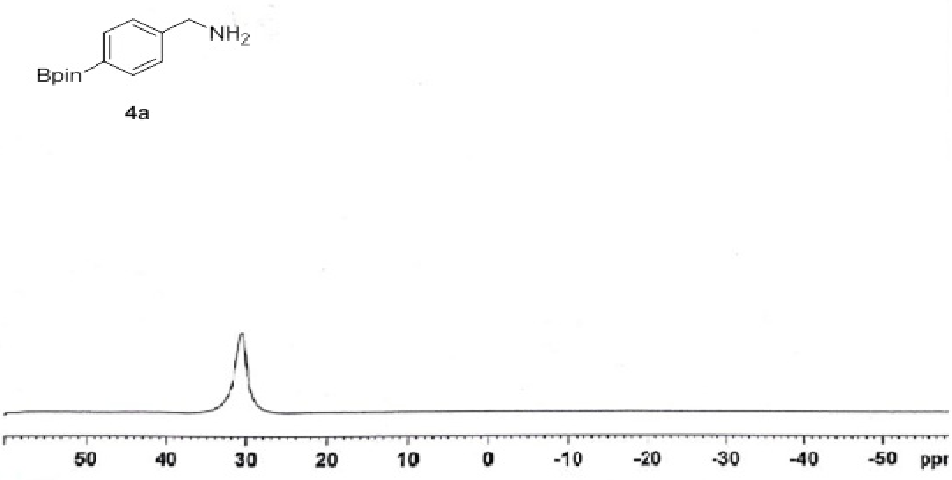


4a

192.5 MHz $^{11}$B-NMR of Compound (**4a**) in $CD_3OD$-$d_4$

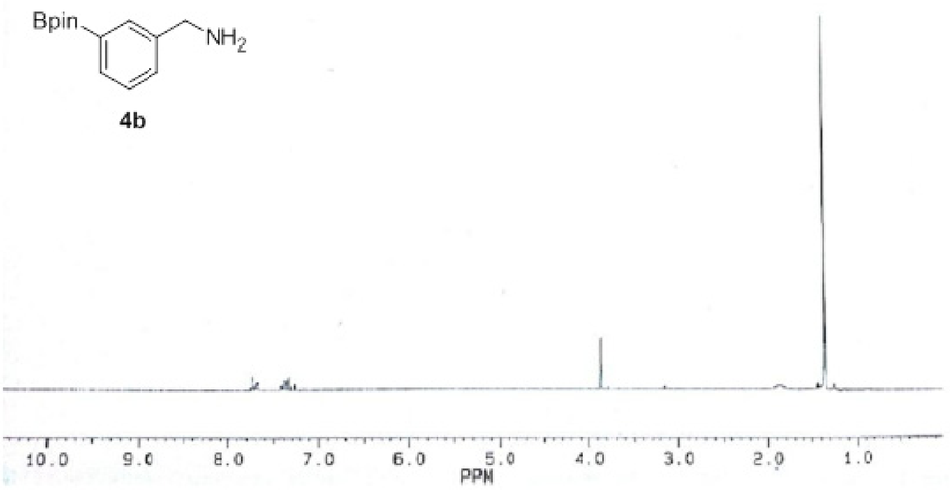


4b

300 MHz $^1$H-NMR of Compound (**4b**) in $CDCl_3$

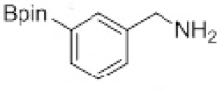


**4b**

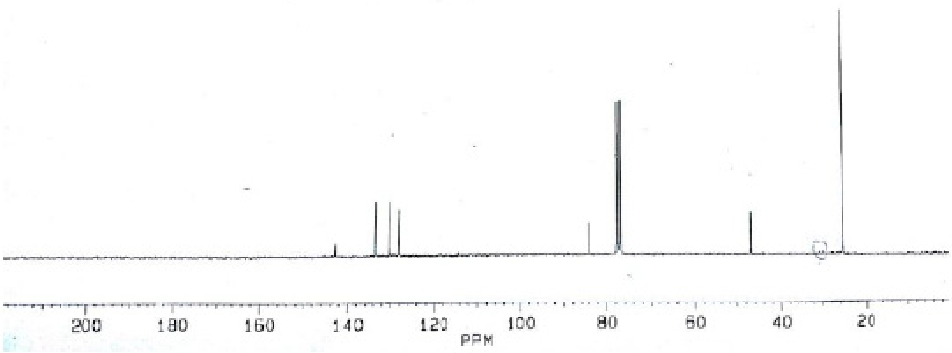


75.5 MHz $^{13}$C-NMR of Compound (**4b**) in $CDCl_3$-$d_4$

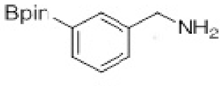


**4b**

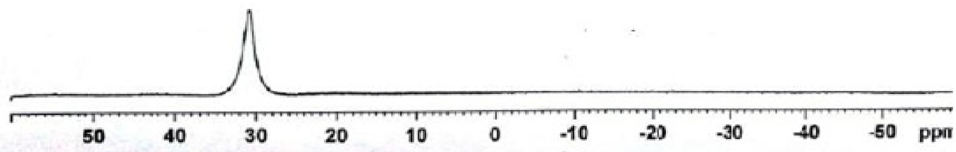


192.5 MHz $^{11}$B-NMR of Compound (**4b**) in $CD_3OD$-$d_4$

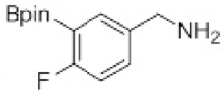


**4c**

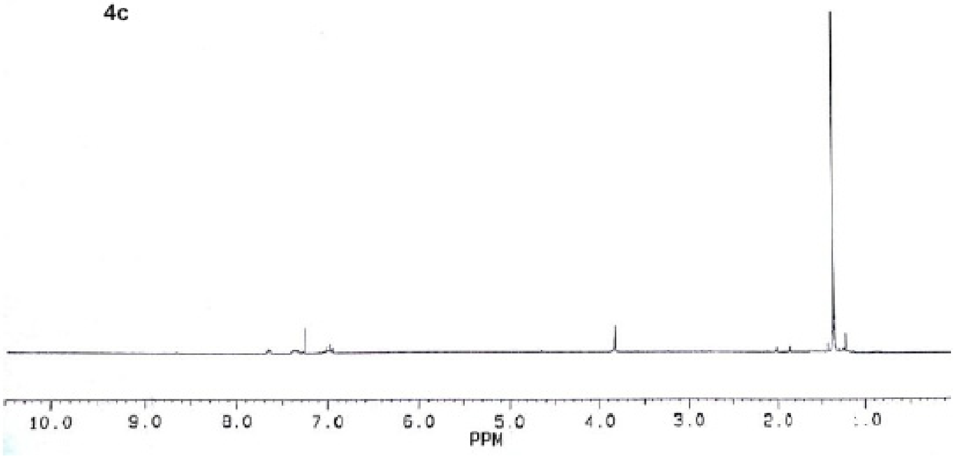


300 MHz $^{1}$H-NMR of Compound (**4c**) in $CDCl_3$

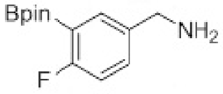


**4c**

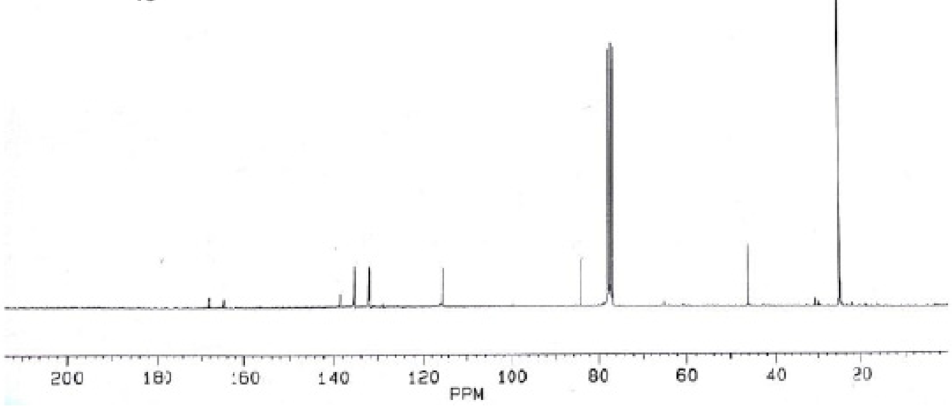


75.5 MHz $^{13}$C-NMR of Compound (**4c**) in $CDCl_3$

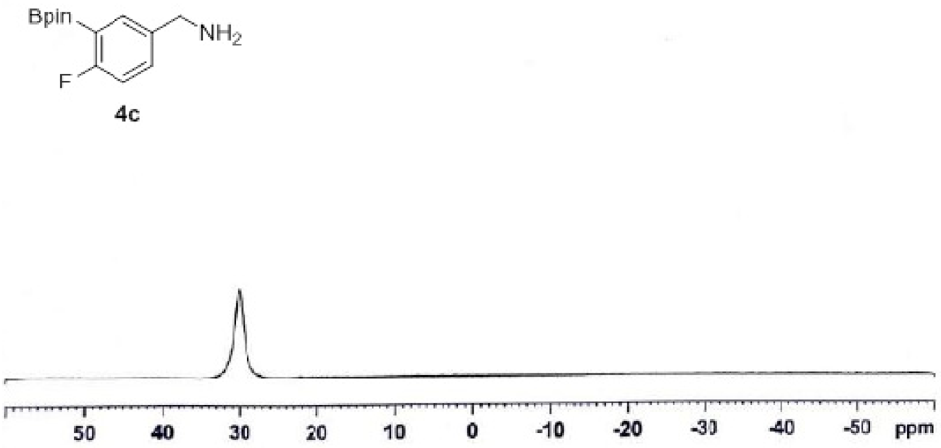


192.5 MHz $^{11}$B-NMR of Compound (**4c**) in $CD_3OD$-$d_4$

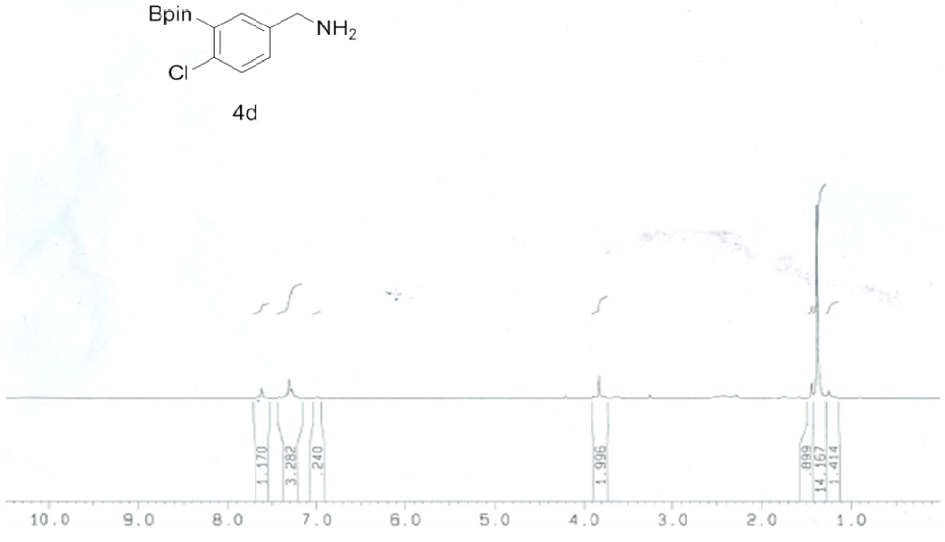


300 MHz $^{1}$H-NMR of Compound (**4d**) in $CDCl_3$

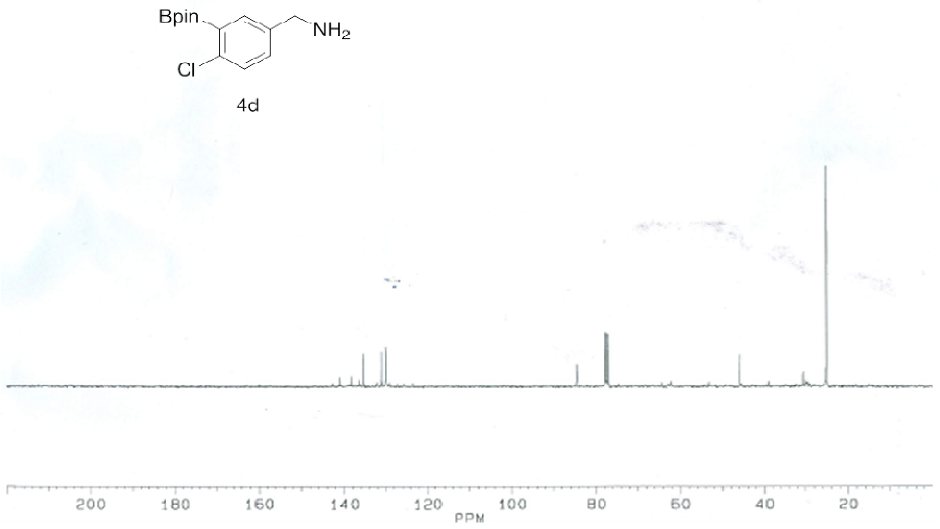


75.5 MHz $^{13}$C-NMR of Compound (**4d**) in $CDCl_3$

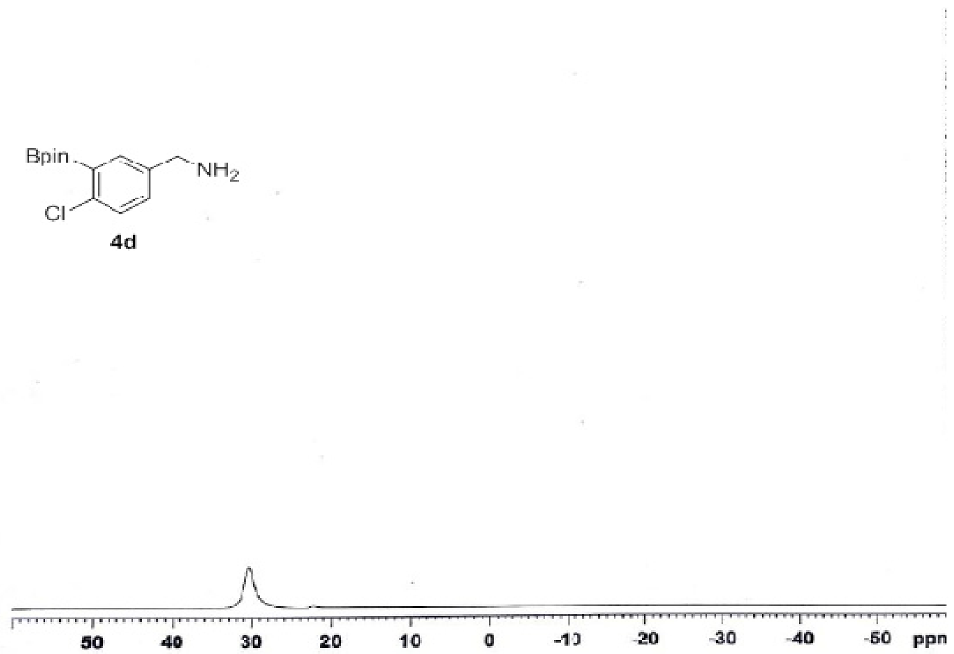


192.5 MHz $^{11}$B-NMR of Compound (**4d**) in $CD_3OD$-$d_4$

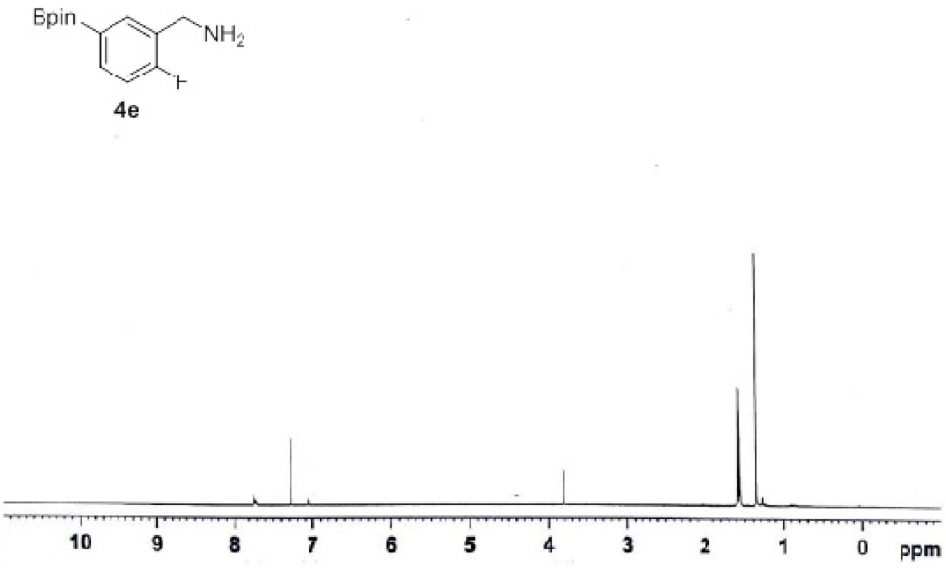


300 MHz $^{1}$H-NMR of Compound (**4e**) in $CDCl_3$

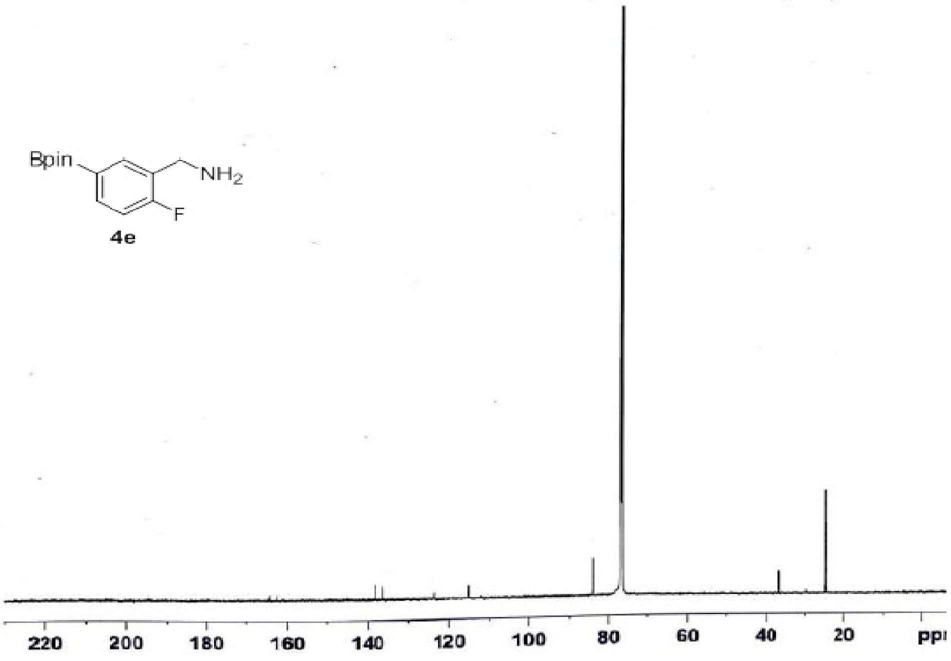


75.5 MHz $^{13}$C-NMR of Compound (**4e**) in $CDCl_3$

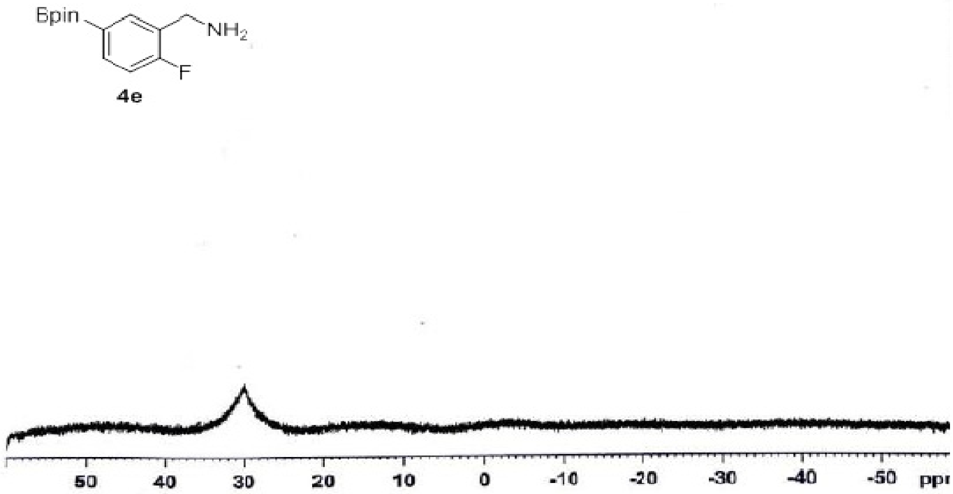


192.5 MHz $^{11}$B-NMR of Compound (**4e**) in $CD_3OD$-$d_4$

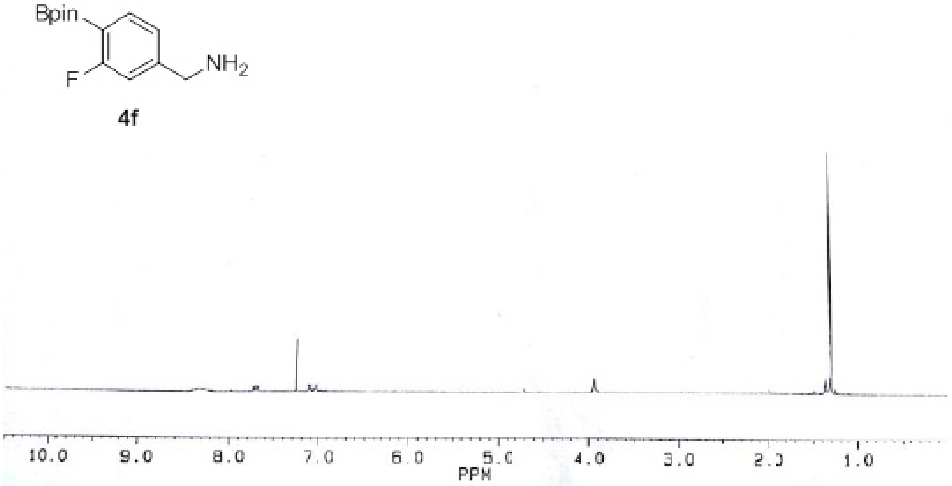


300 MHz $^{1}$H-NMR of Compound (**4f**) in $CDCl_3$

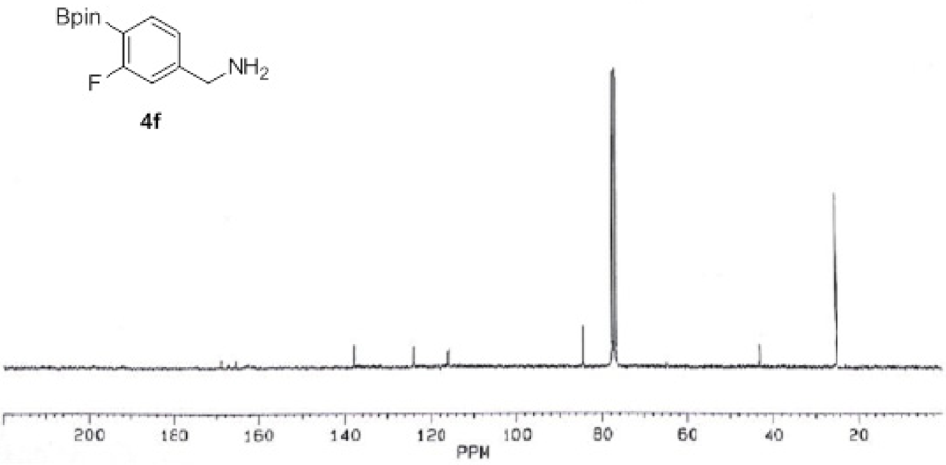


75.5 MHz $^{13}$C-NMR of Compound (**4f**) in $CDCl_3$

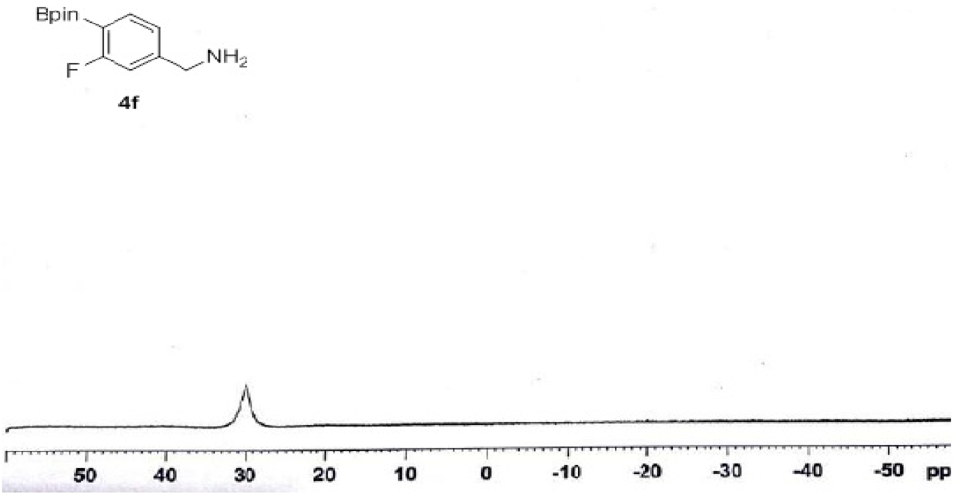


192.5 MHz $^{11}$B-NMR of Compound (**4f**) in $CDCl_3$

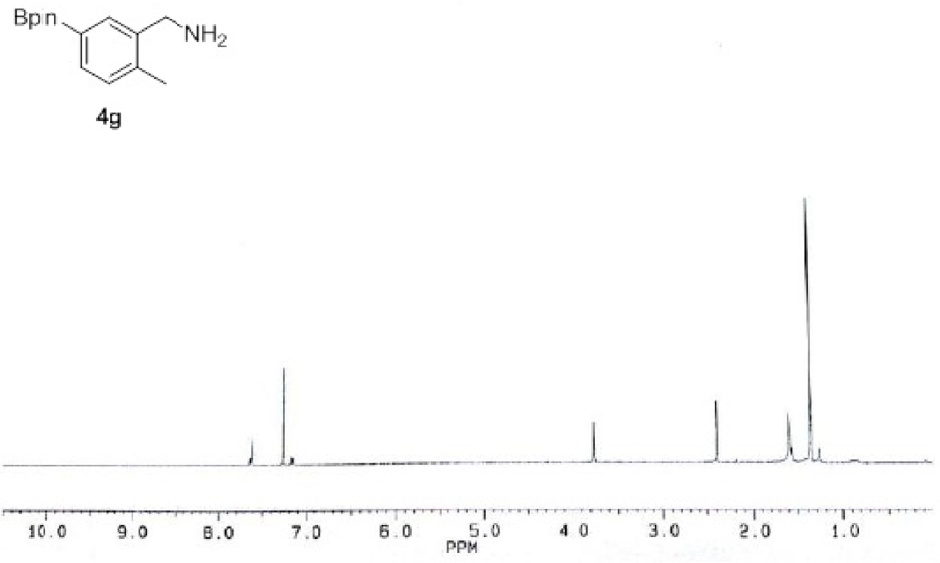


300 MHz $^{1}$H-NMR of Compound (**4g**) in $CDCl_3$

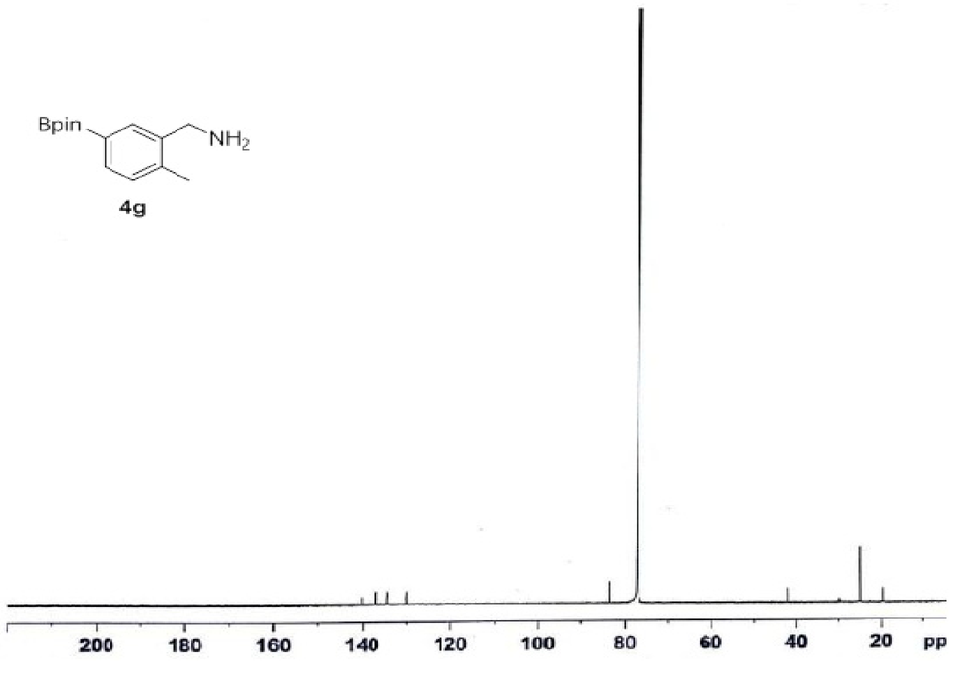


150.9 MHz $^{13}$C-NMR of Compound (**4g**) in $CDCl_3$

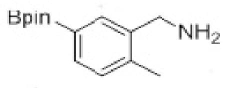


**4g**

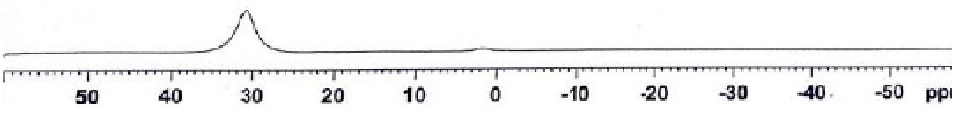


192.5 MHz $^{11}$B-NMR of Compound (**4g**) in $CDCl_3$

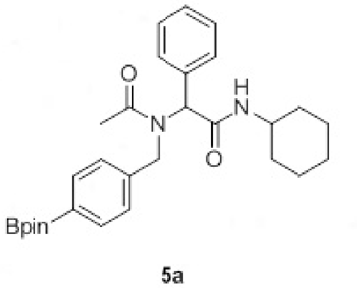


**5a**

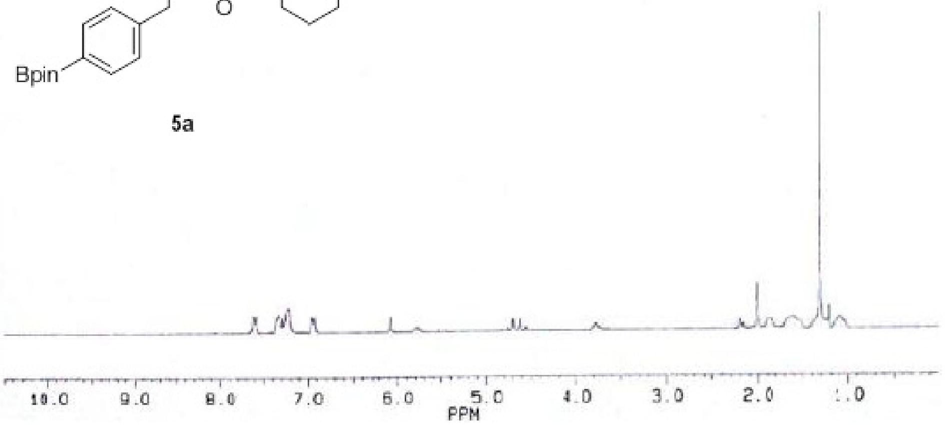


300 MHz $^{1}$H-NMR of Compound (**5a**) in $CDCl_3$-$d_3$

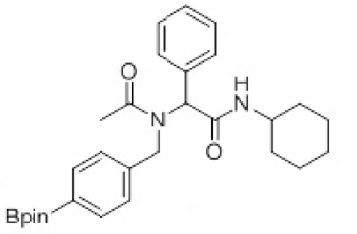


**5a**

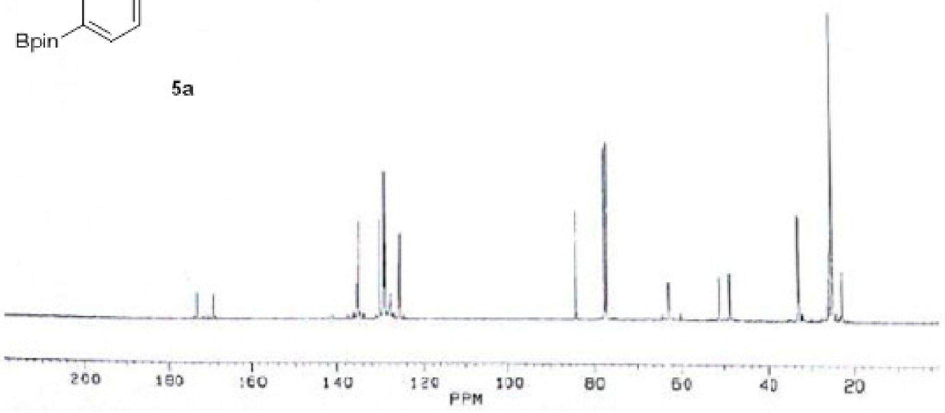


75.5 MHz $^{13}C$-NMR of Compound (**5a**) in $CDCl_3$

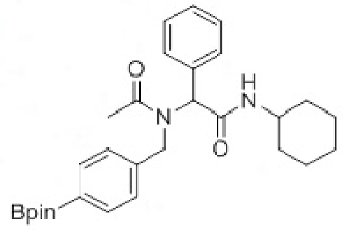


**5a**

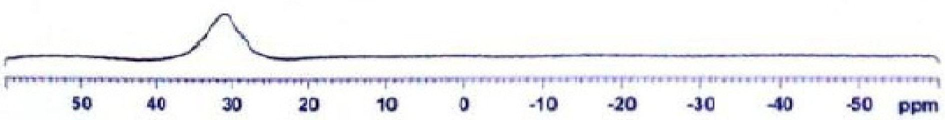


192.5 MHz $^{11}B$-NMR of Compound (**5a**) in $CD_3OD$-*d* $_4$

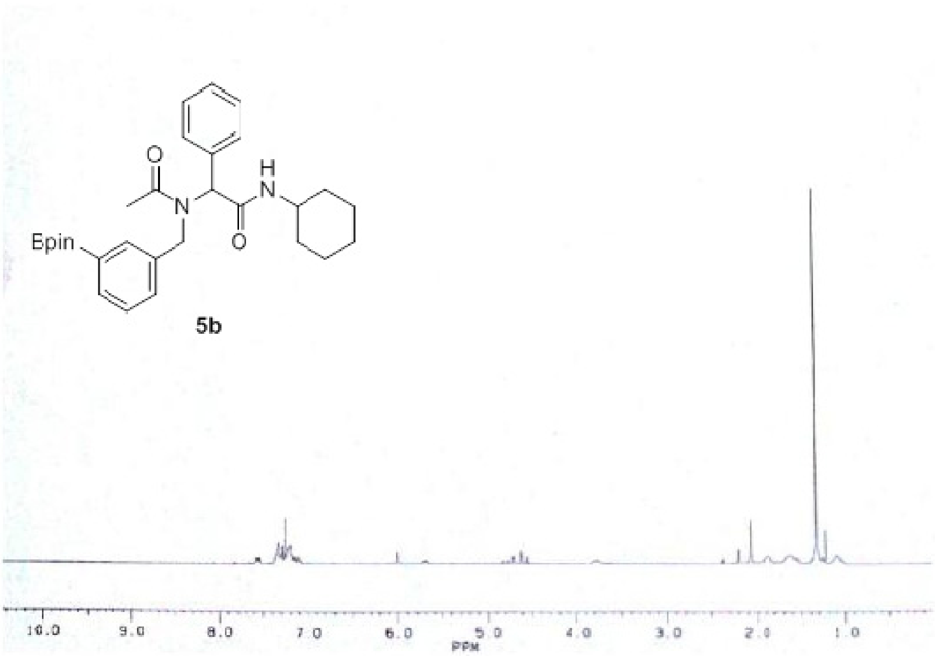


300 MHz $^{1}$H-NMR of Compound (**5b**) in $CDCl_3$

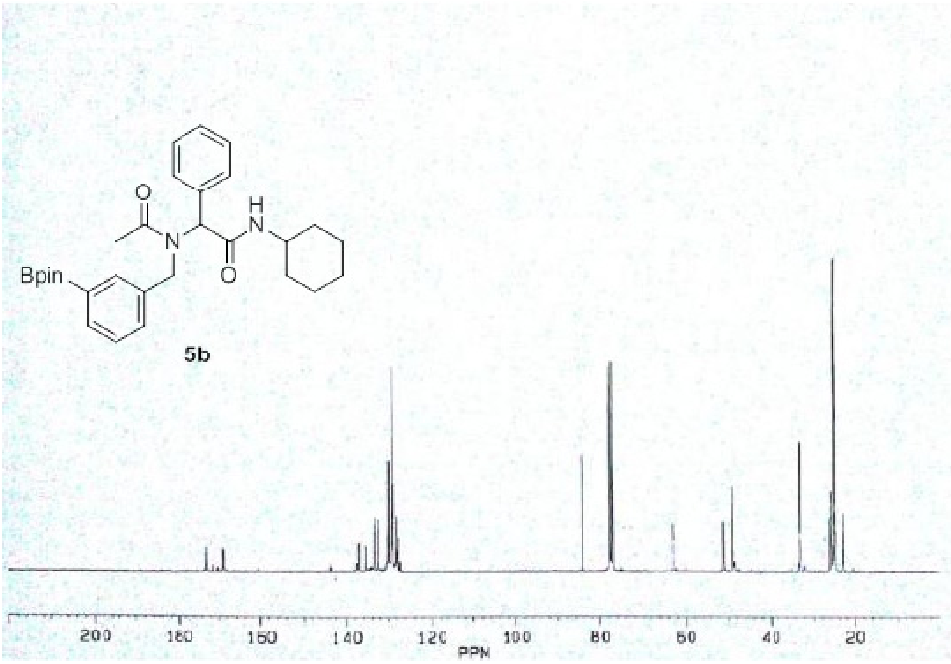


75.5 MHz $^{13}$C-NMR of Compound (**5b**) in $CDCl_3$

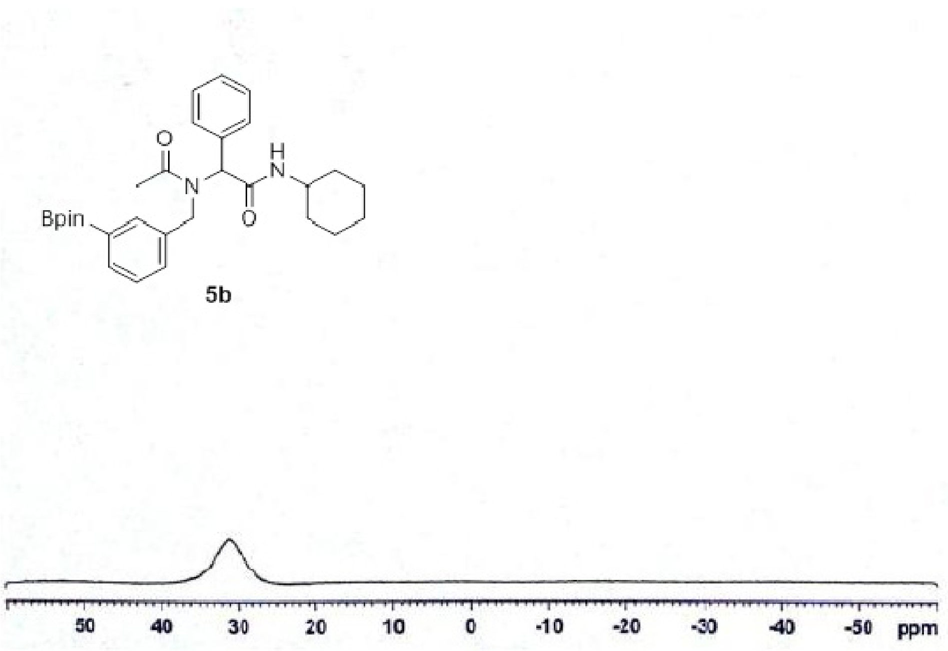


192.5 MHz $^{11}$B-NMR of Compound (**5b**) in $CD_3OD$-$d_4$

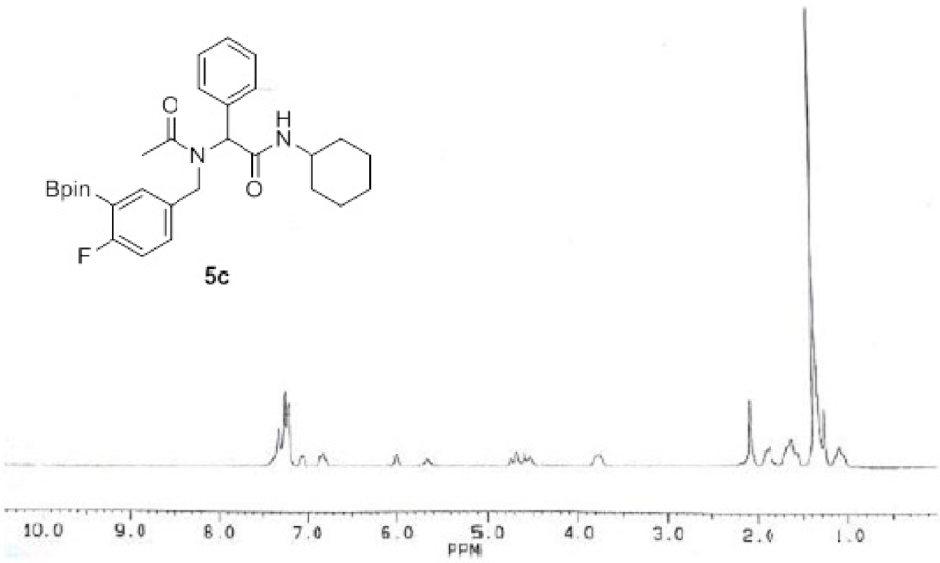


300 MHz $^{1}$H-NMR of Compound (**5c**) in $CDCl_3$

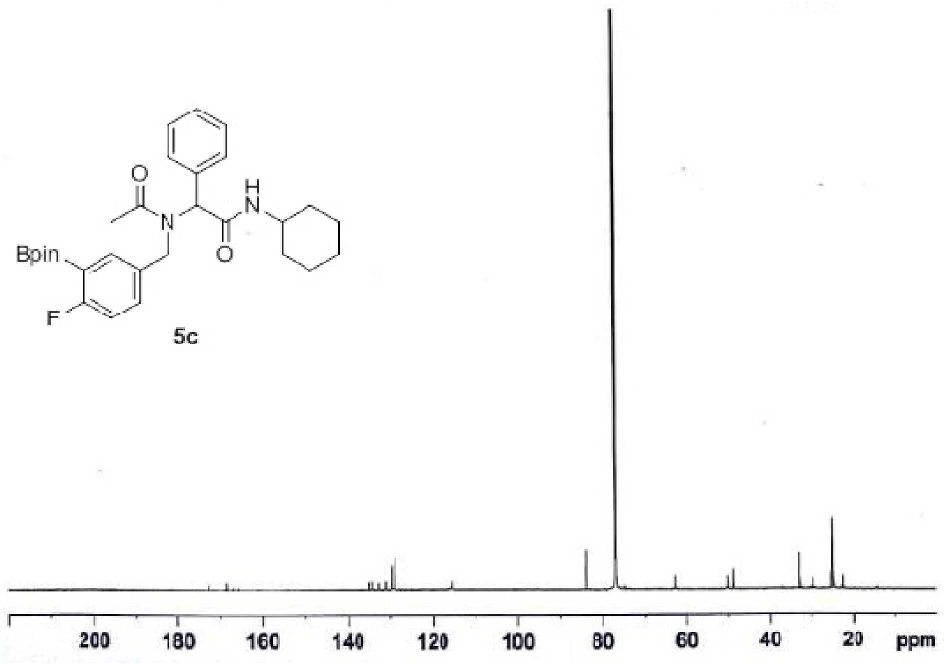


150.9 MHz $^{13}$C-NMR of Compound (**5c**) in $CDCl_3$

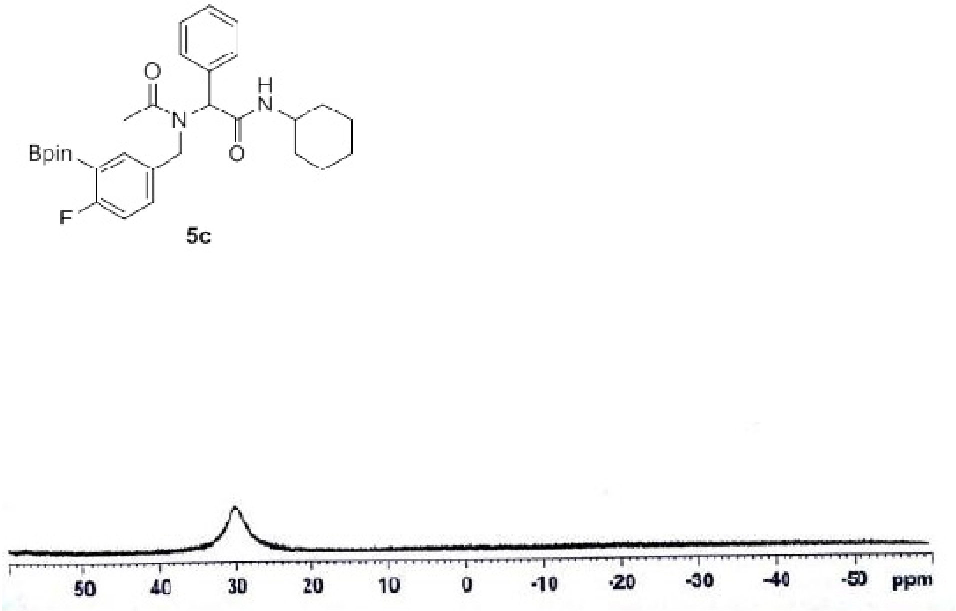


192.5 MHz $^{11}$B-NMR of Compound (**5c**) in $CDCl_3$

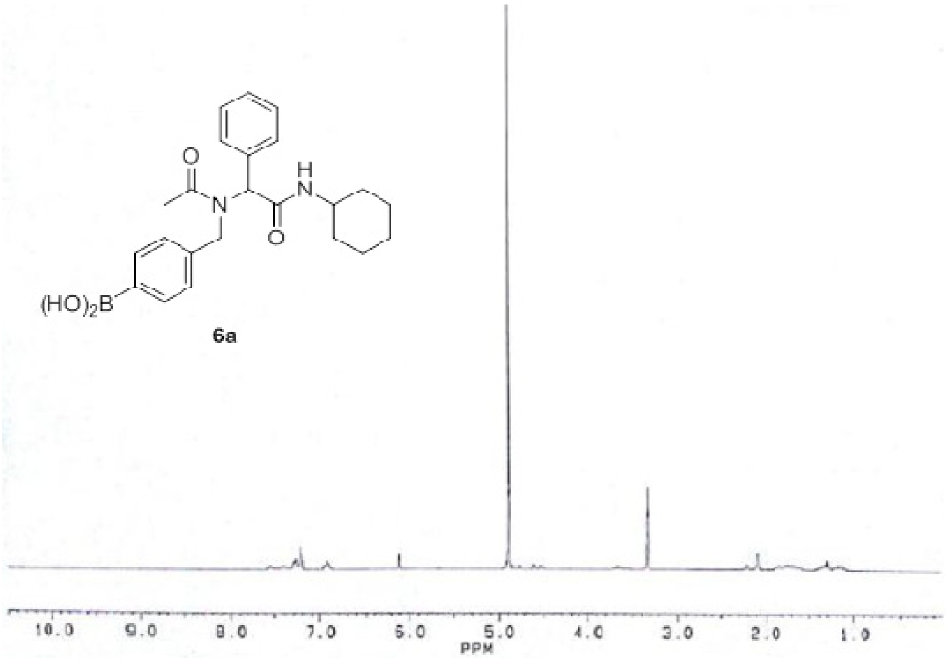


300 MHz $^{1}$H-NMR of Compound (**6a**) in $CD_3OD$-$d_4$

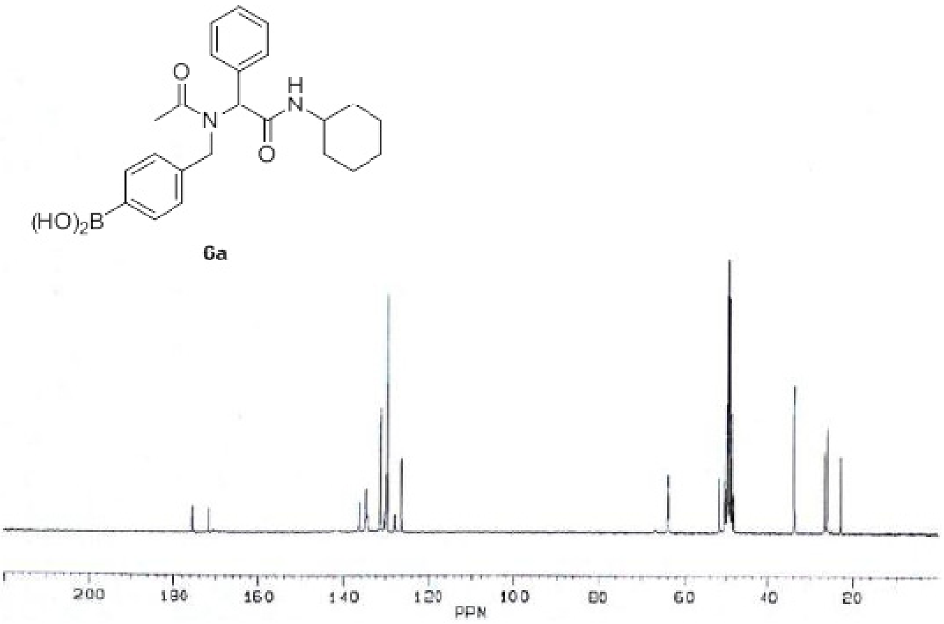


75.5 MHz $^{13}$C-NMR of Compound (**6a**) in $CD_3OD$-$d_4$

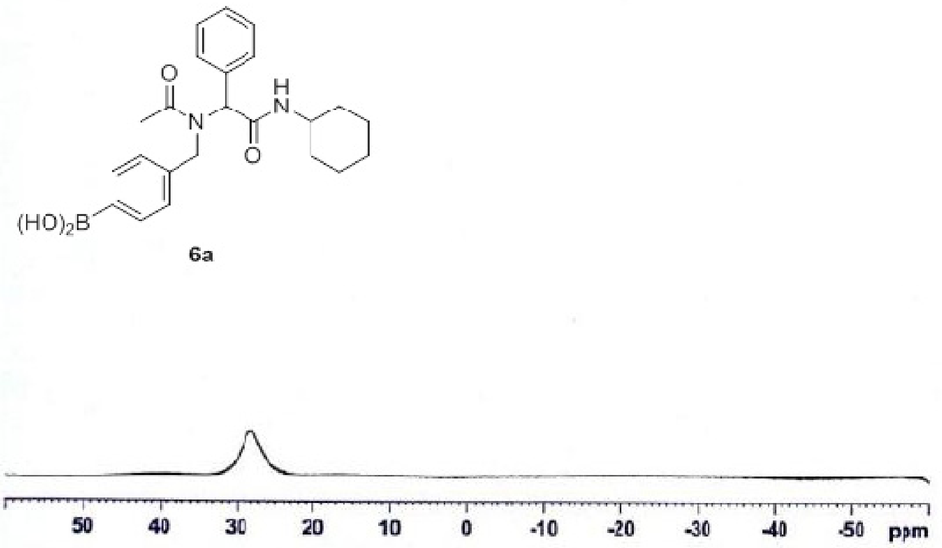


192.5MHz $^{11}$B-NMR of Compound (**6a**) in $CD_3OD$-*d*$_4$

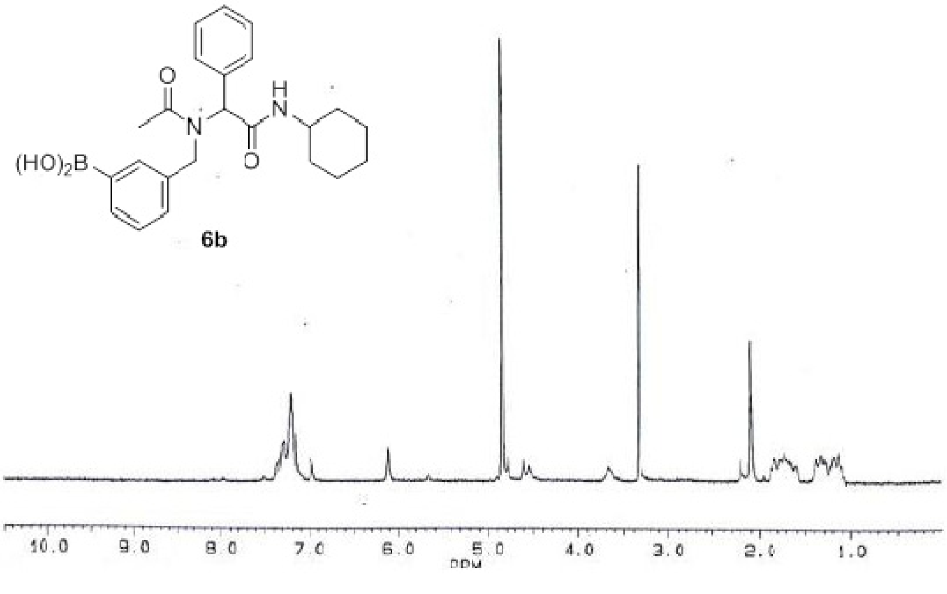


300 MHz $^1$H-NMR of Compound (**6b**) in $CD_3OD$-*d*$_4$

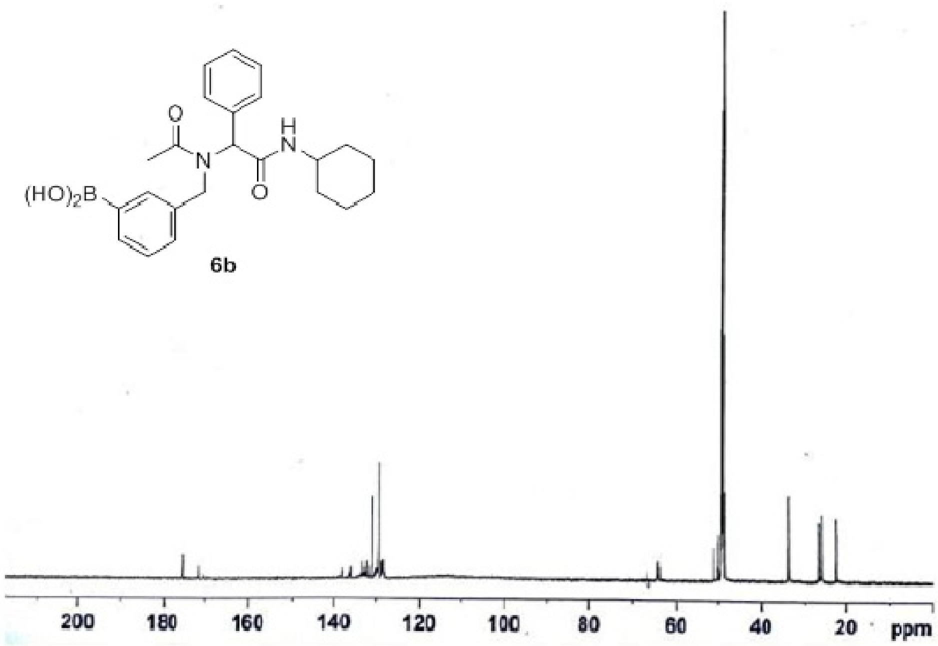


150.9 MHz $^{13}$C-NMR of Compound (**6b**) in $CD_3OD$-$d_4$

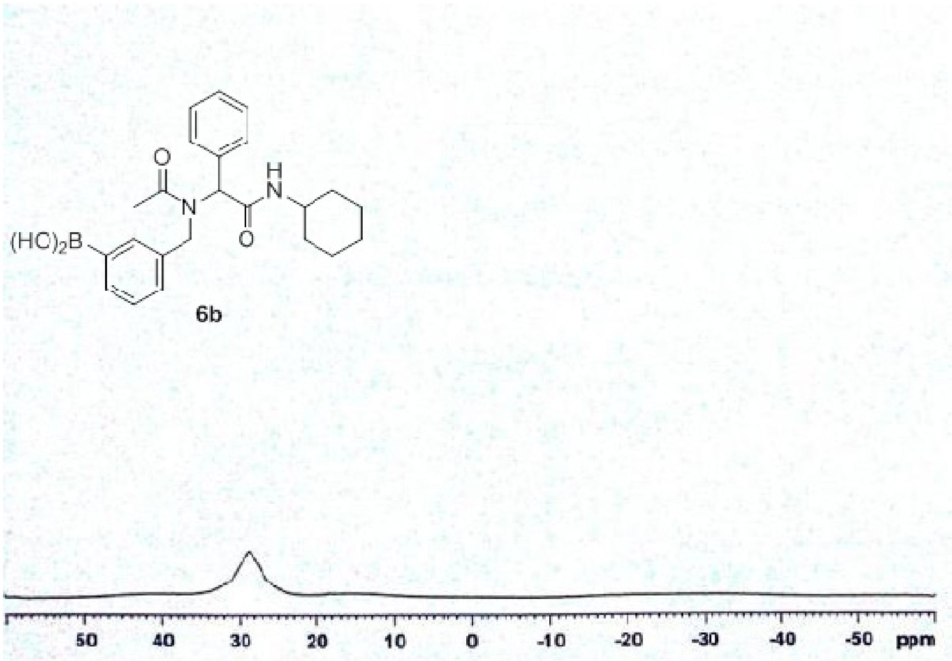


192.5 MHz $^{11}$B-NMR of Compound (**6b**) in $CD_3OD$-$d_4$

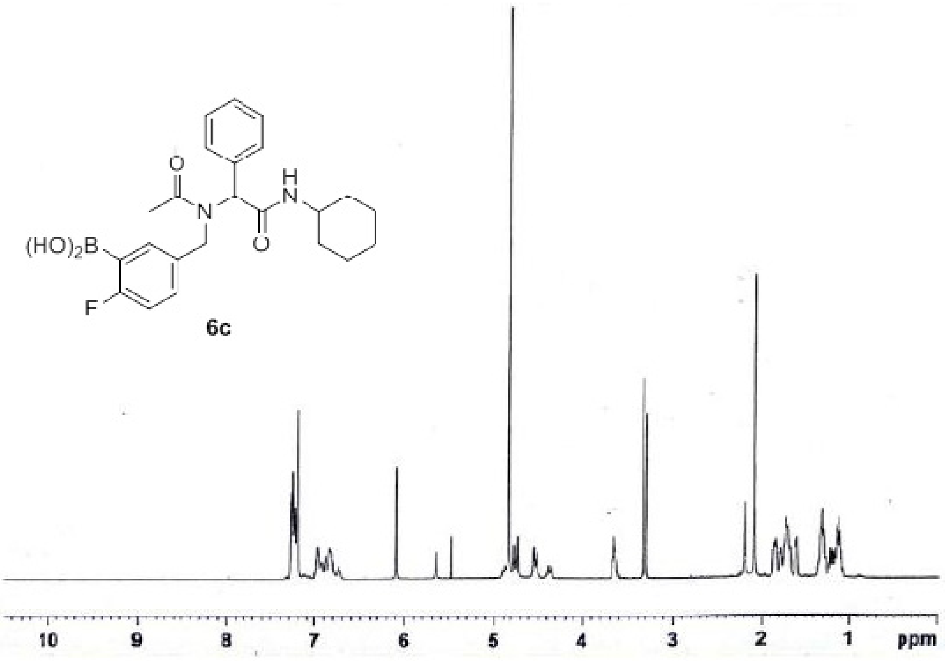


600 MHz $^{1}$H-NMR of Compound (**6c**) in $CD_3OD$-$d_4$

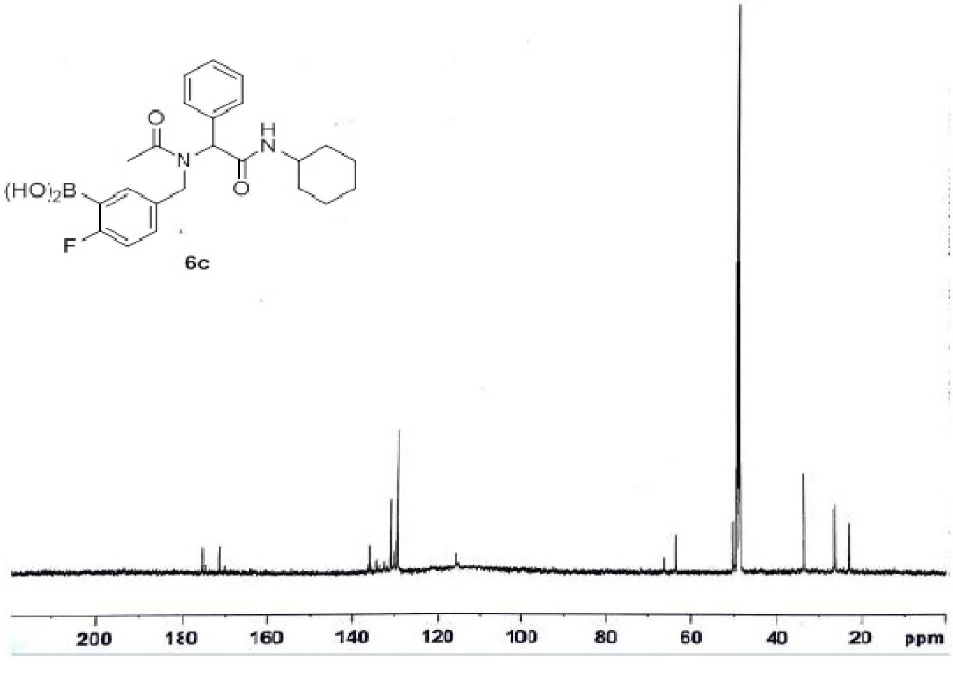


75.5 MHz $^{13}$C-NMR of Compound (**6c**) in $CD_3OD$-$d_4$

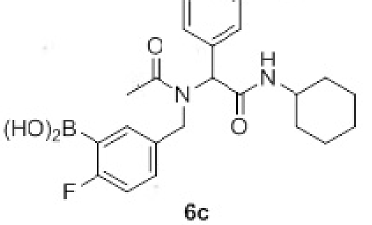

**6c**

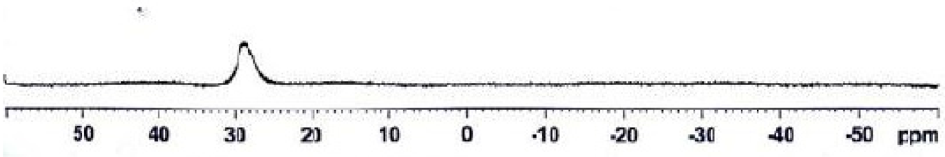


192.5 MHz $^{11}$B-NMR of Compound (**6c**) in $CD_3OD$-$d_4$

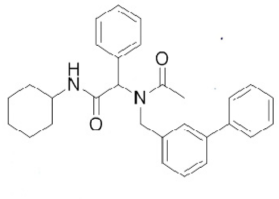

8a

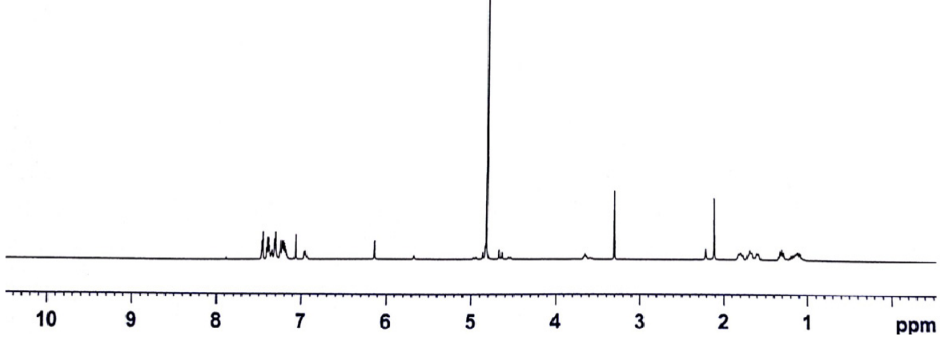


600 MHz $^{1}$H-NMR of Compound (**8a**) in $CD_3OD$-$d_4$

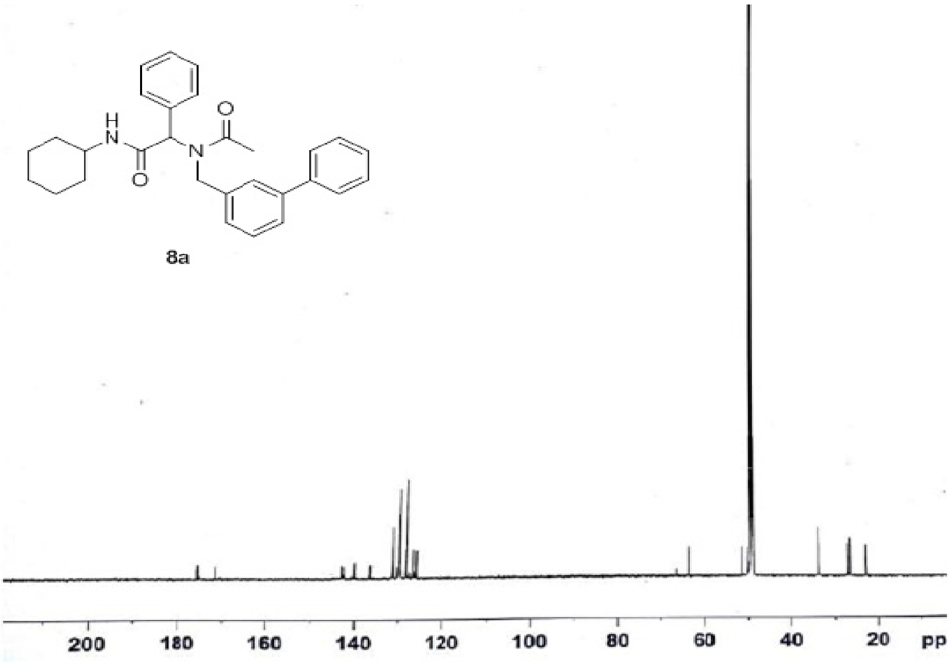


150.9 MHz $^{13}$C-NMR of Compound (**8a**) in $CD_3OD$-$d_4$

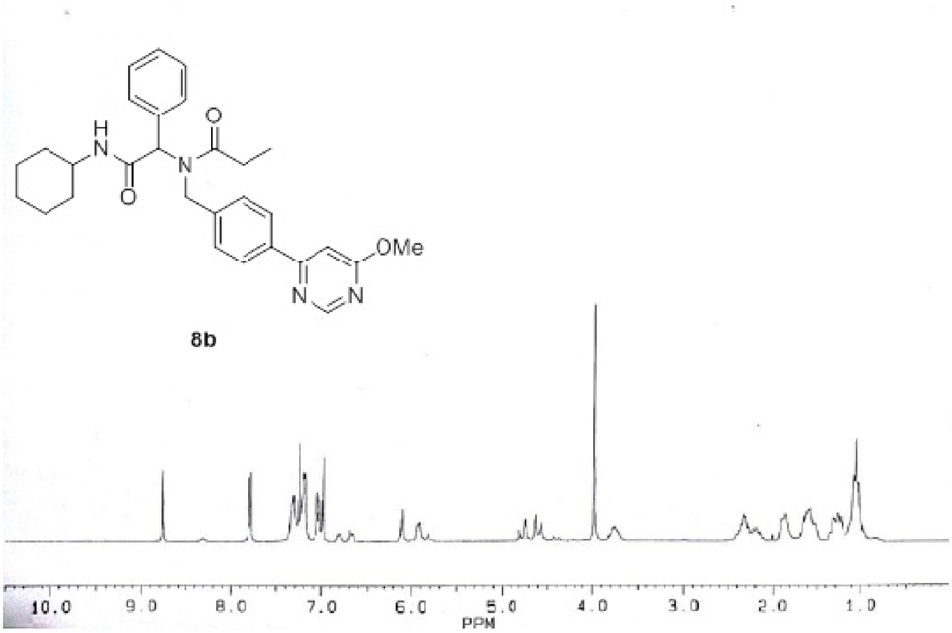


300 MHz $^{1}$H-NMR of Compound (**8b**) in $CDCl_3$

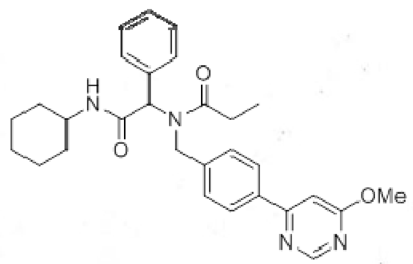


**8b**

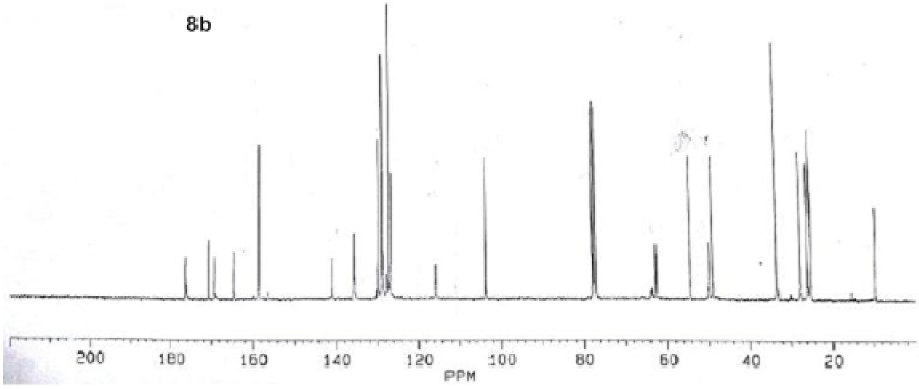


75.5 MHz $^{13}$C-NMR of Compound (**8b**) in $CDCl_3$
